# Supplementary material for: Simple and Expedient Access to Novel Fluorinated Thiazolo- and Oxazolo[3,2-a]pyrimidin-7-one Derivatives and Their Functionalization via Palladium-Catalyzed Reactions
Source: Molecules. 2022 May 7;27(9):3013. doi: 10.3390/molecules27093013 (PMC9100779; doi:10.3390/molecules27093013)

Simple and expedient access to novel fluorinated thiazolo- and oxazolo[3,2-*a*]pyrimidin-7-one derivatives and their functionalization *via* palladium-catalyzed reactions.

Wafa Blancou,[1,2] Badr Jismy,[1,*] Soufiane Touil,[2] Hassan Allouchi[3] and Mohamed Abarbri[1,*]

[1] Laboratoire de Physico-Chimie des Matériaux et des Electrolytes pour l'Energie (PCM2E), EA 6299, Avenue Monge, Faculté des Sciences, Université de Tours, Parc de Grandmont, 37200 Tours, France.
[2] Laboratoire des Composés Hétéro-Organiques et Matériaux Nanostructurés (LR18ES11), Université de Carthage, Faculté des Sciences de Bizerte, 7021 Zarzouna, Tunisie.
[3] Université de Tours, Faculté de Pharmacie, EA 7502 SIMBA, 31 avenue Monge - 37200 Tours, France.

* Correspondence: mohamed.abarbri@univ-tours.fr and badr.jismy@hotmail.com ; Tel.: +33(2)47-36-73-59; Fax: +33(2)47-36-70-73

5-(trifluoromethyl)-7H-thiazolo[3,2-a]pyrimidin-7-one (**3a**).

^{1}H NMR (300 MHz, $CDCl_3$)

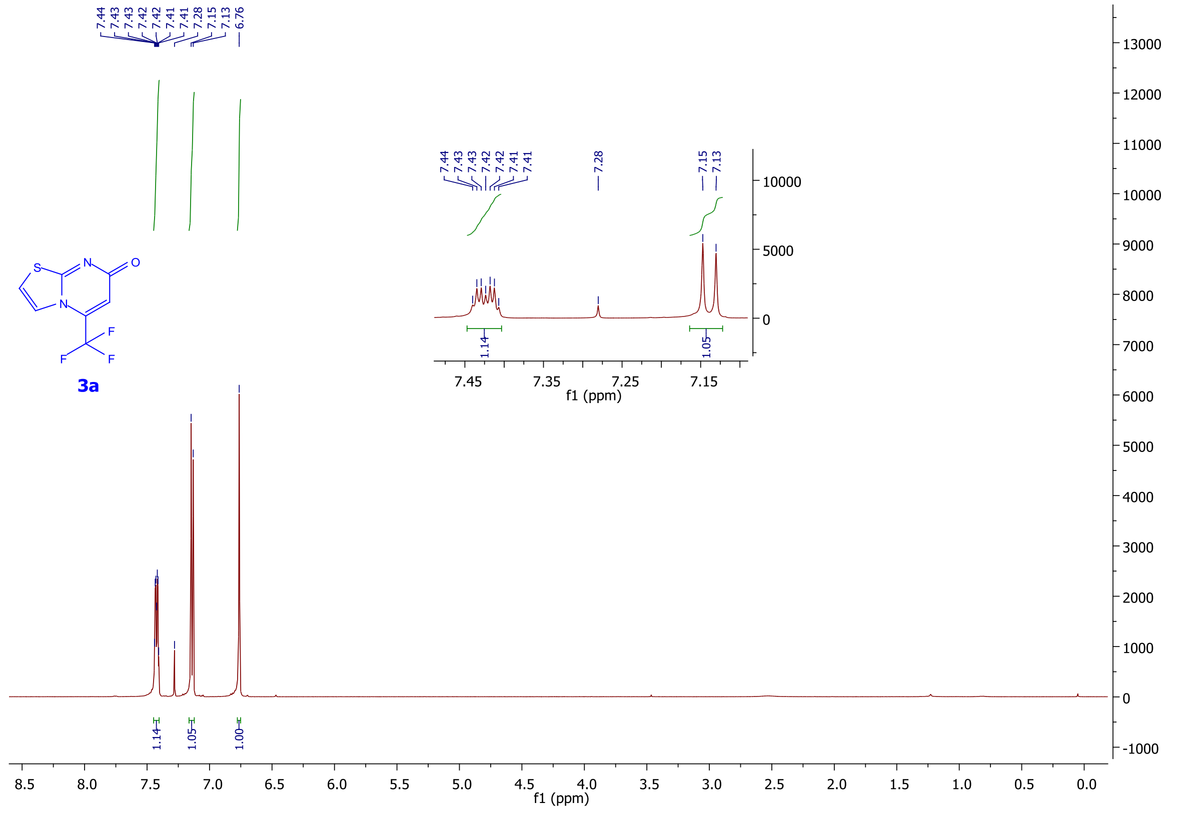

^{19}F NMR (282 MHz, $CDCl_3$)

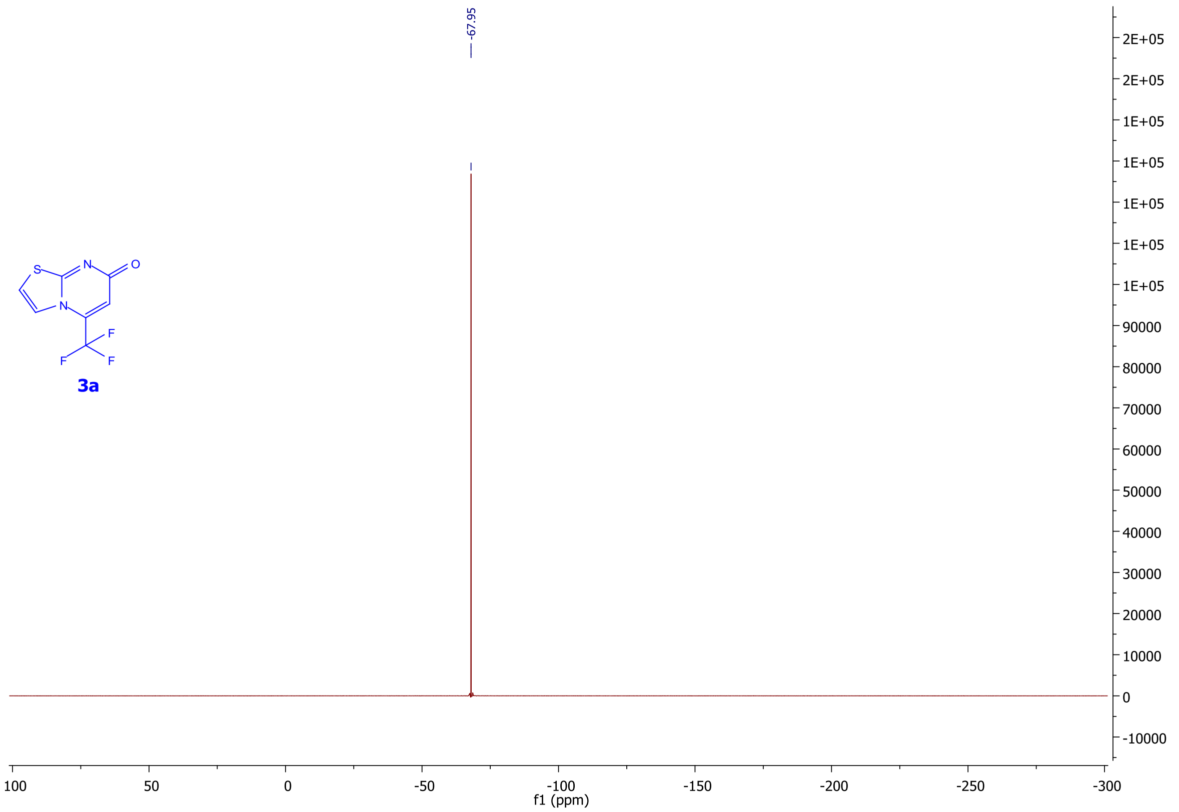

^{13}C NMR (75 MHz, $CDCl_3$)

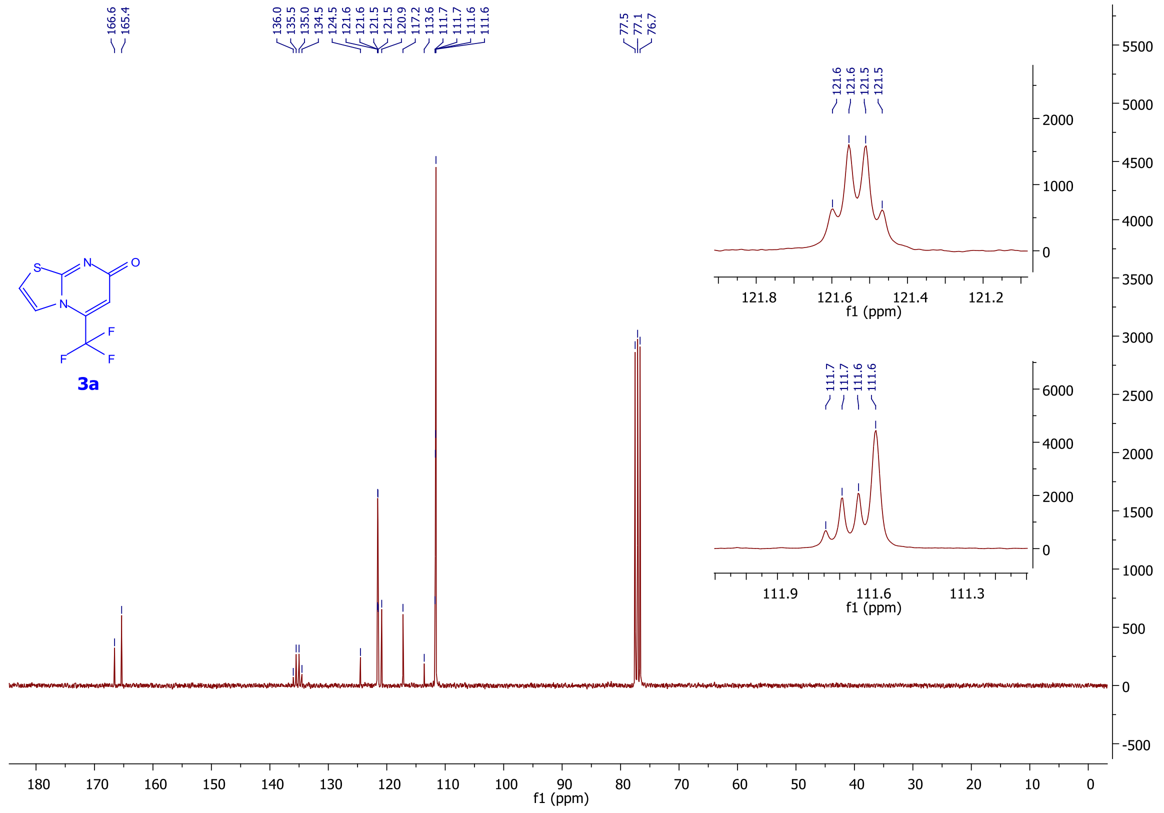

5-(perfluoroethyl)-7H-thiazolo[3,2-a]pyrimidin-7-one (**3b**).

^{1}H NMR (300 MHz, $CDCl_3$)

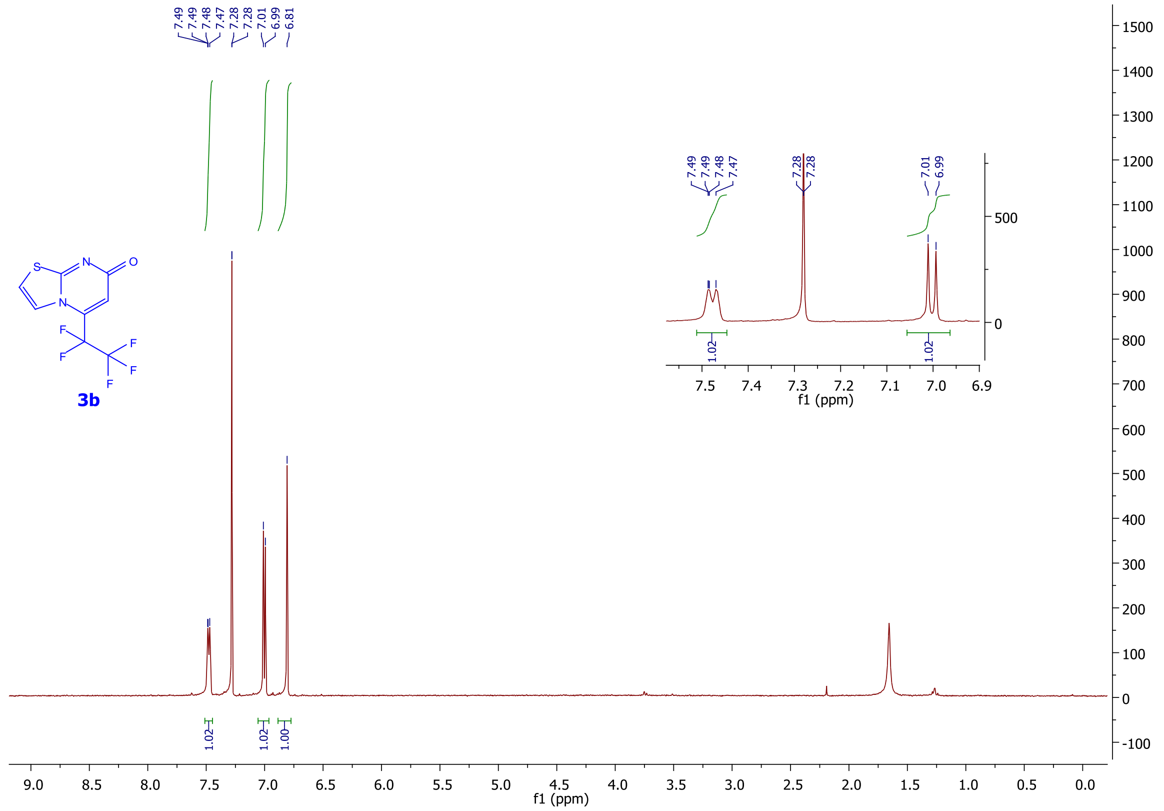

^{19}F NMR (282 MHz, $CDCl_3$)

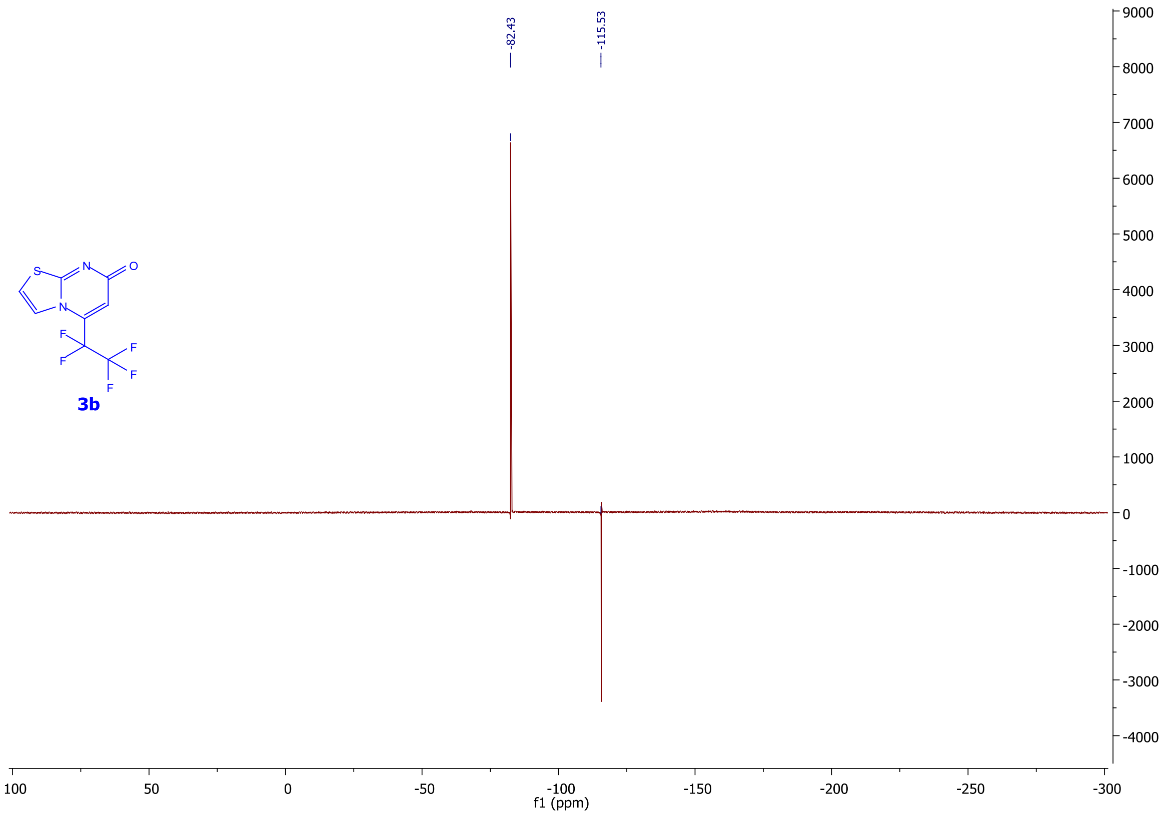

^{13}C NMR (75 MHz, $CDCl_3$)

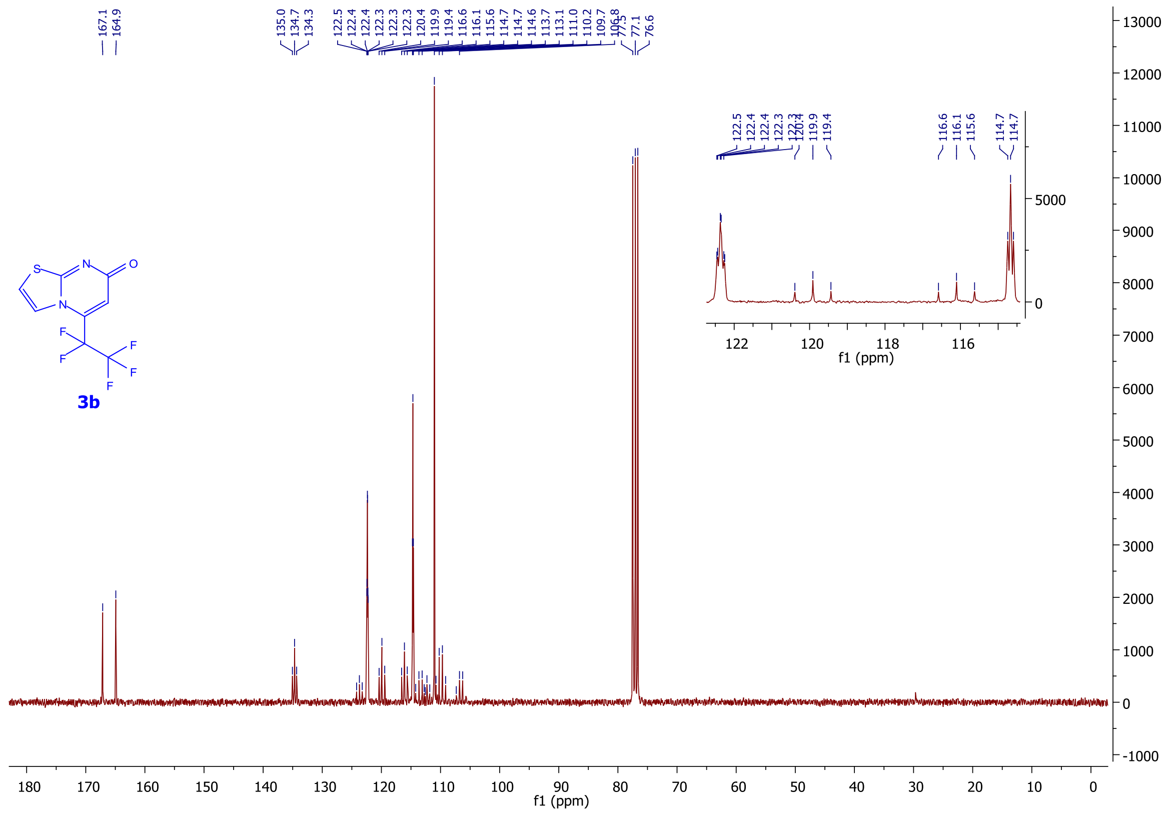

5-(difluoro(4-methoxyphenyl)methyl)-7H-thiazolo[3,2-a]pyrimidin-7-one (**3c**).

^{1}H NMR (300 MHz, $CDCl_3$)

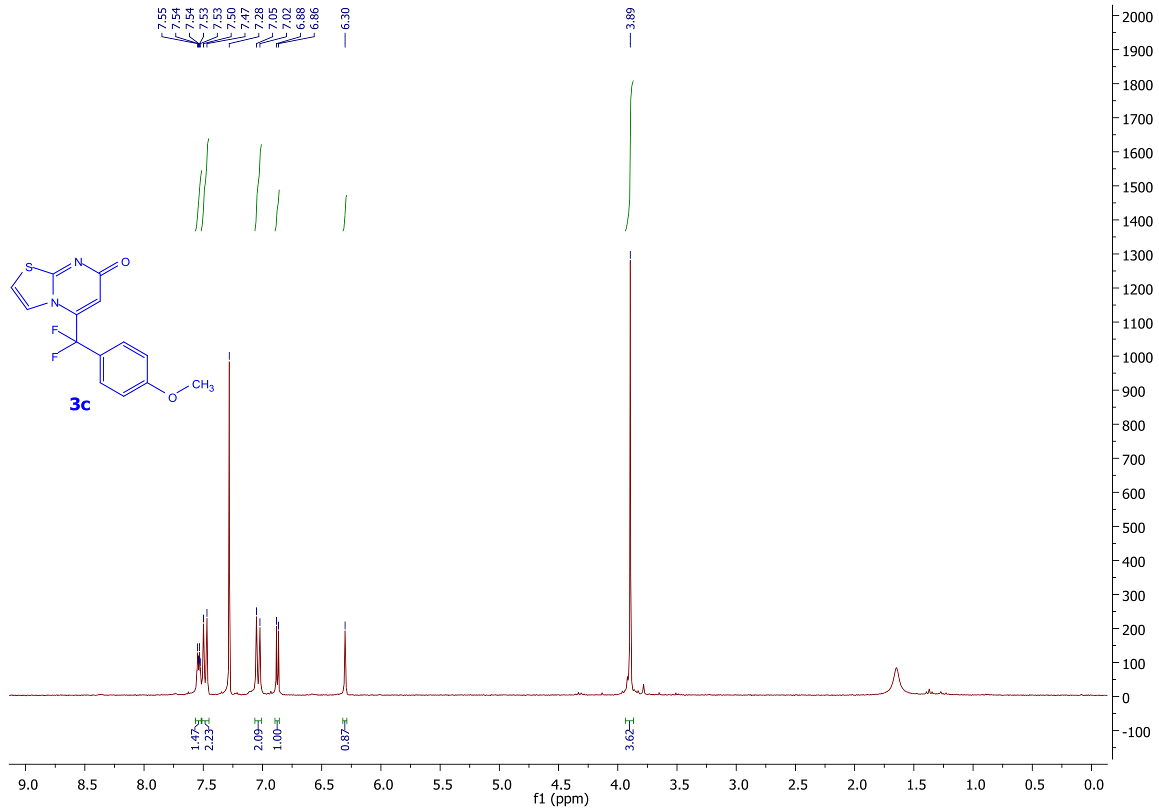

^{19}F NMR (282 MHz, $CDCl_3$)

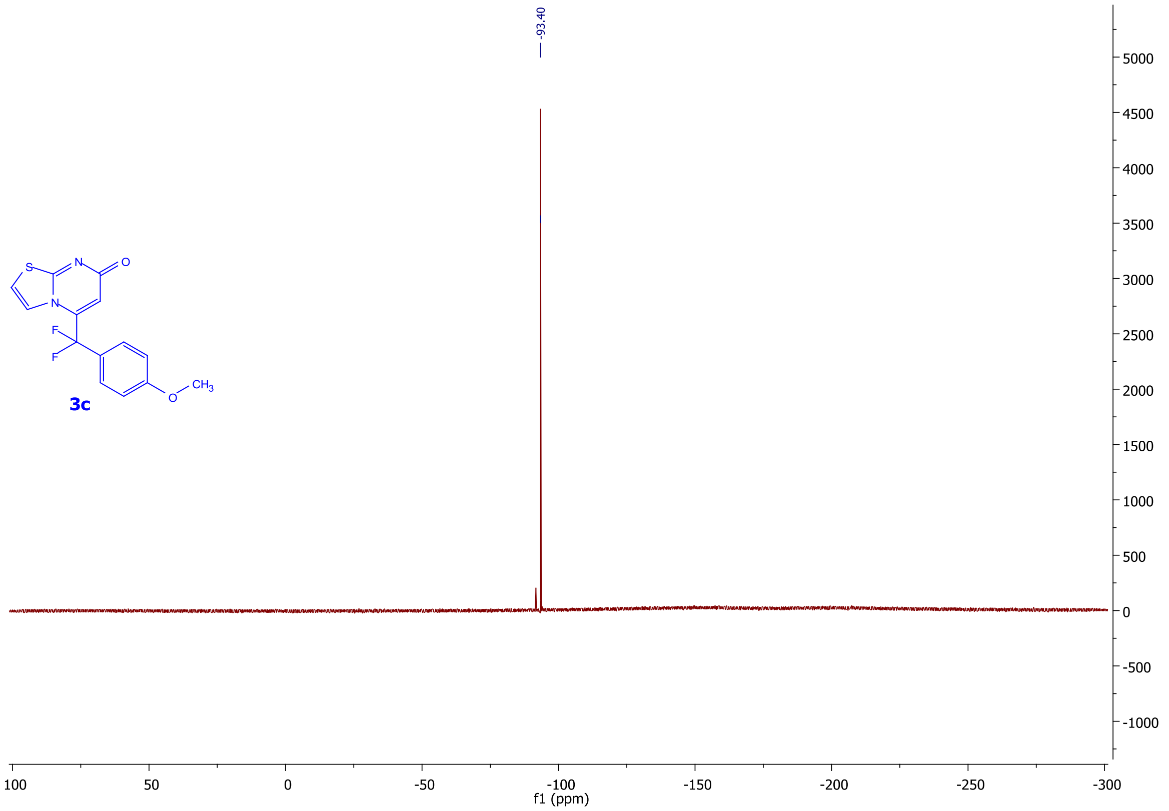

^{13}C NMR (75 MHz, $CDCl_3$)

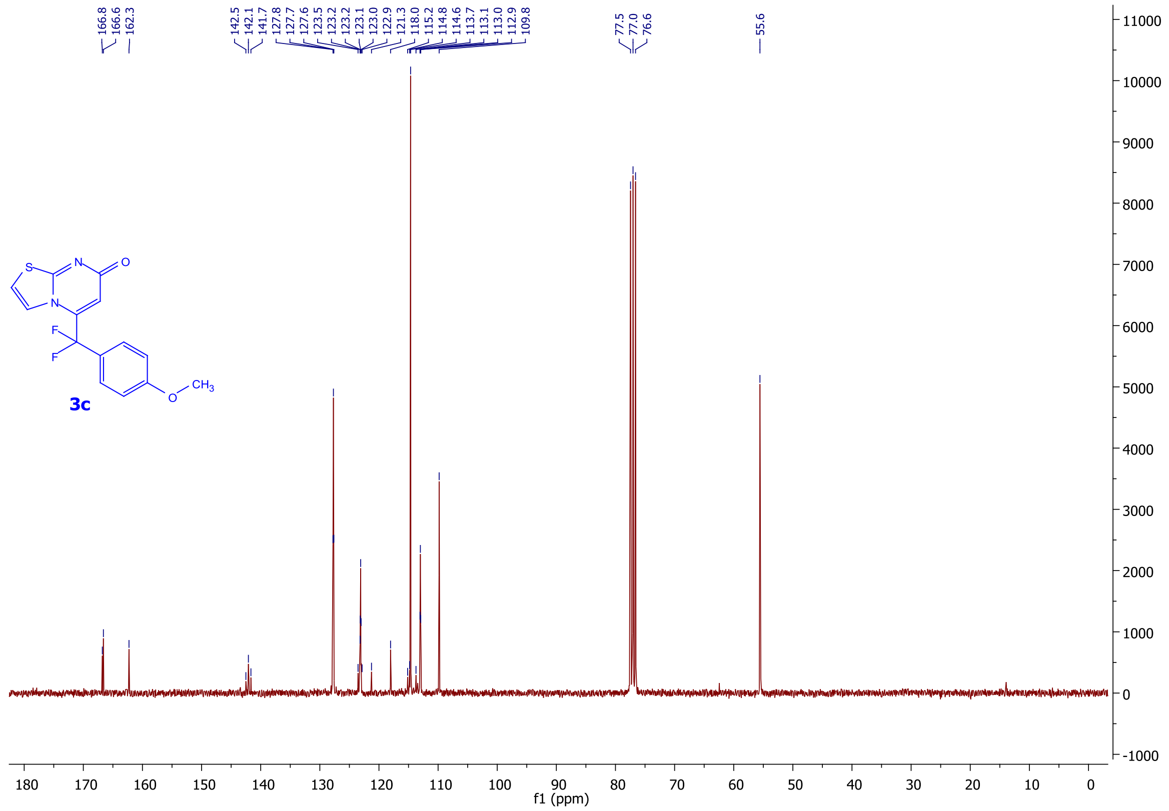

5-((4-bromophenyl)difluoromethyl)-7H-thiazolo[3,2-a]pyrimidin-7-one (**3d**).

^{1}H NMR (300 MHz, $CDCl_3$)

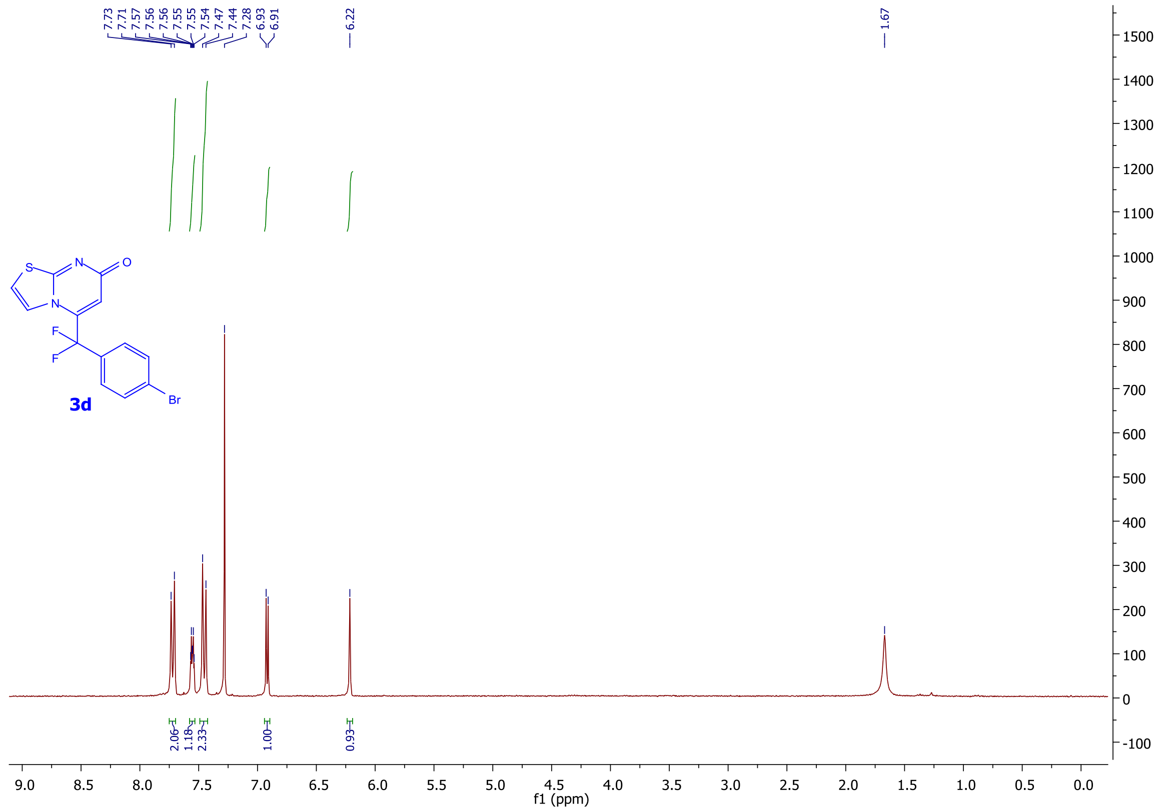

^{19}F NMR (282 MHz, $CDCl_3$)

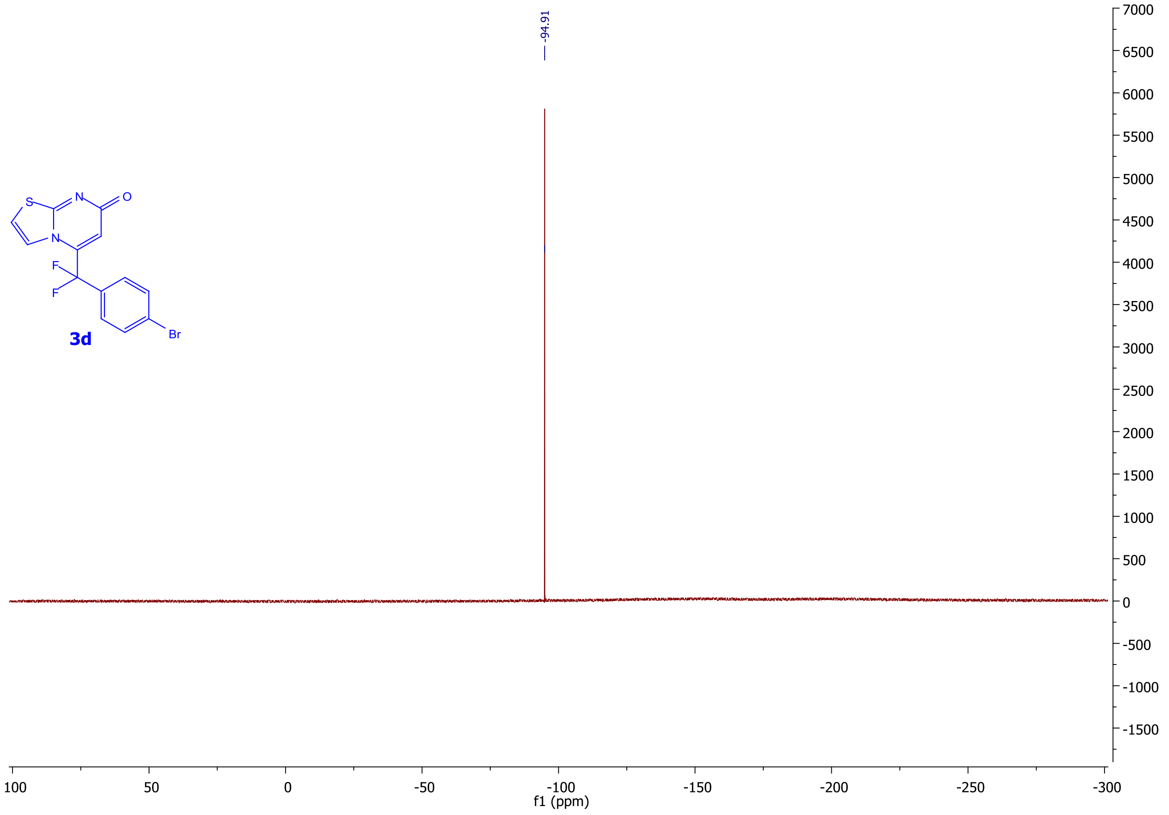

^{13}C NMR (75 MHz, $CDCl_3$)

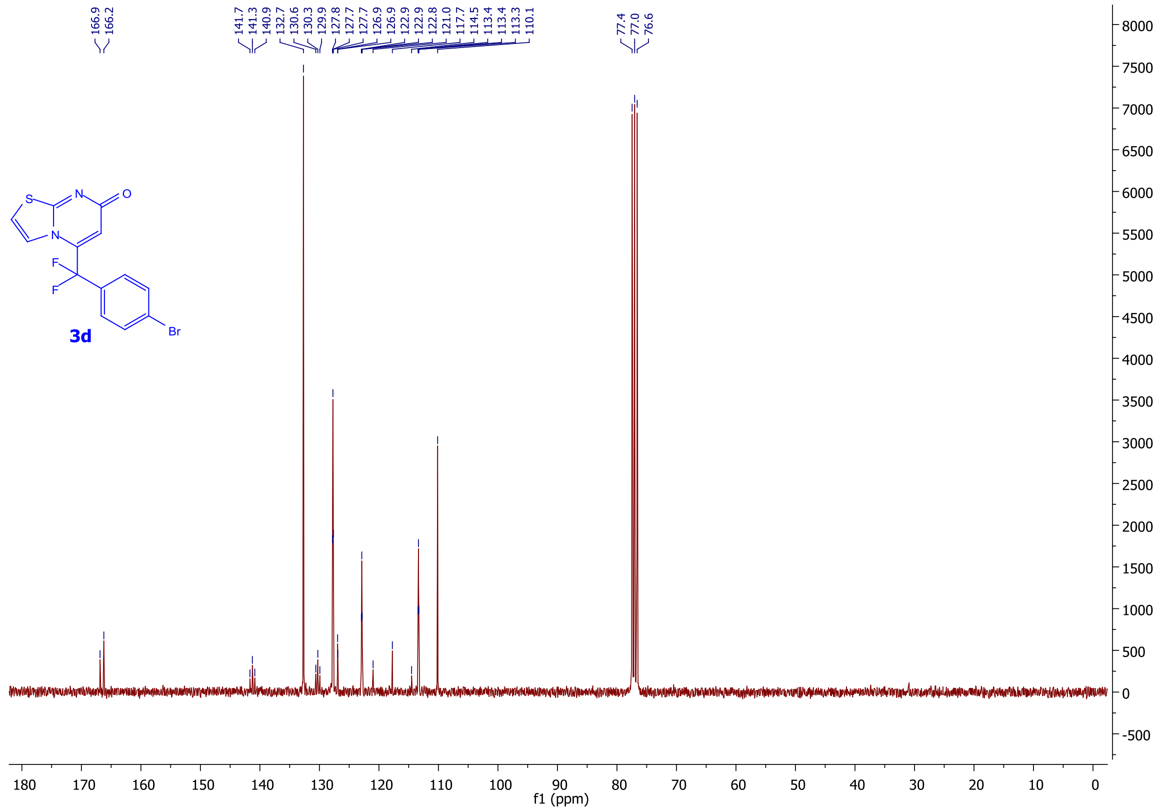

2-bromo-5-(trifluoromethyl)-7H-thiazolo[3,2-a]pyrimidin-7-one (**3e**).

^{1}H NMR (300 MHz, DMSO-d_6)

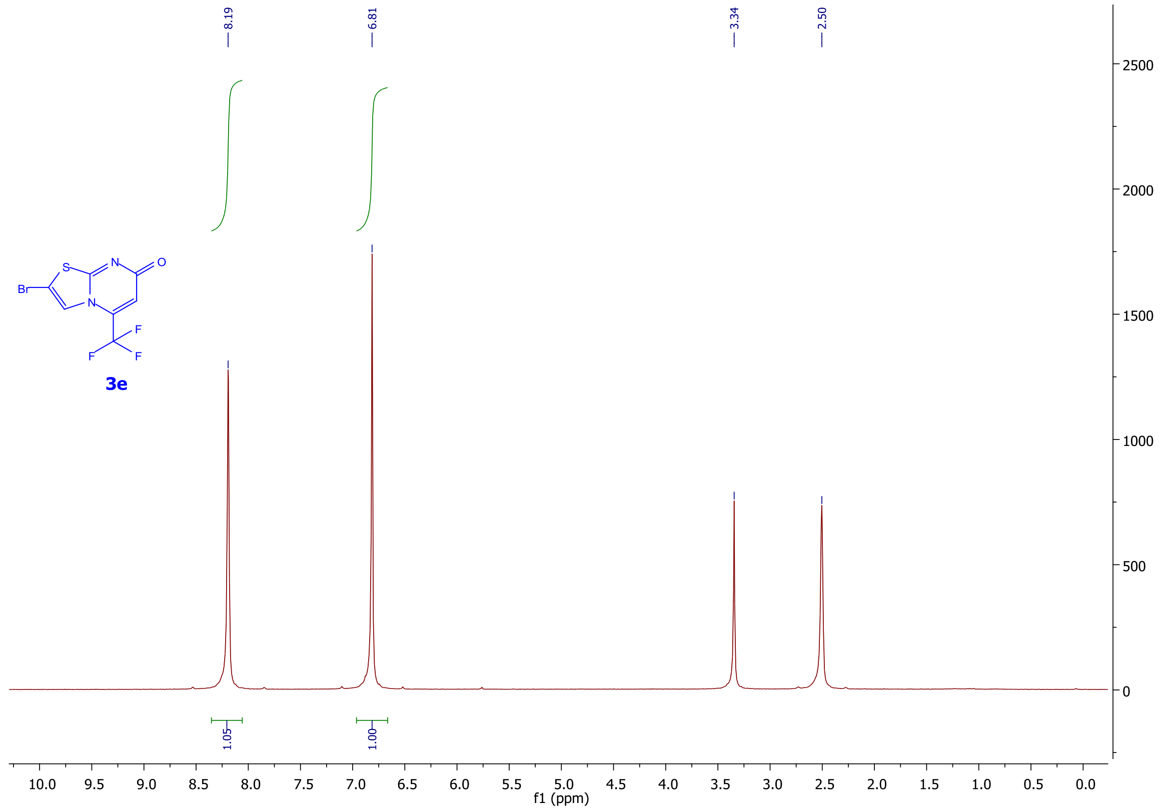

^{19}F NMR (282 MHz, DMSO-d_6)

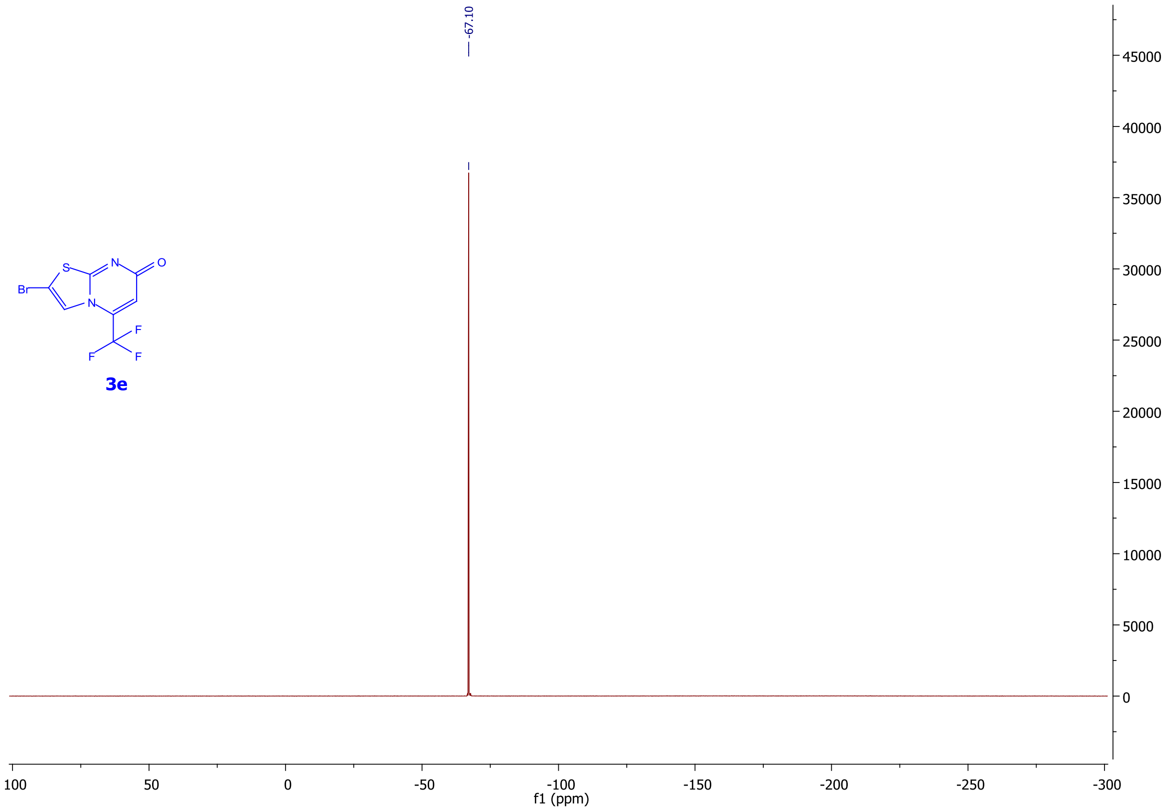

^{13}C NMR (75 MHz, DMSO-d_6)

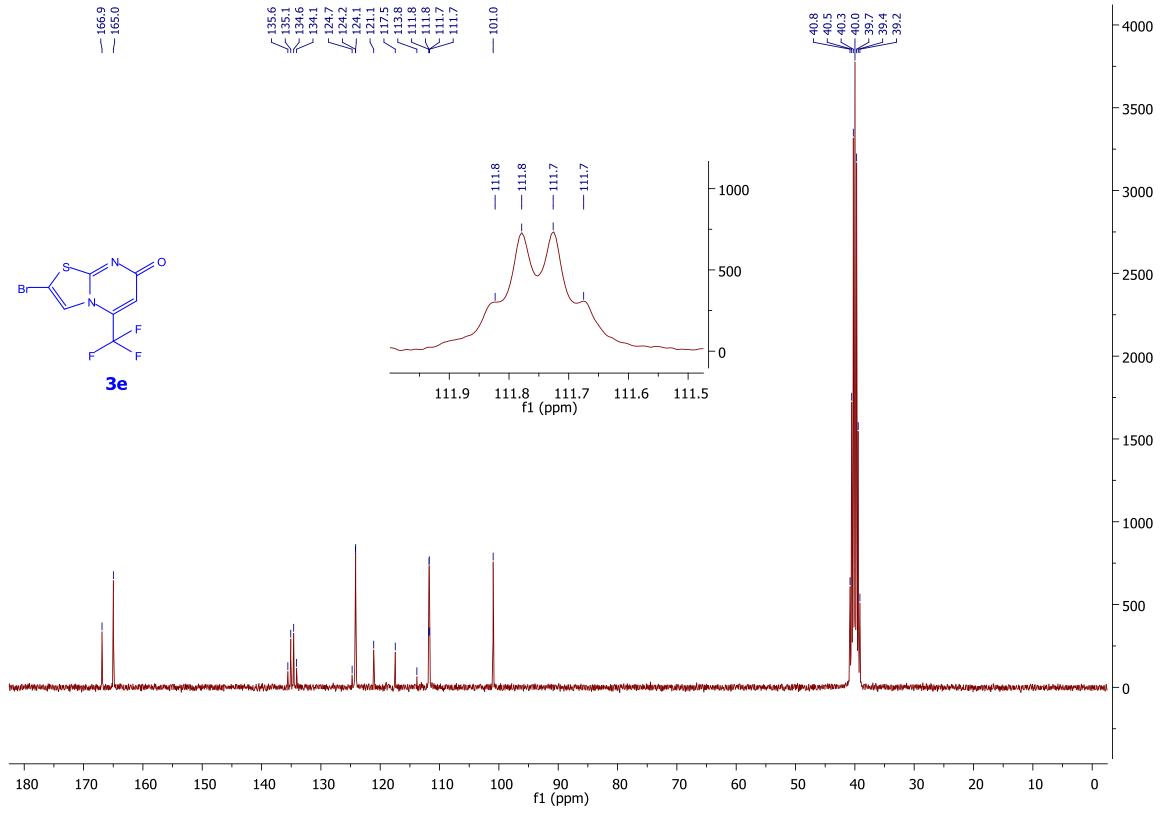

2-bromo-5-(perfluoroethyl)-7H-thiazolo[3,2-a]pyrimidin-7-one (**3f**)

^{1}H NMR (300 MHz, $CDCl_3$)

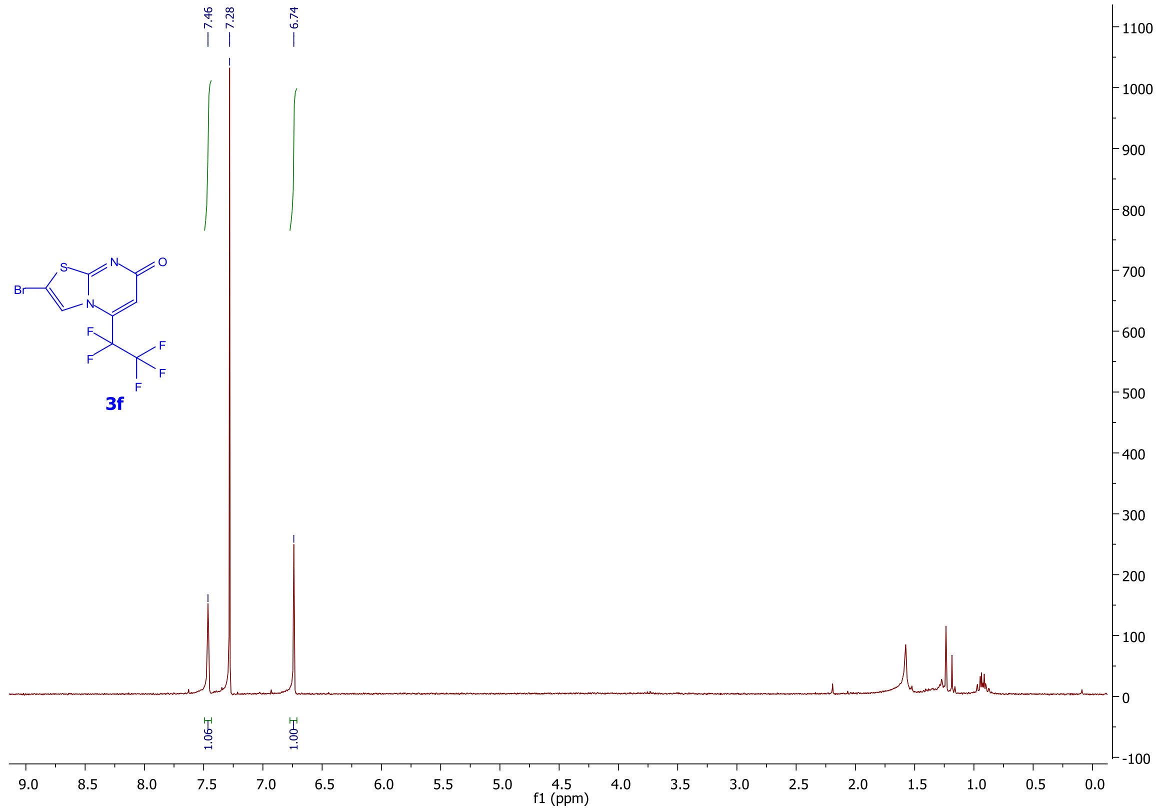

^{19}F NMR (282 MHz, $CDCl_3$)

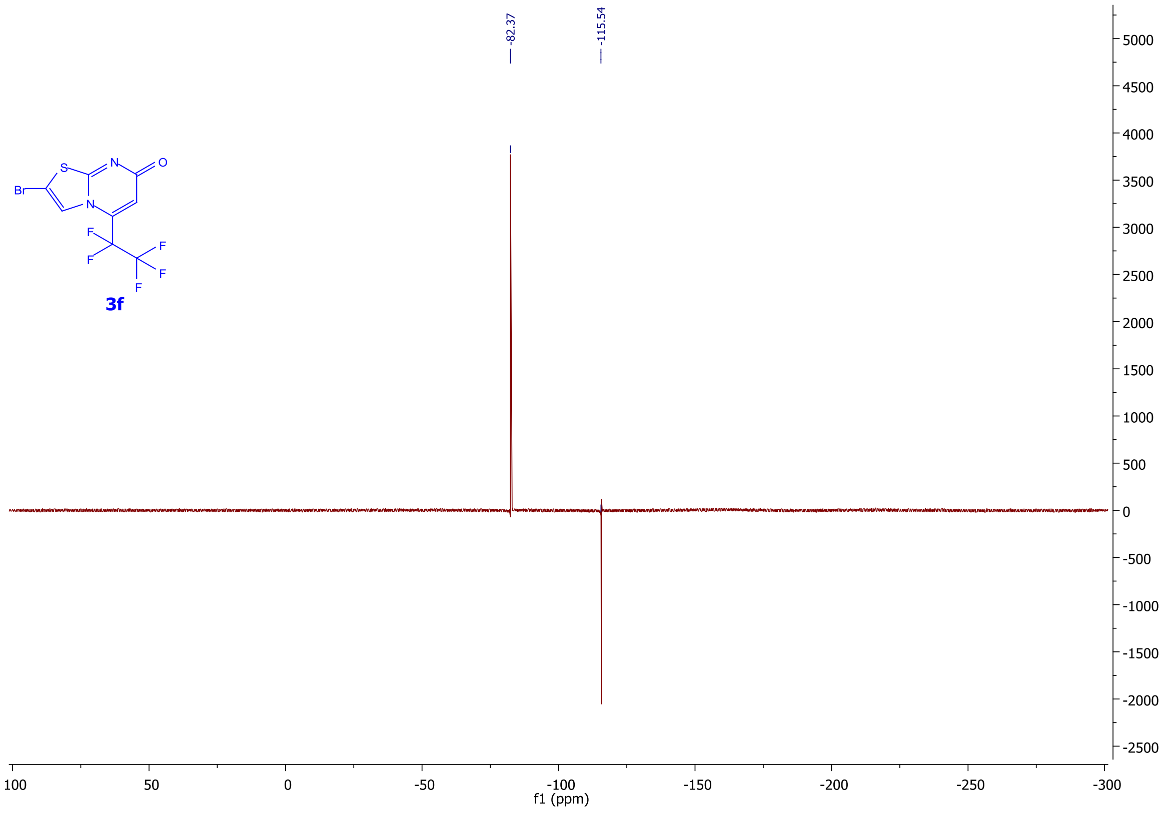

^{13}C NMR (75 MHz, $CDCl_3$)

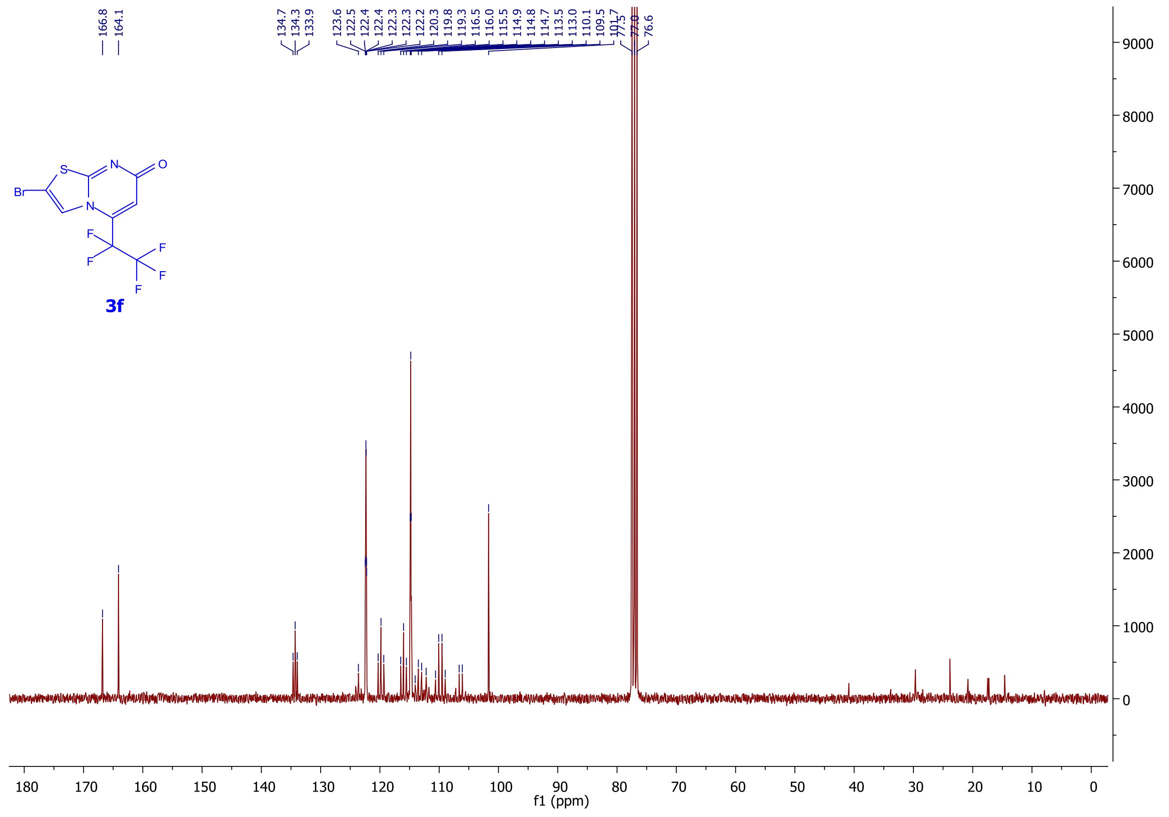

2-bromo-5-((4-bromophenyl)difluoromethyl)-7H-thiazolo[3,2-a]pyrimidin-7-one (**3g**).

^{1}H NMR (300 MHz, $CDCl_3$)

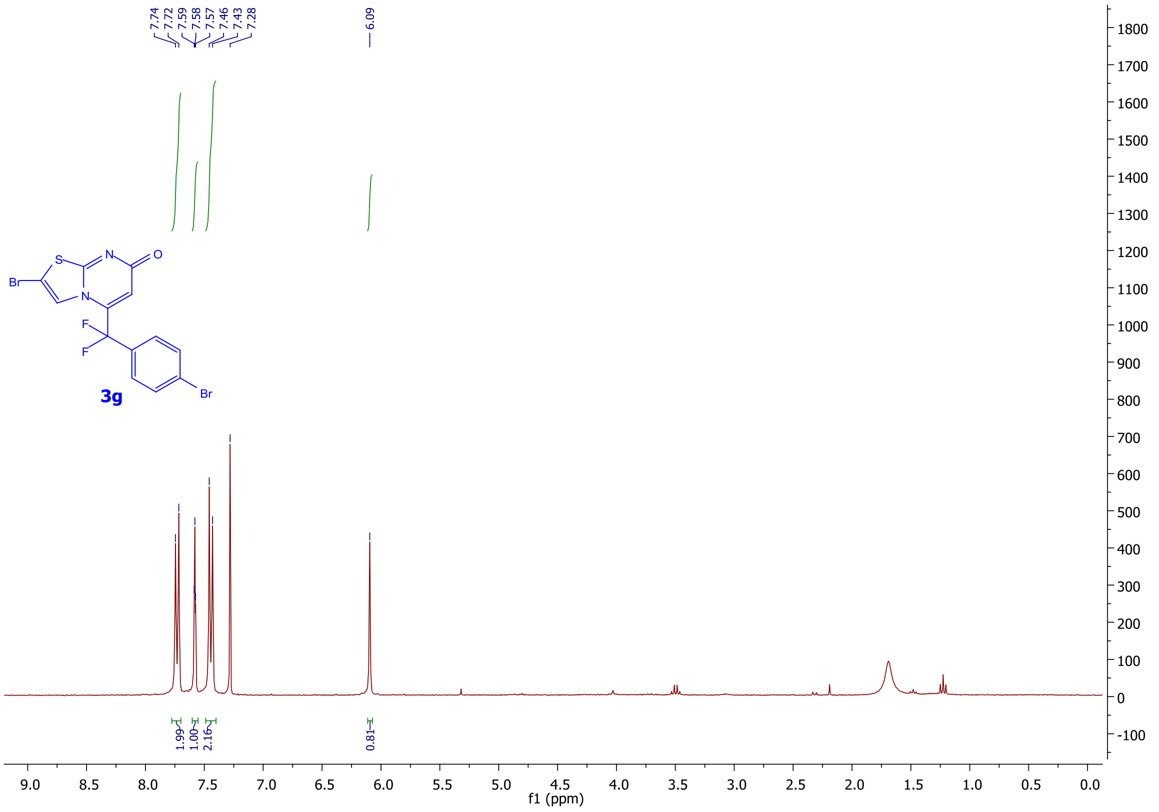

^{19}F NMR (282 MHz, $CDCl_3$)

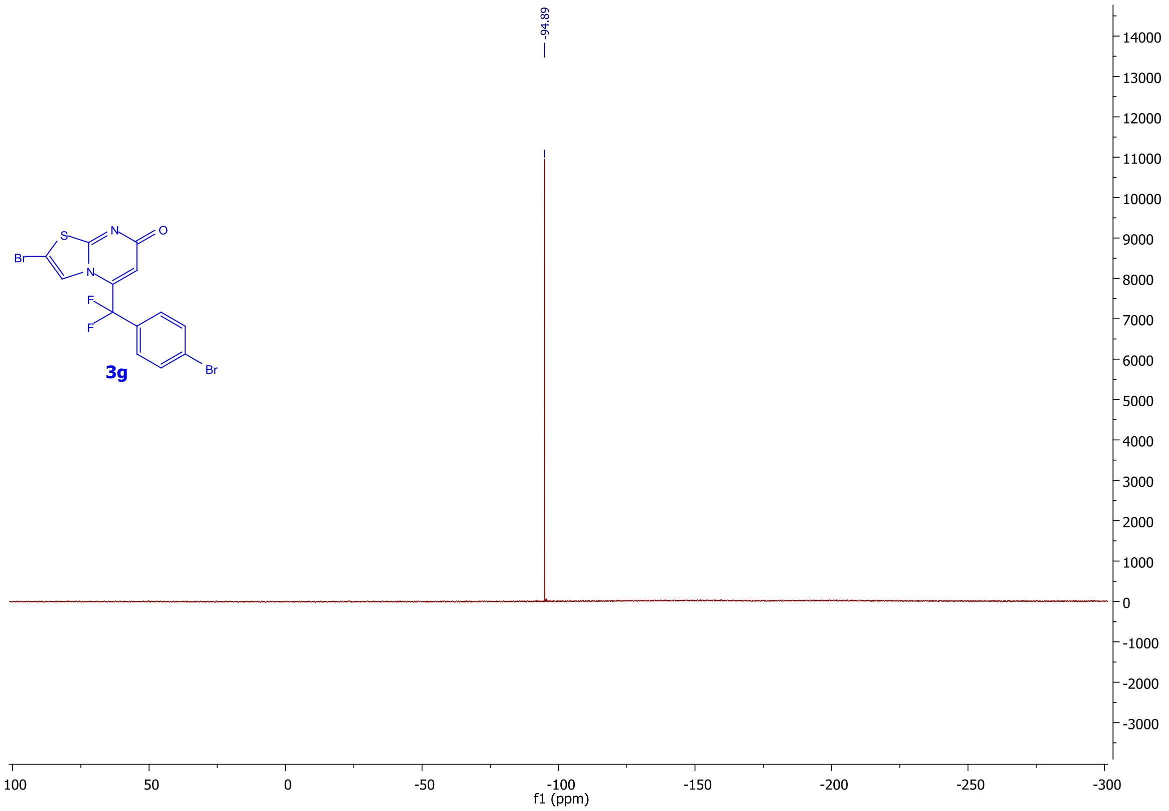

^{13}C NMR (75 MHz, $CDCl_3$)

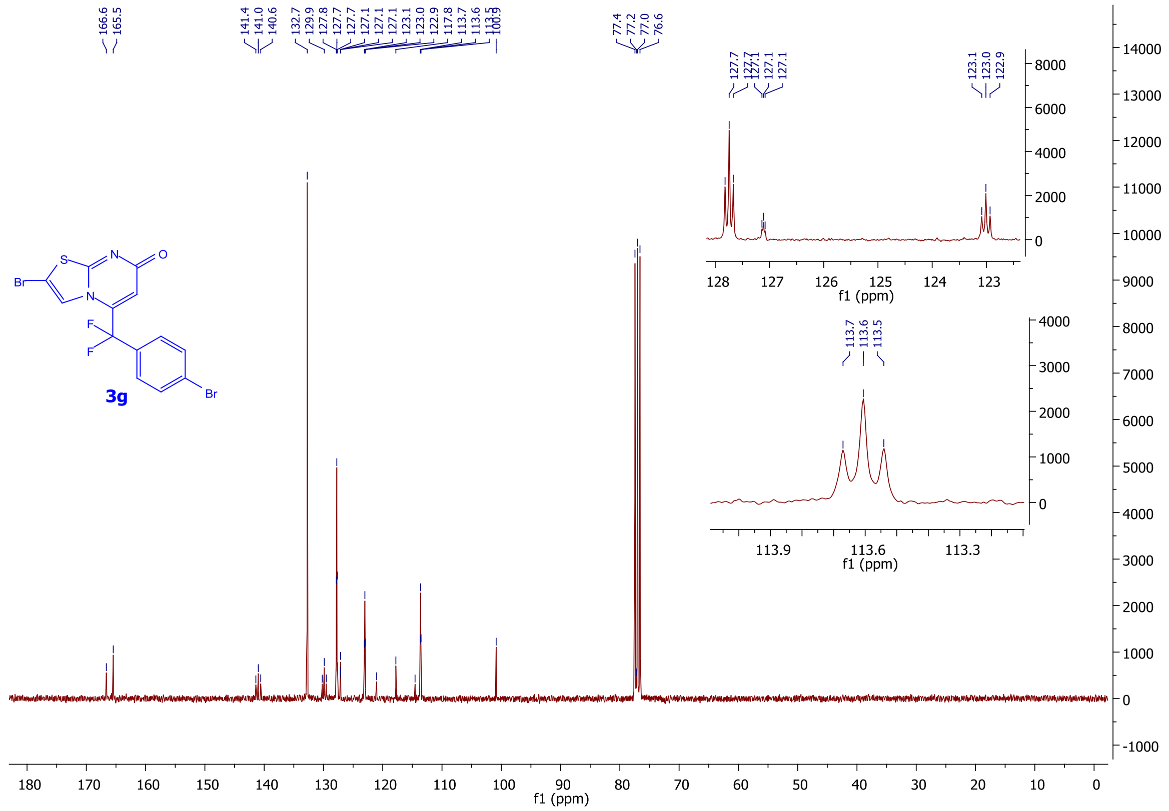

4-(trifluoromethyl)-2H-benzo[4,5]thiazolo[3,2-a]pyrimidin-2-one (**3h**).

^{1}H NMR (300 MHz, $CDCl_3$)

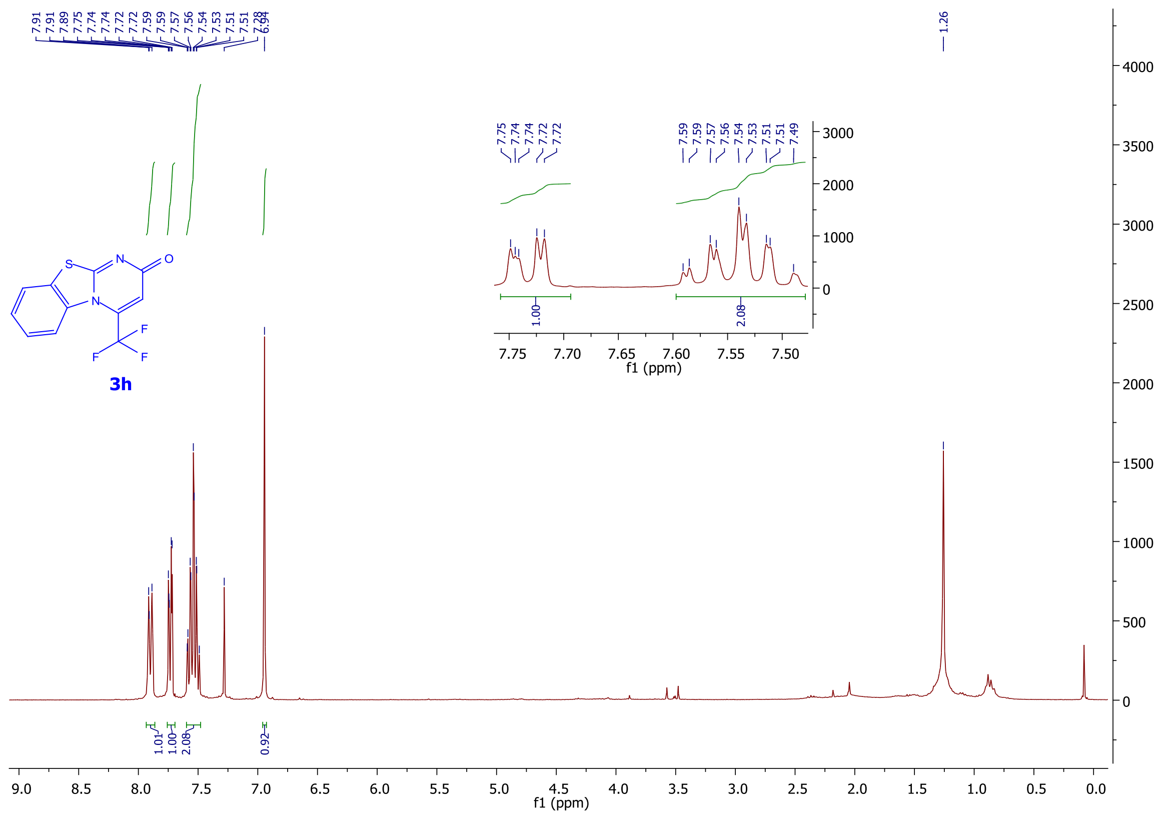

^{19}F NMR (282 MHz, $CDCl_3$)

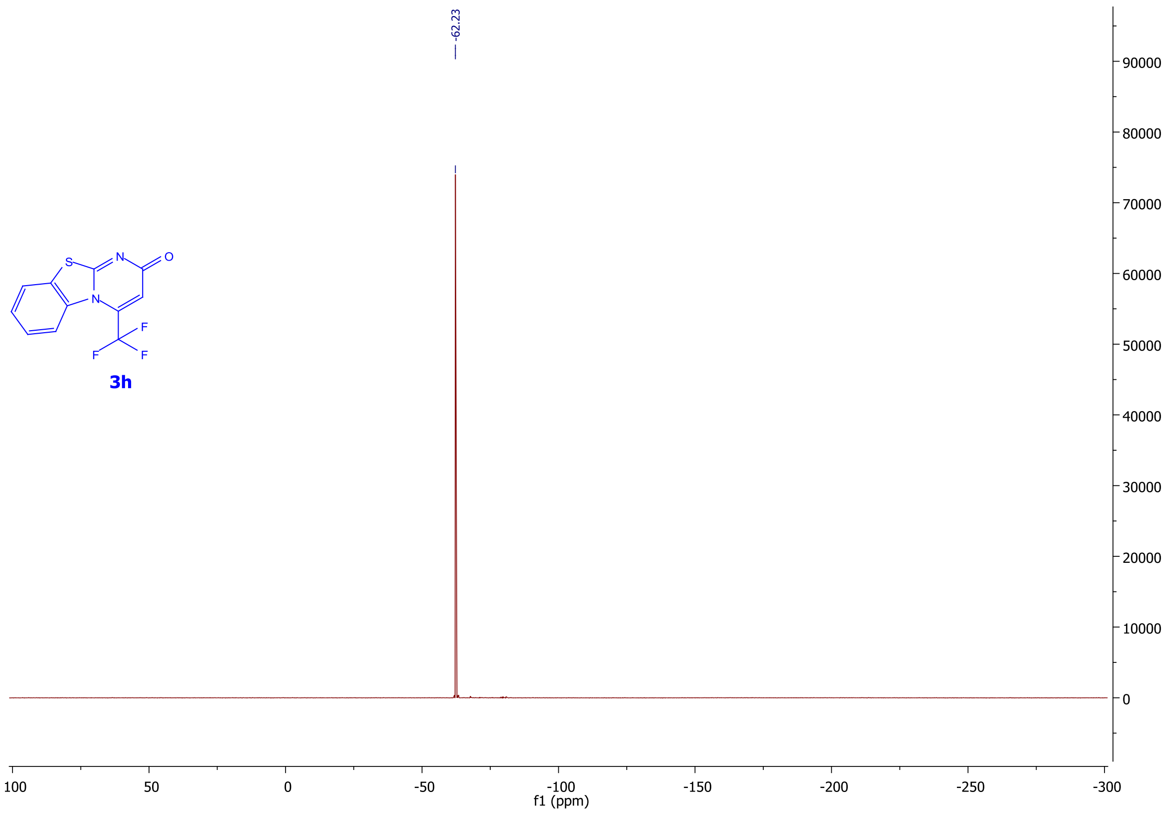

^{13}C NMR (75 MHz, $CDCl_3$)

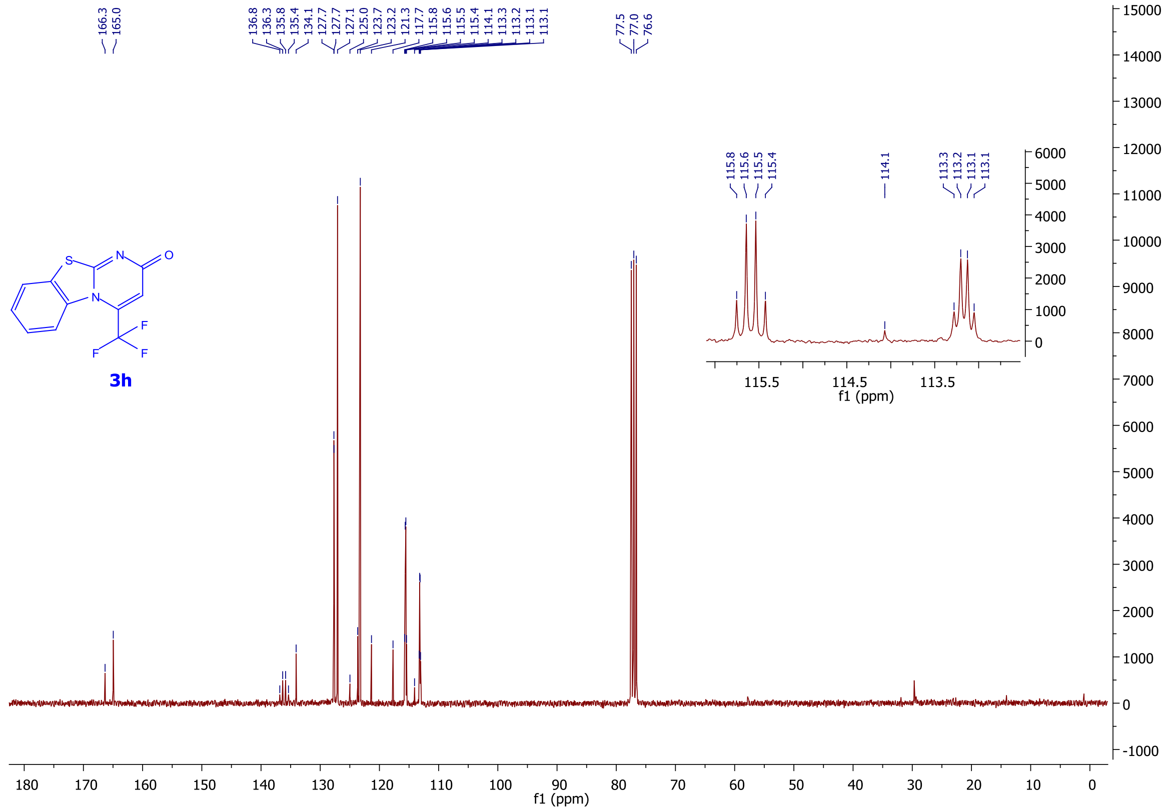

8-bromo-4-(trifluoromethyl)-2H-benzo[4,5]thiazolo[3,2-a]pyrimidin-2-one (**3i**).

^{1}H NMR (300 MHz, $CDCl_3$)

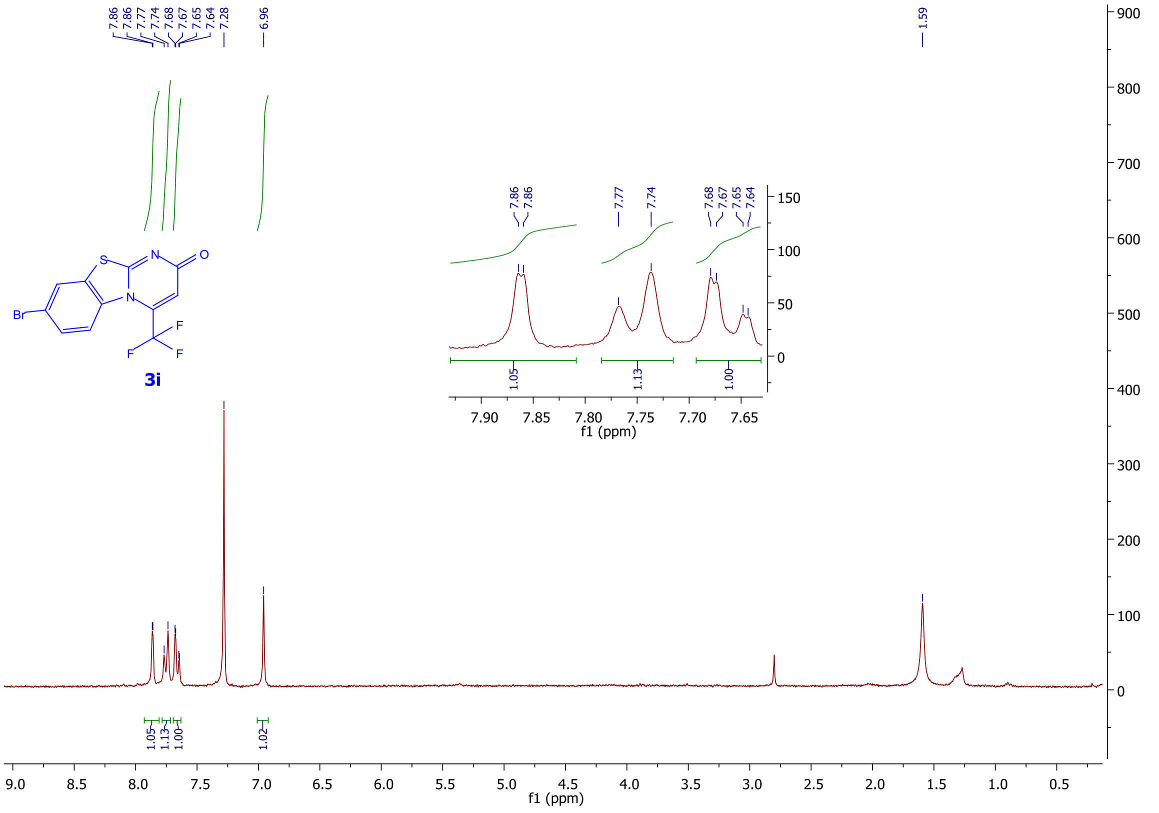

19F NMR (282 MHz, CDCl3)

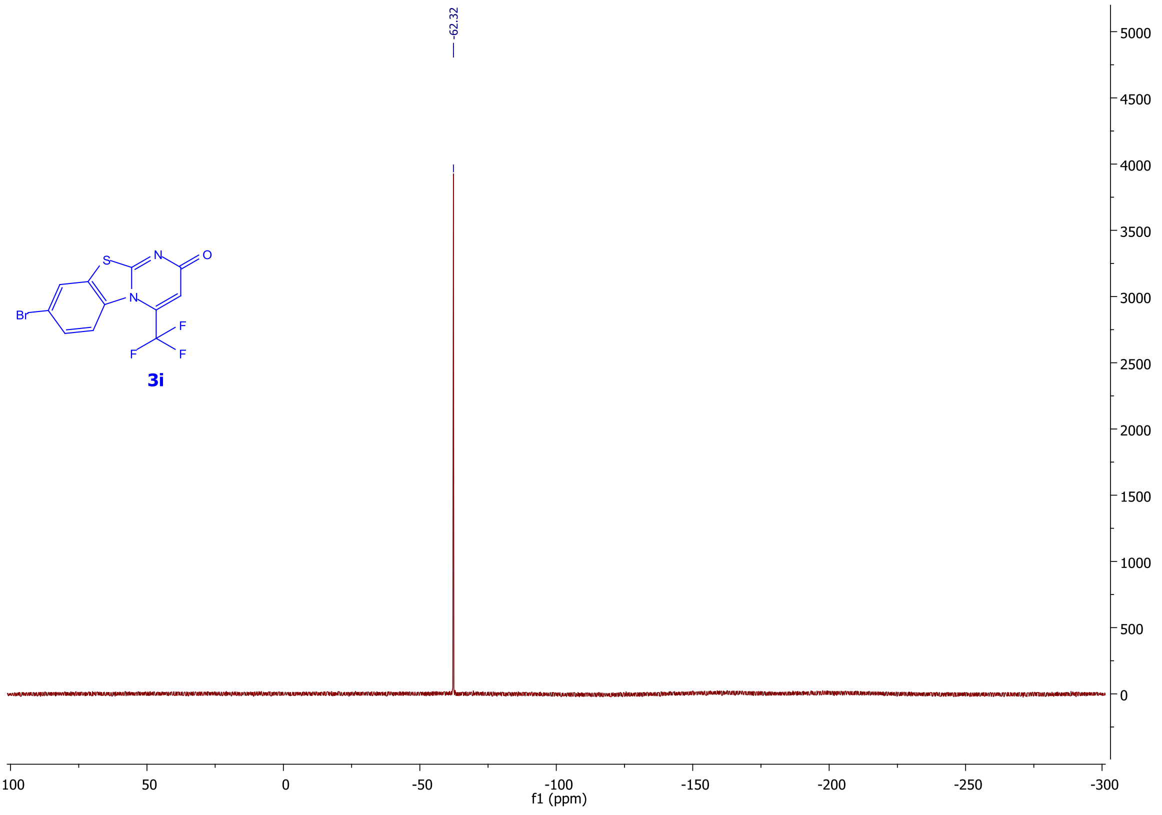

13C NMR (75 MHz, CDCl3)

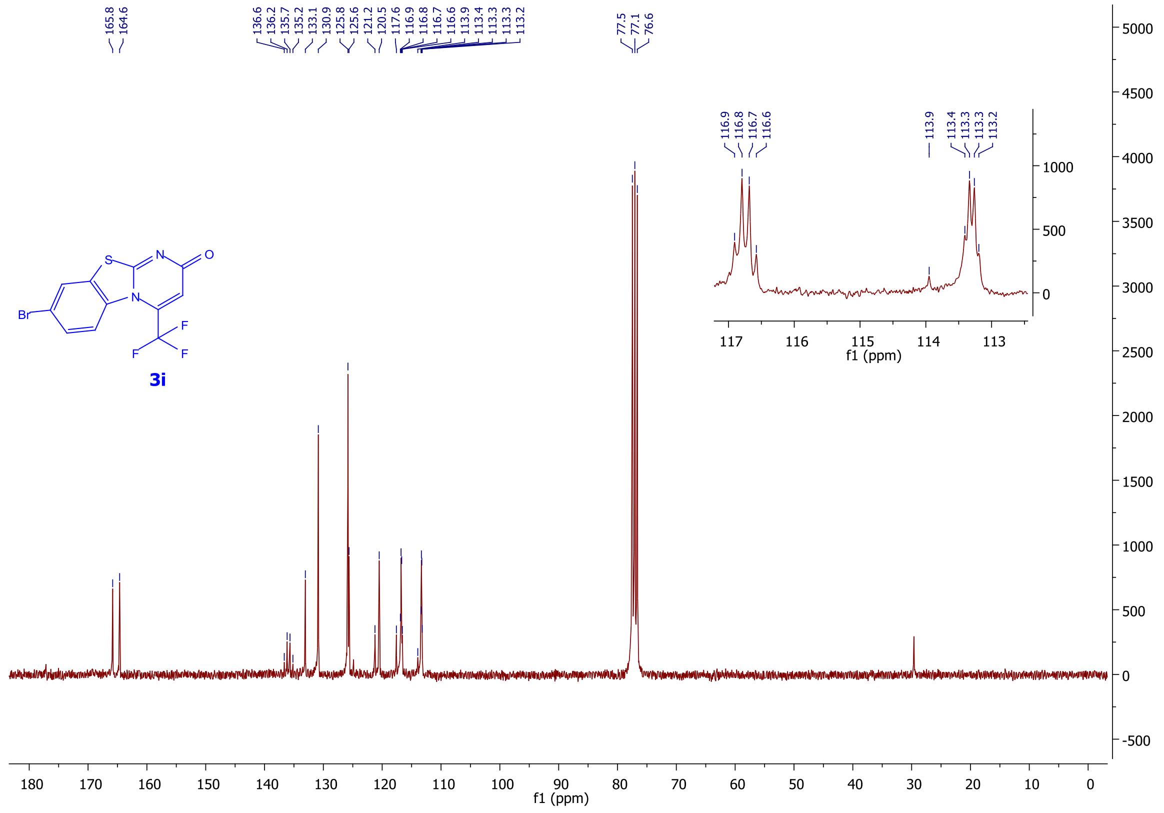

5-(trifluoromethyl)-7H-oxazolo[3,2-a]pyrimidin-7-one (**3j**)

^{1}H NMR (300 MHz, $CDCl_3$)

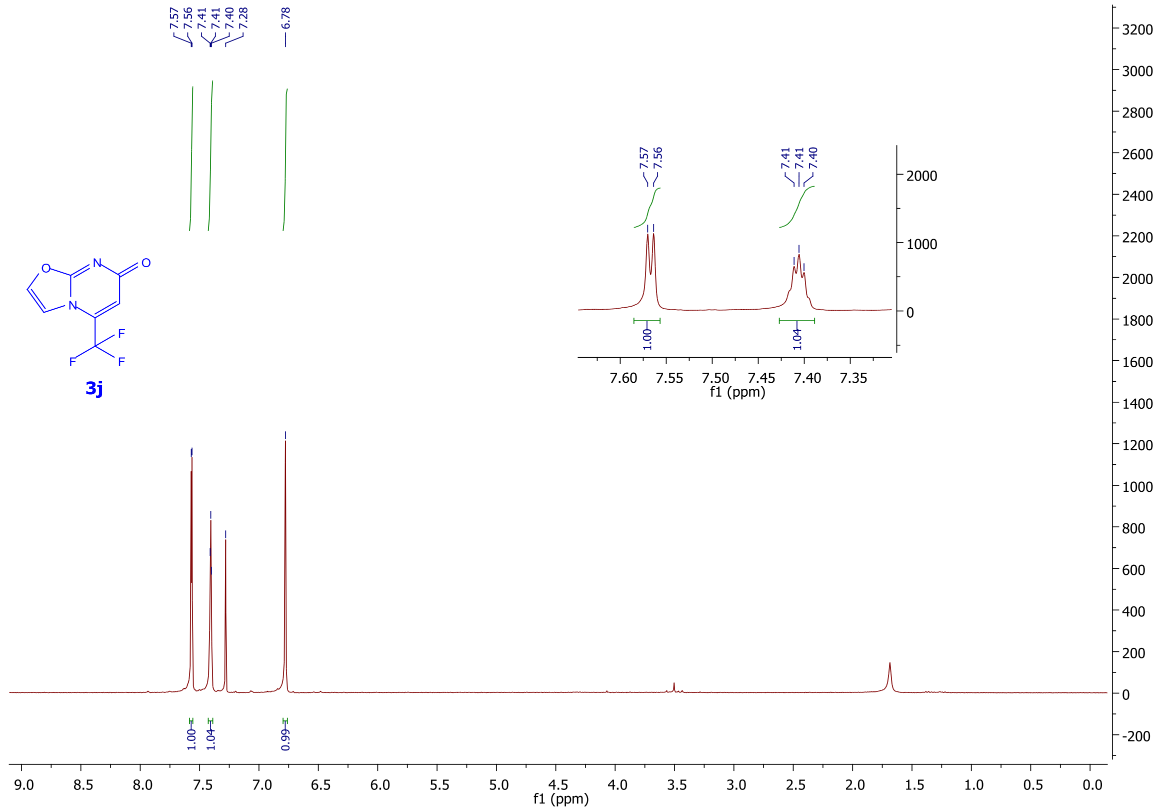

^{19}F NMR (282 MHz, $CDCl_3$)

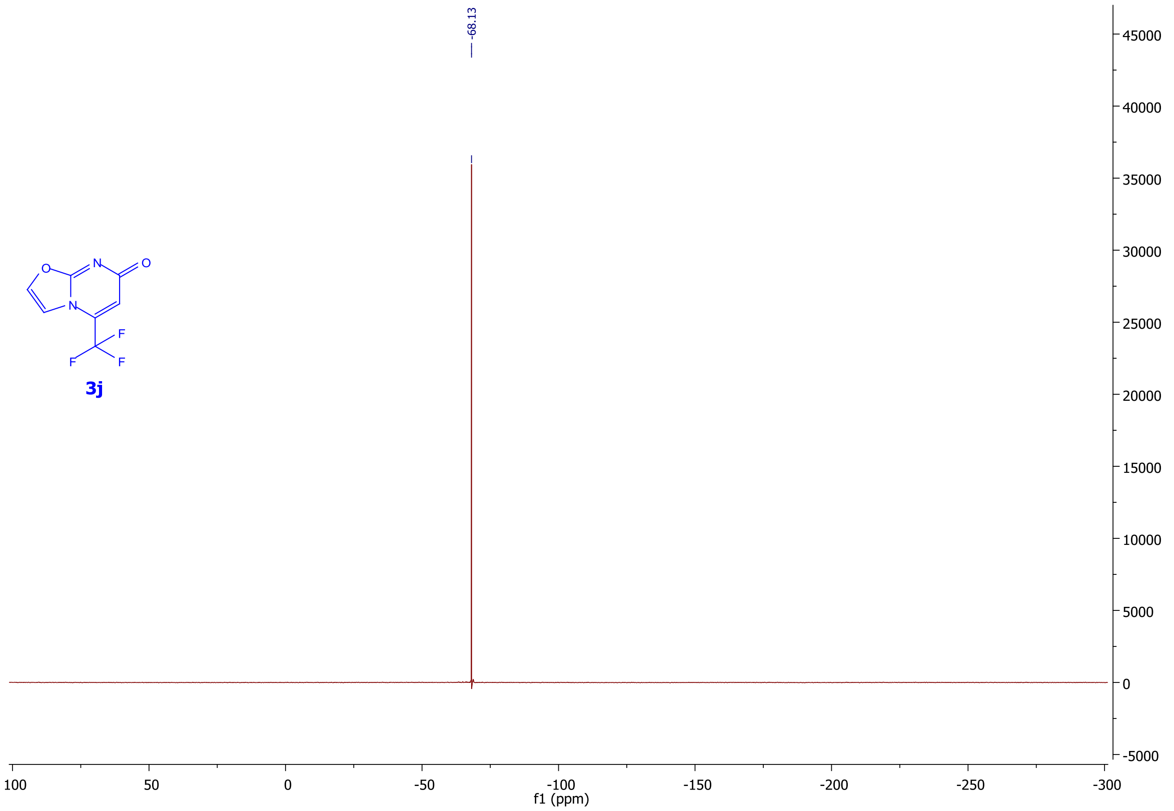

^{13}C NMR (75 MHz, DMSO-d_6)

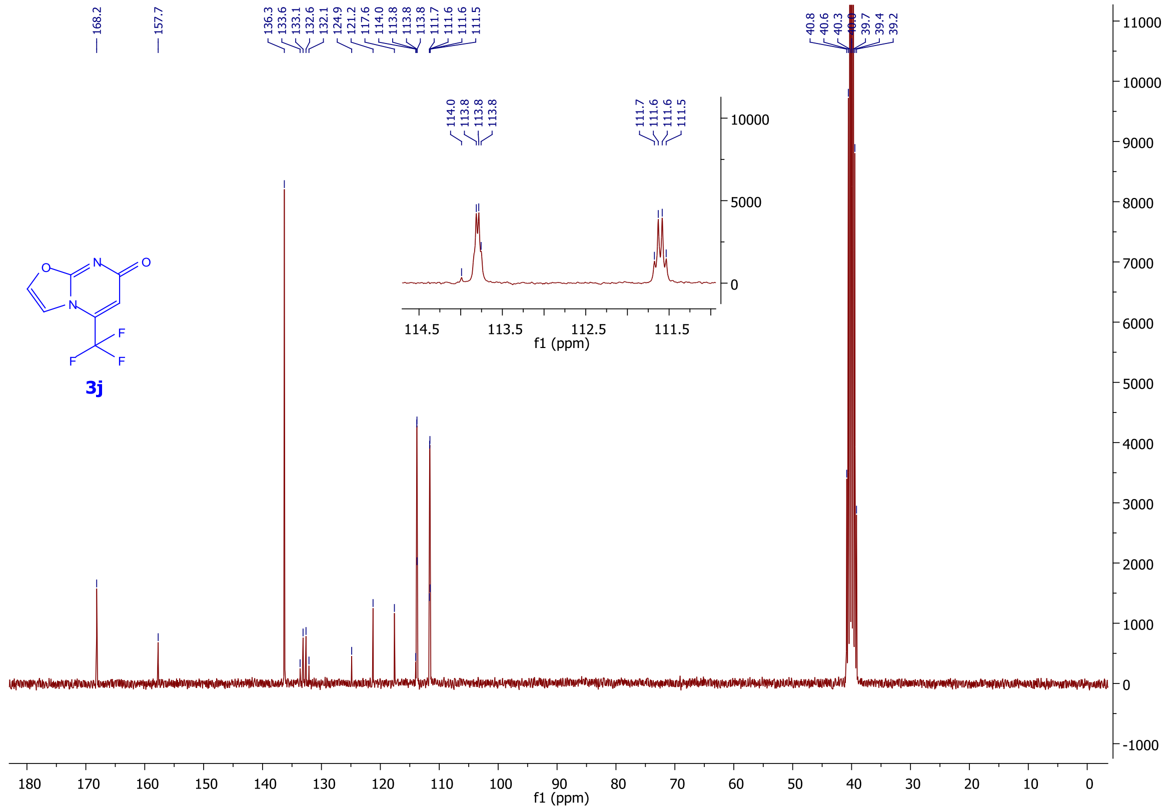

5-(perfluoroethyl)-7H-oxazolo[3,2-a]pyrimidin-7-one (**3k**)

^{1}H NMR (300 MHz, $CDCl_3$)

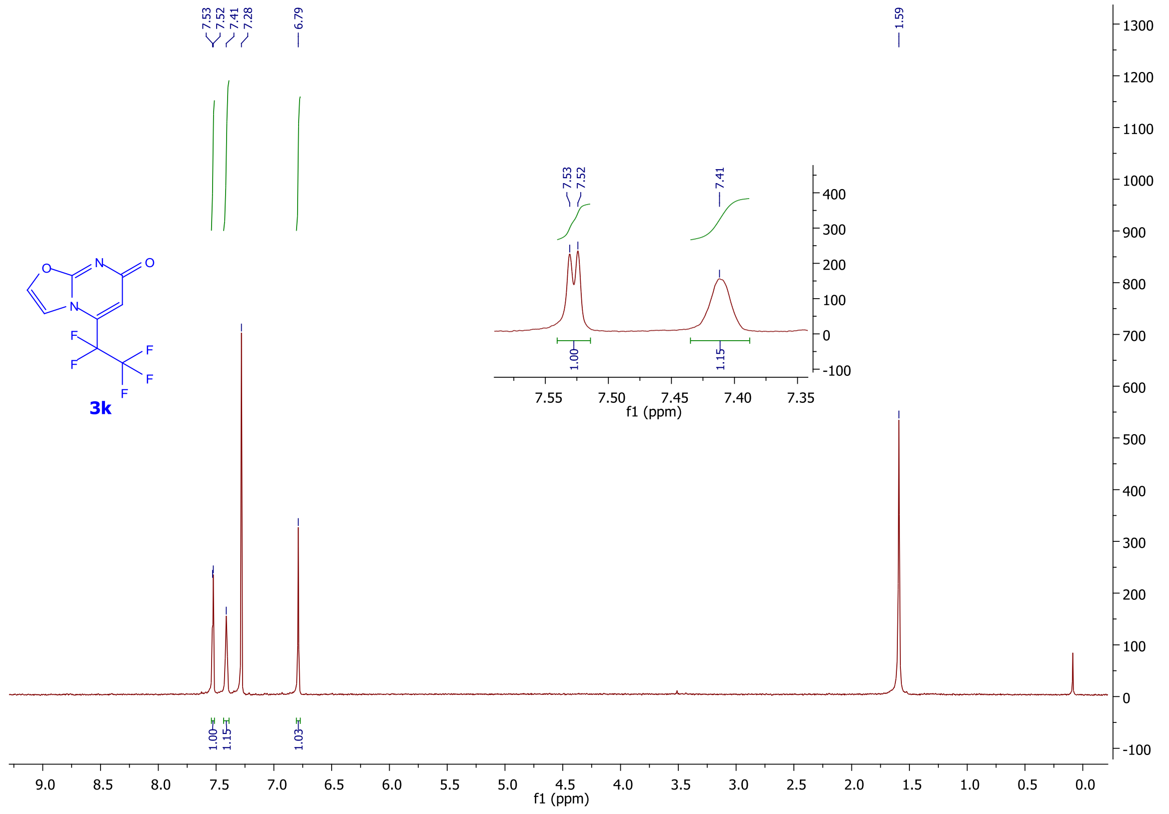

^{19}F NMR (282 MHz, $CDCl_3$)

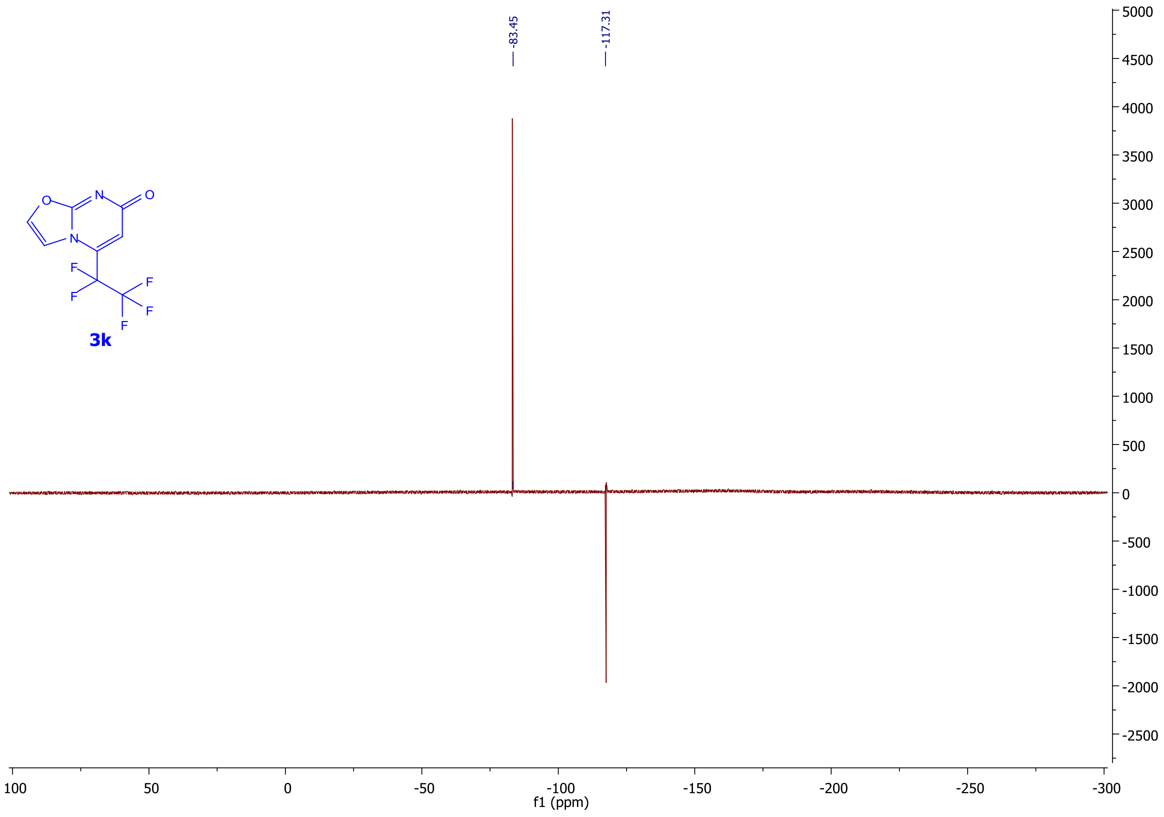

^{13}C NMR (75 MHz, $CDCl_3$)

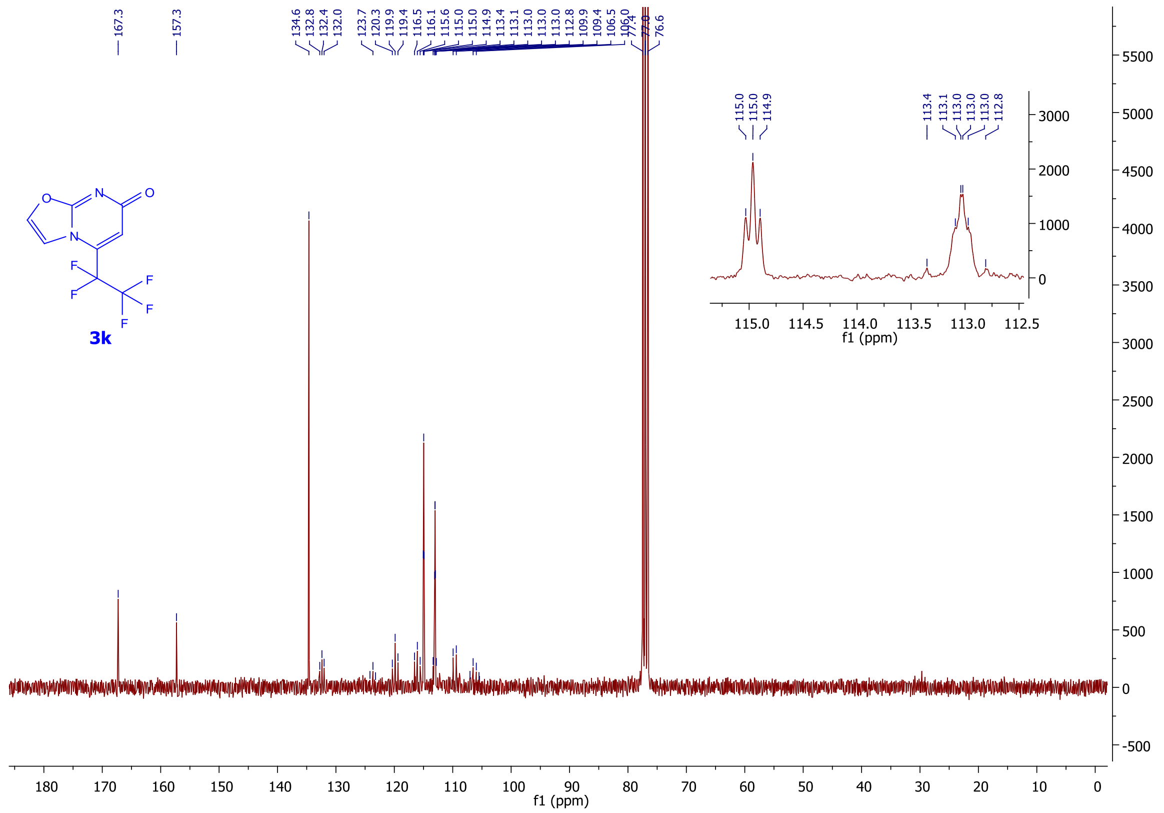

5-((4-bromophenyl)difluoromethyl)-7H-oxazolo[3,2-a]pyrimidin-7-one (**3l**)

^{1}H NMR (300 MHz, $CDCl_3$)

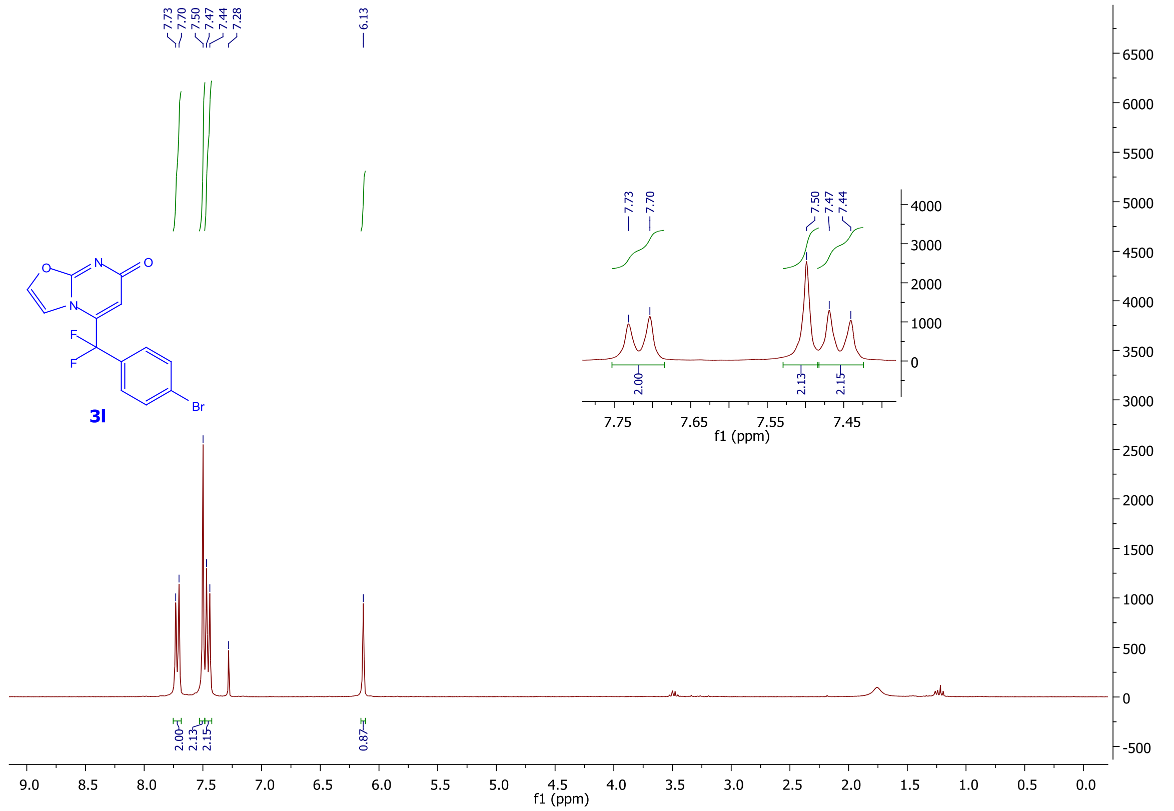

^{19}F NMR (282 MHz, $CDCl_3$)

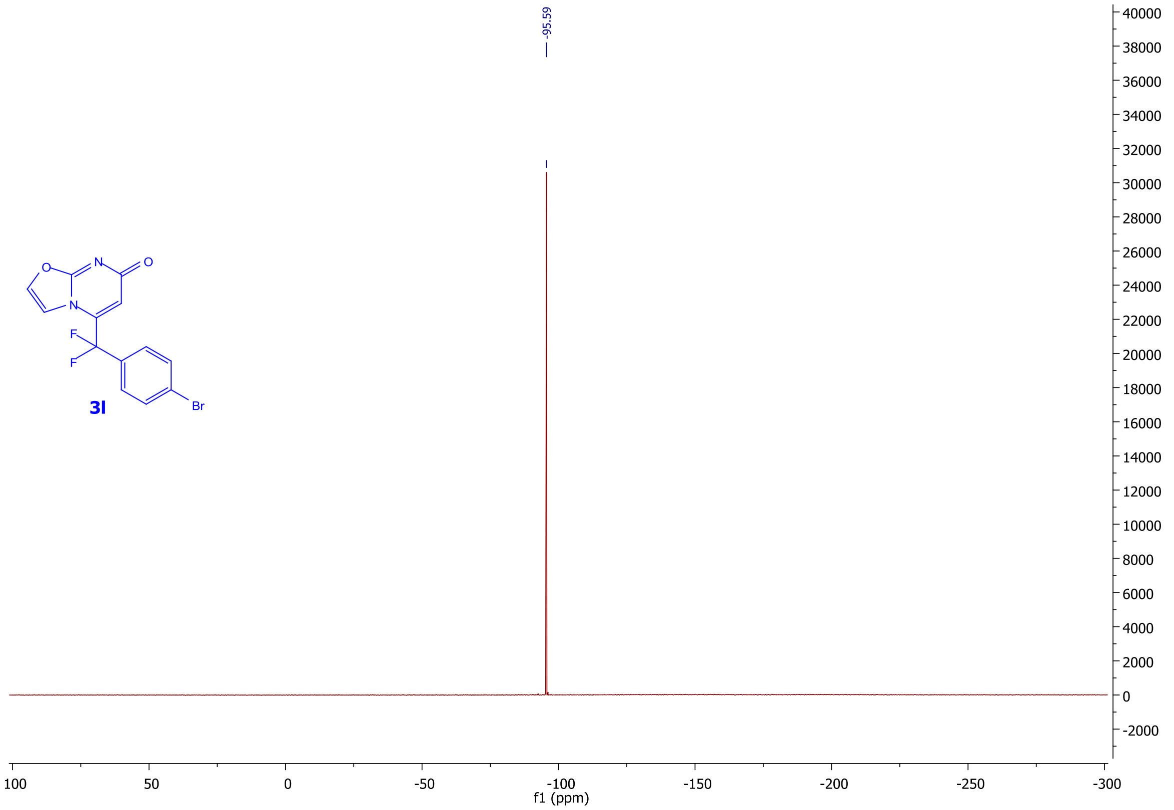

^{13}C NMR (75 MHz, $CDCl_3$)

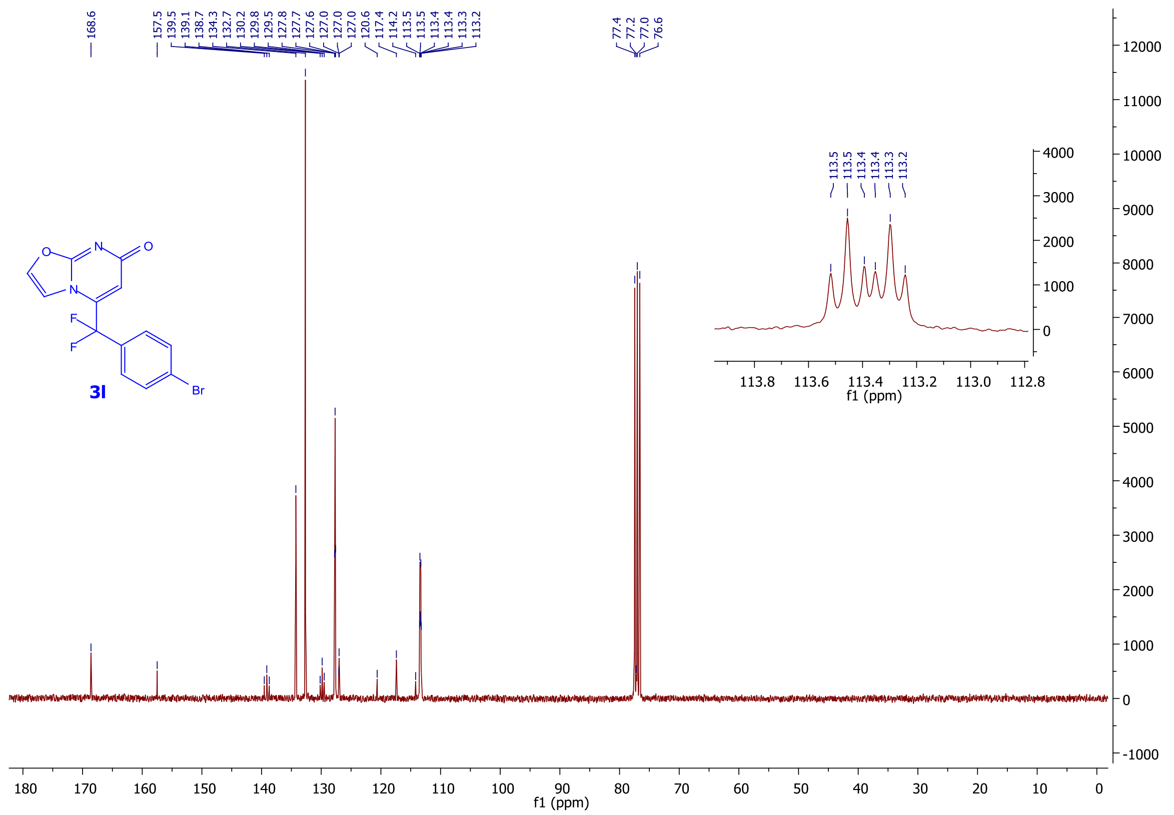

4-(trifluoromethyl)-2H-benzo[4,5]oxazolo[3,2-a]pyrimidin-2-one (**3m**)

^{1}H NMR (300 MHz, DMSO-d_6)

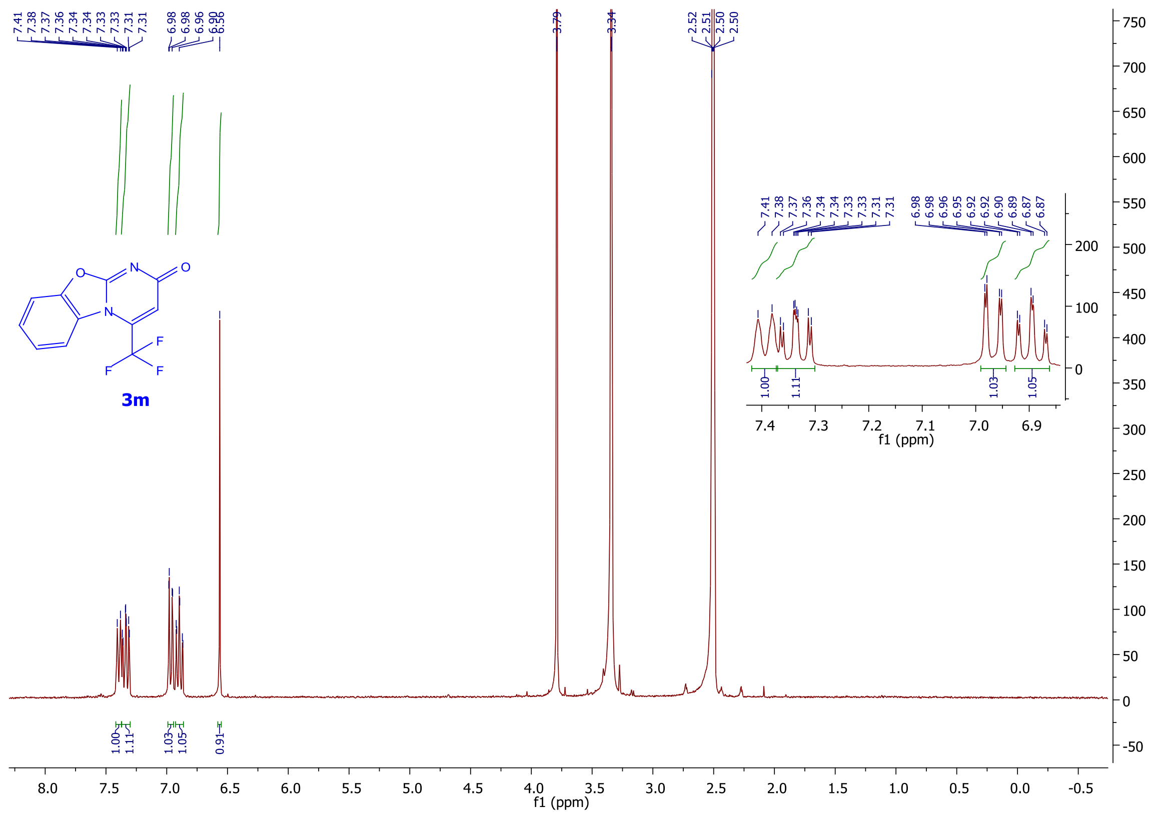

^{19}F NMR (282 MHz, DMSO-d_6)

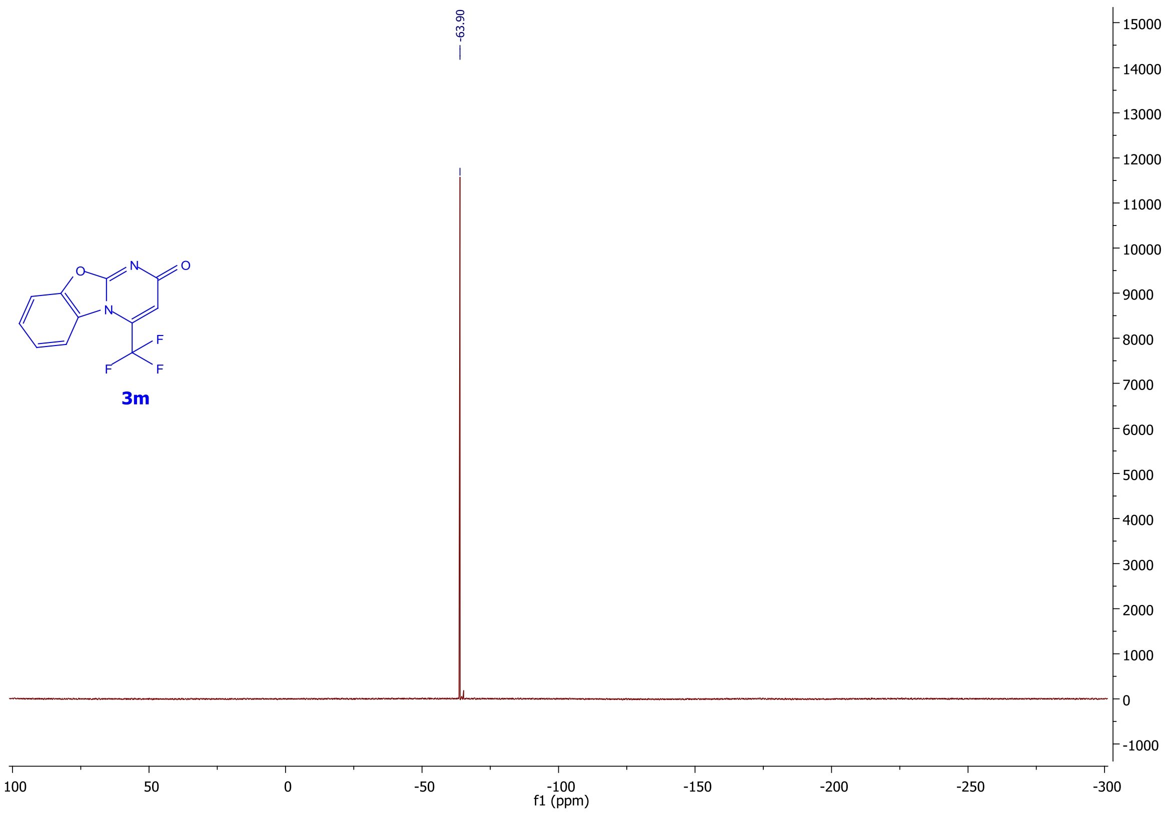

^{13}C NMR (75 MHz, DMSO-d_6)

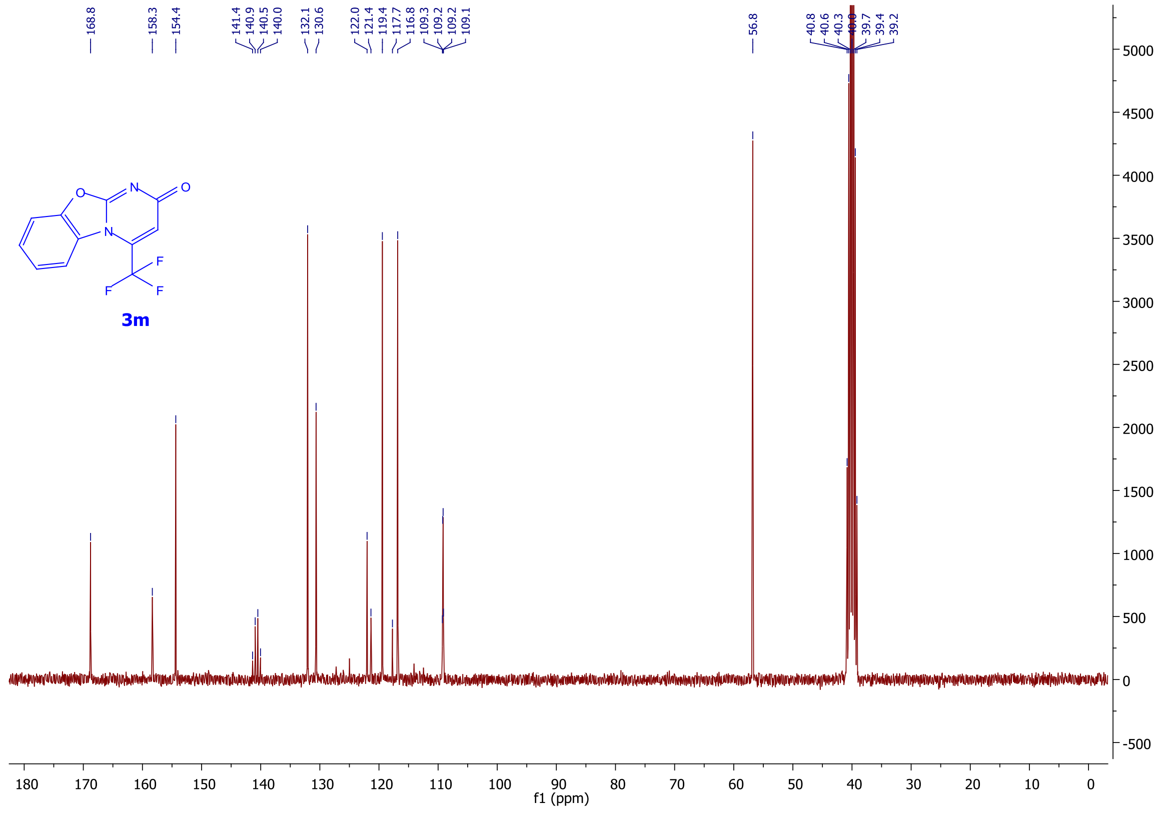

7-chloro-4-(trifluoromethyl)-2H-benzo[4,5]oxazolo[3,2-a]pyrimidin-2-one (**3n**)

^{1}H NMR (300 MHz, MeOH-*d4*)

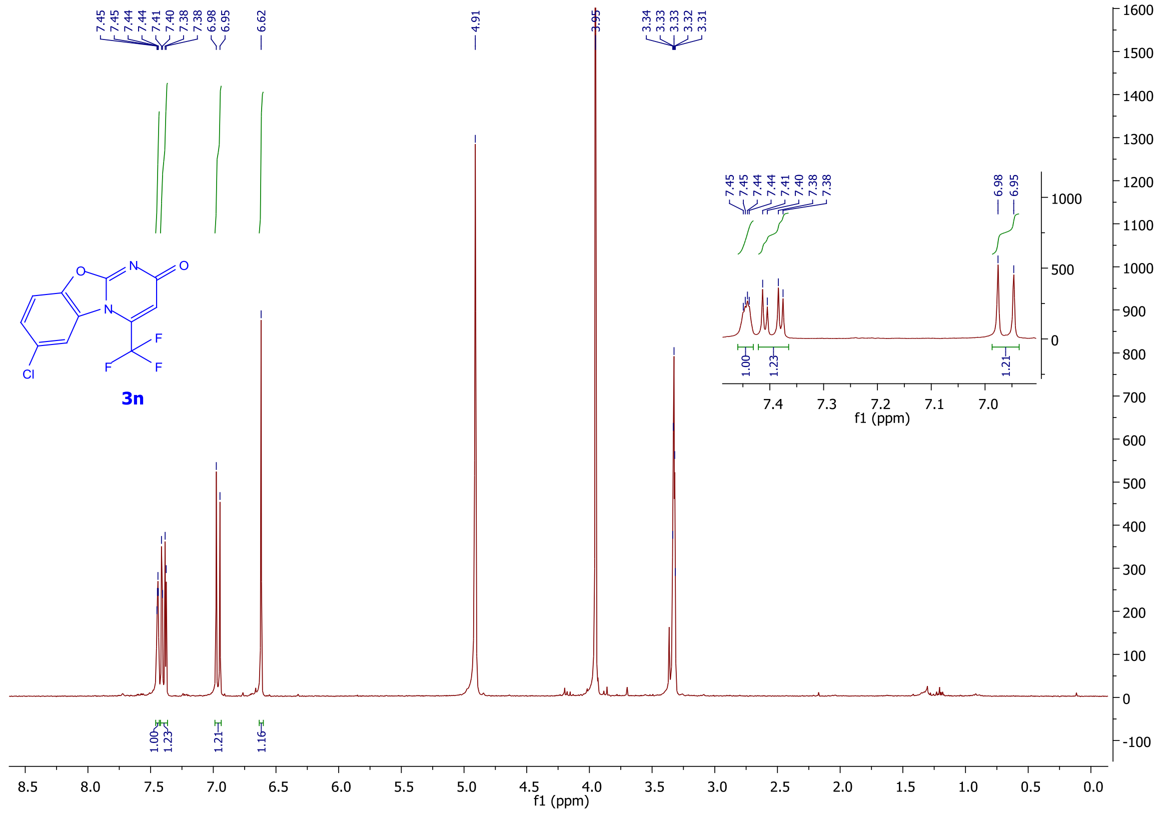

^{19}F NMR (282 MHz, MeOH-*d4*)

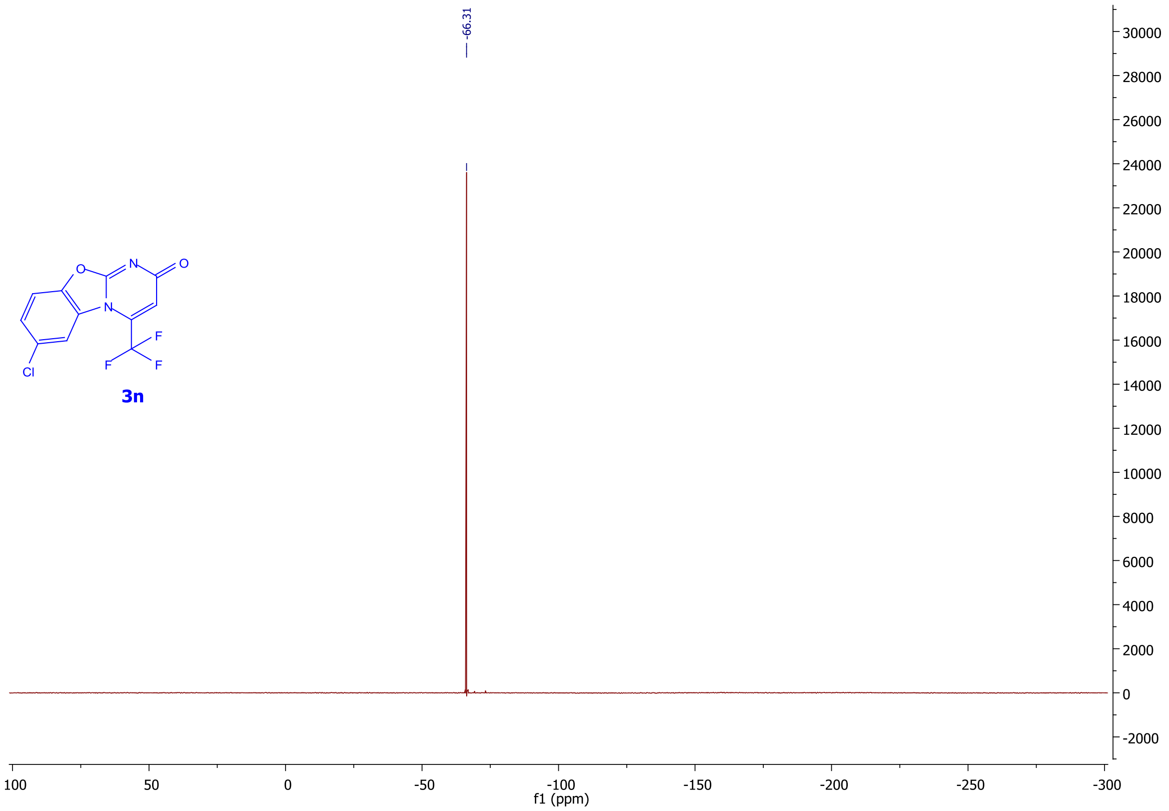

^{13}C NMR (75 MHz, MeOH-*d4*)

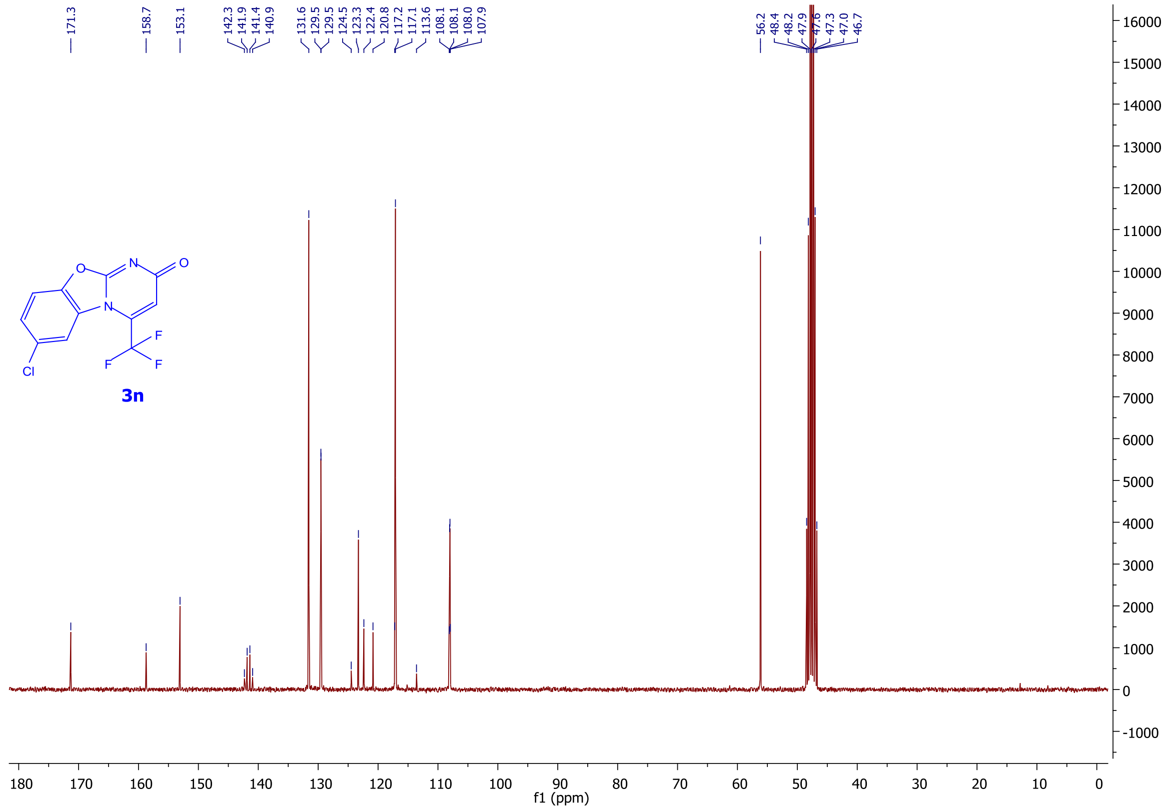

2-phenyl-5-(trifluoromethyl)-7*H*-thiazolo[3,2-*a*]pyrimidin-7-one (**4a**)

^{1}H NMR (300 MHz, $CDCl_3$)

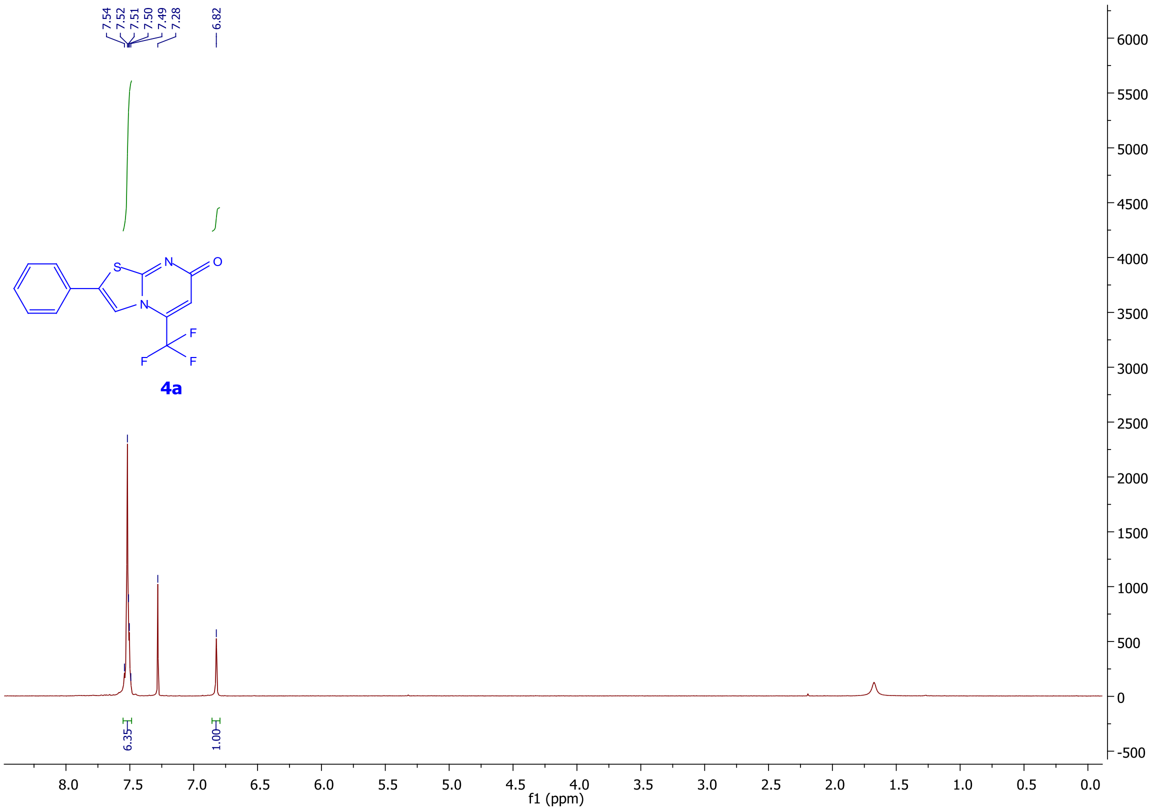

^{19}F NMR (282 MHz, $CDCl_3$)

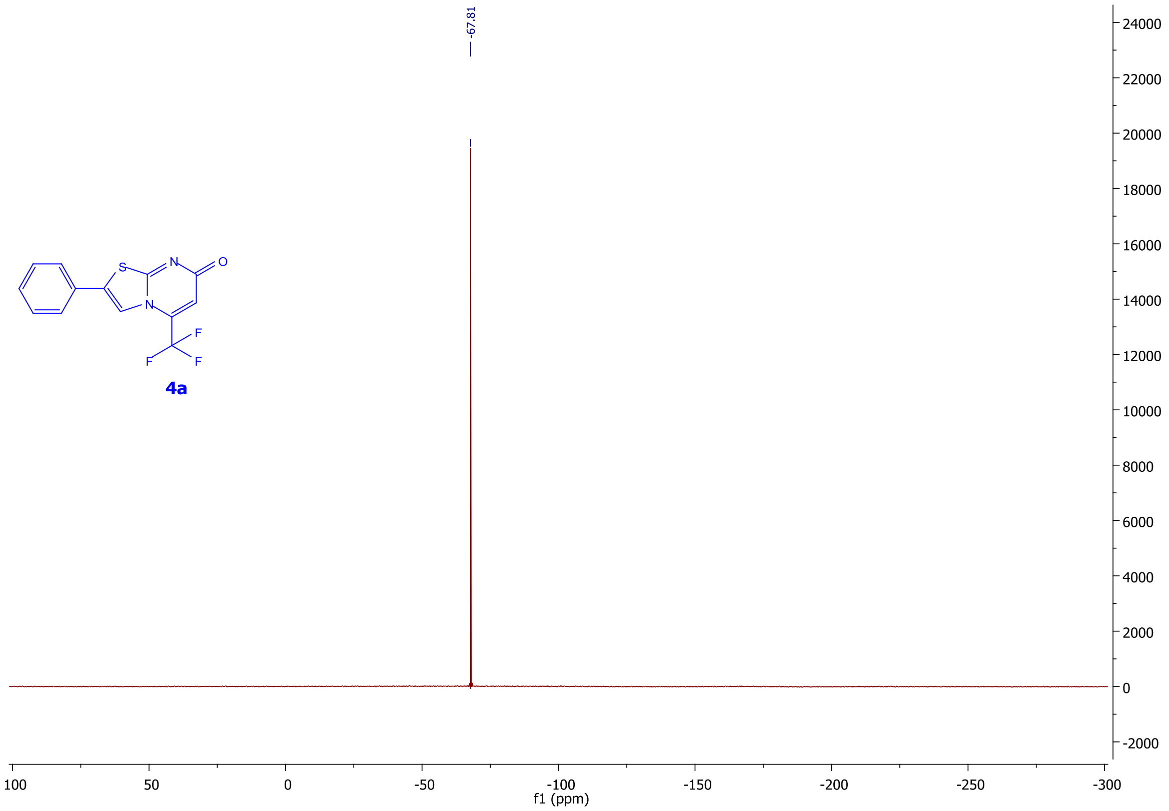

^{13}C NMR (75 MHz, $CDCl_3$)

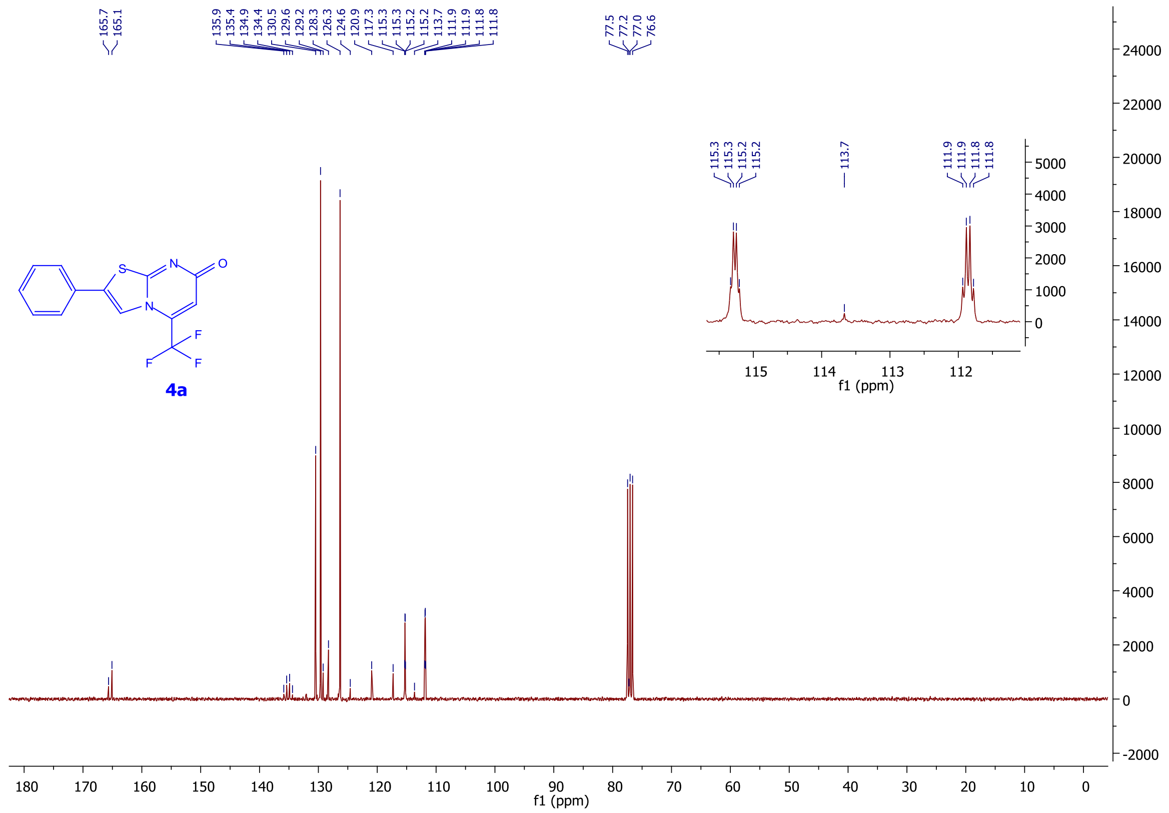

2-(2-methoxyphenyl)-5-(trifluoromethyl)-7*H*-thiazolo[3,2-*a*]pyrimidin-7-one (**4b**)

^{1}H NMR (300 MHz, $CDCl_3$)

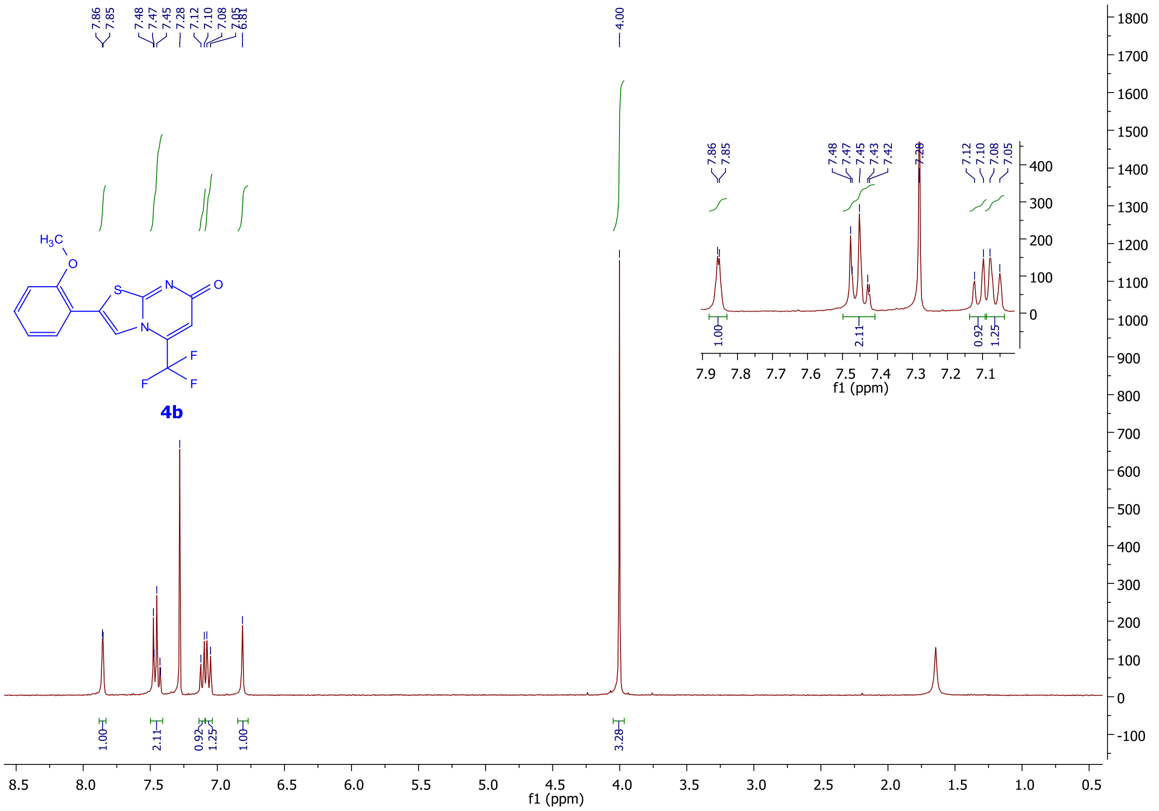

4b

^{19}F NMR (282 MHz, $CDCl_3$)

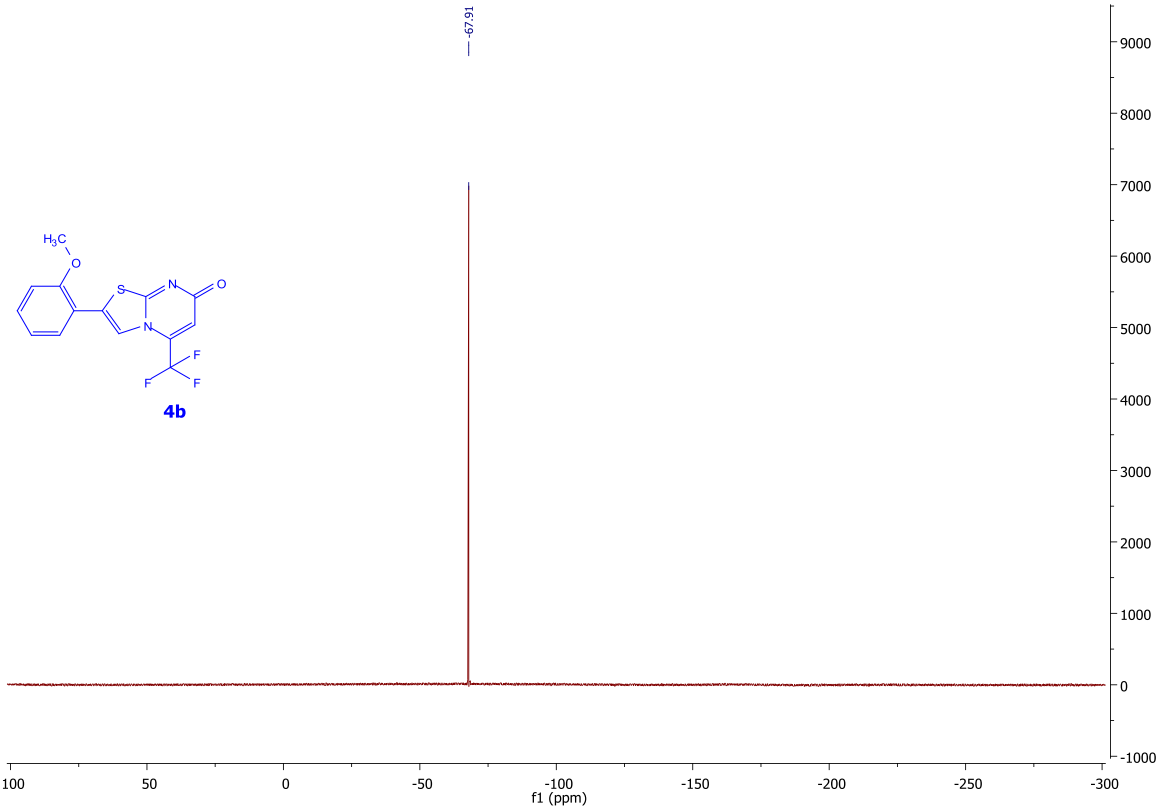

4b

^{13}C NMR (75 MHz, $CDCl_3$)

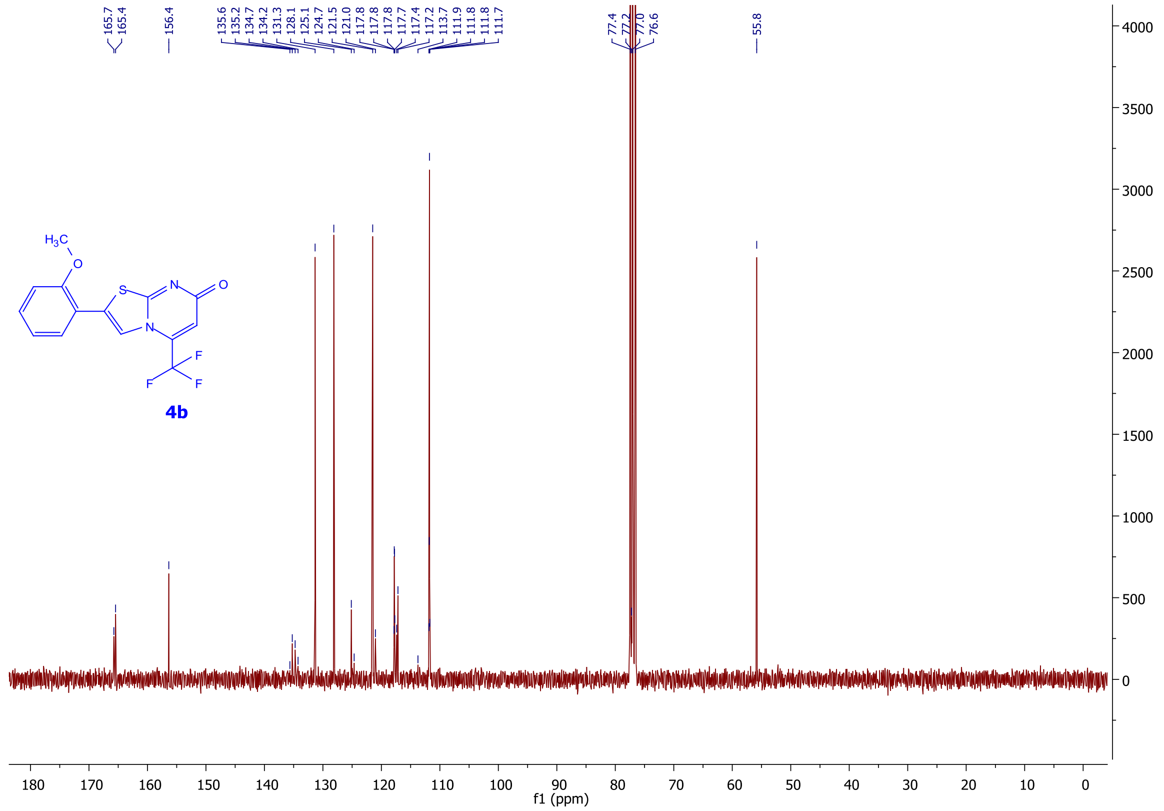

2-(3-methoxyphenyl)-5-(trifluoromethyl)-7*H*-thiazolo[3,2-*a*]pyrimidin-7-one (**4c**)

^{1}H NMR (300 MHz, $CDCl_3$)

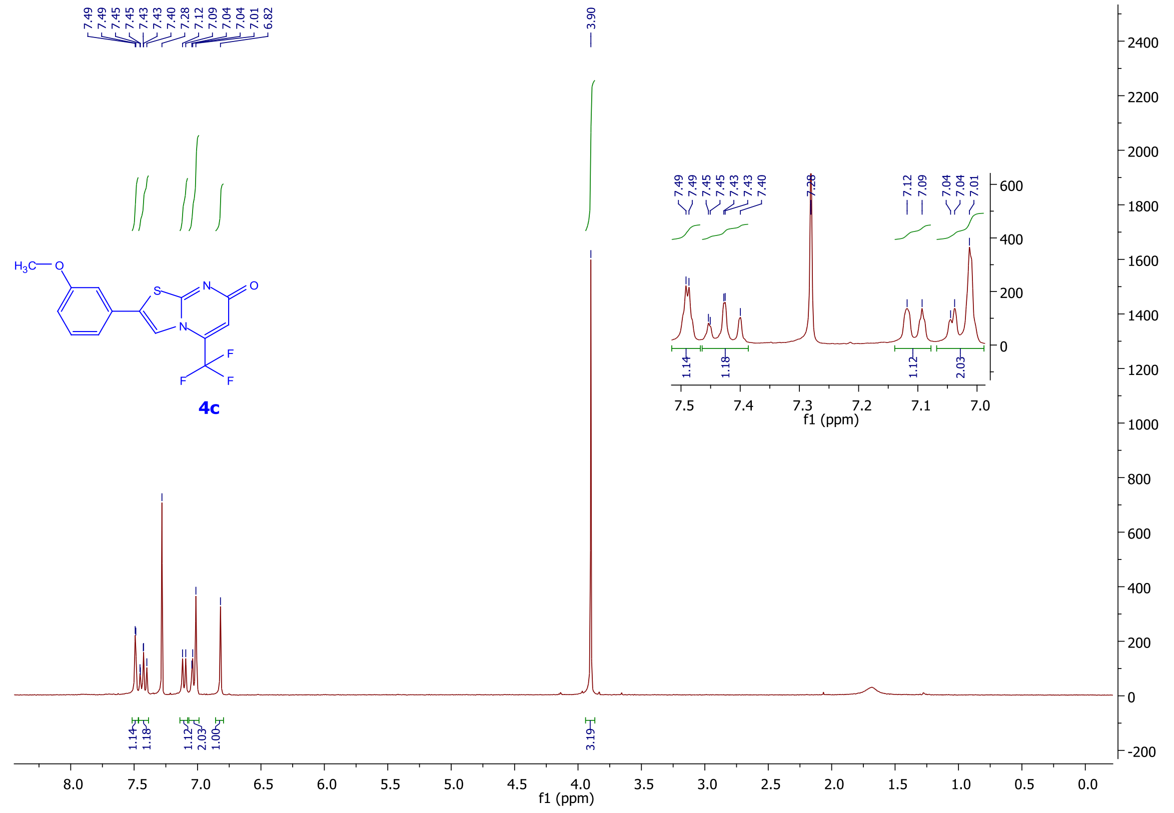

^{19}F NMR (282 MHz, $CDCl_3$)

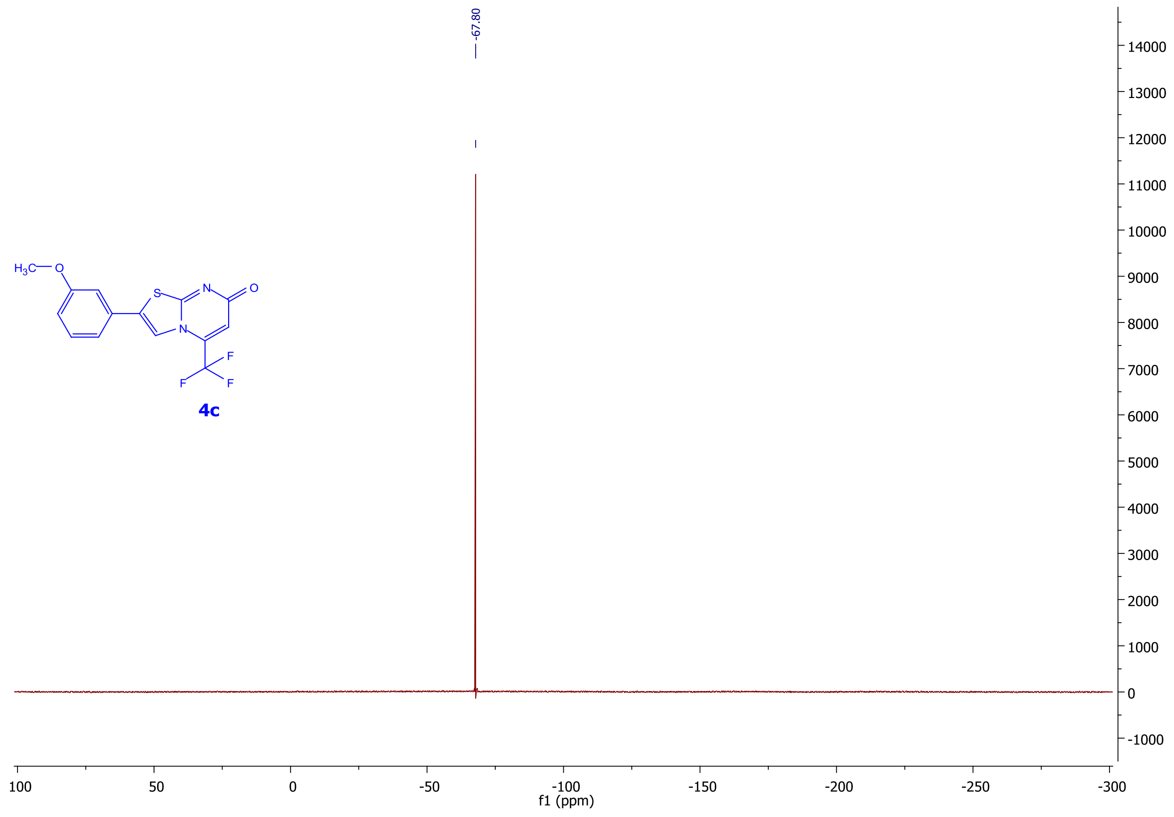

^{13}C NMR (75 MHz, $CDCl_3$)

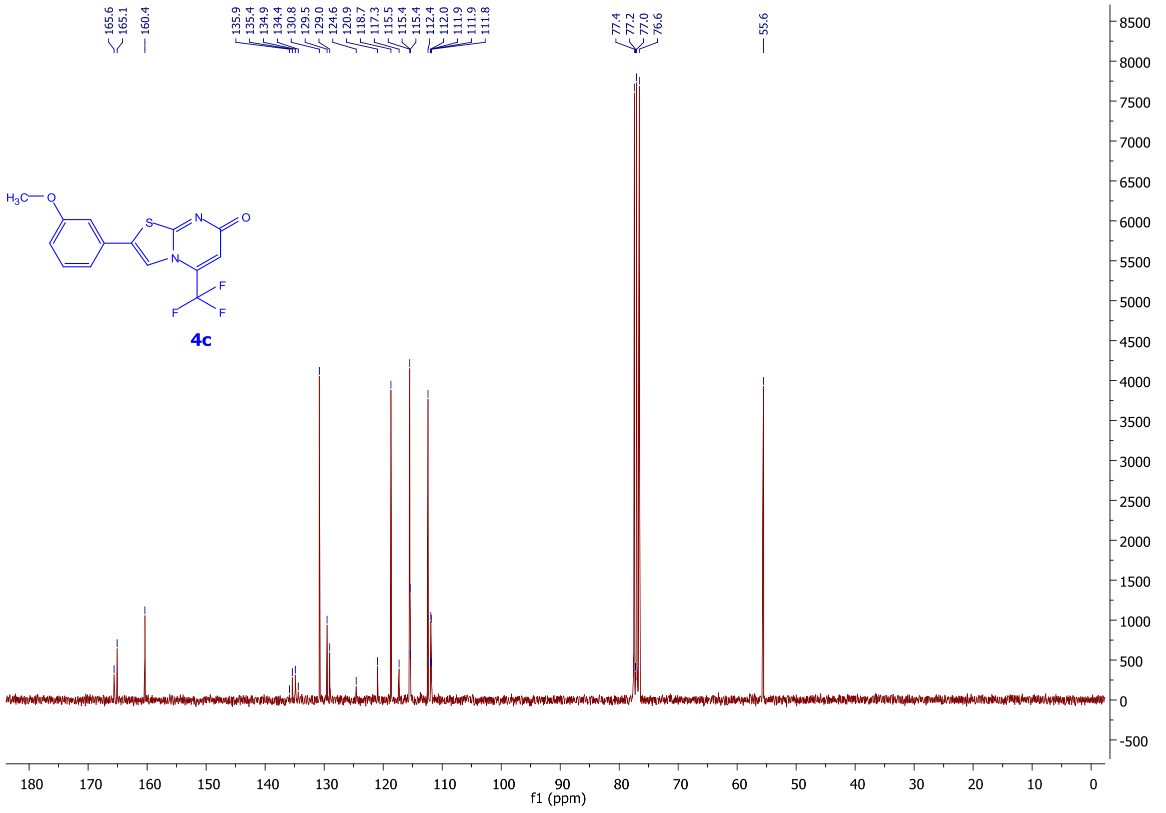

2-(4-methoxyphenyl)-5-(trifluoromethyl)-7*H*-thiazolo[3,2-*a*]pyrimidin-7-one (**4d**)

^{1}H NMR (300 MHz, $CDCl_3$)

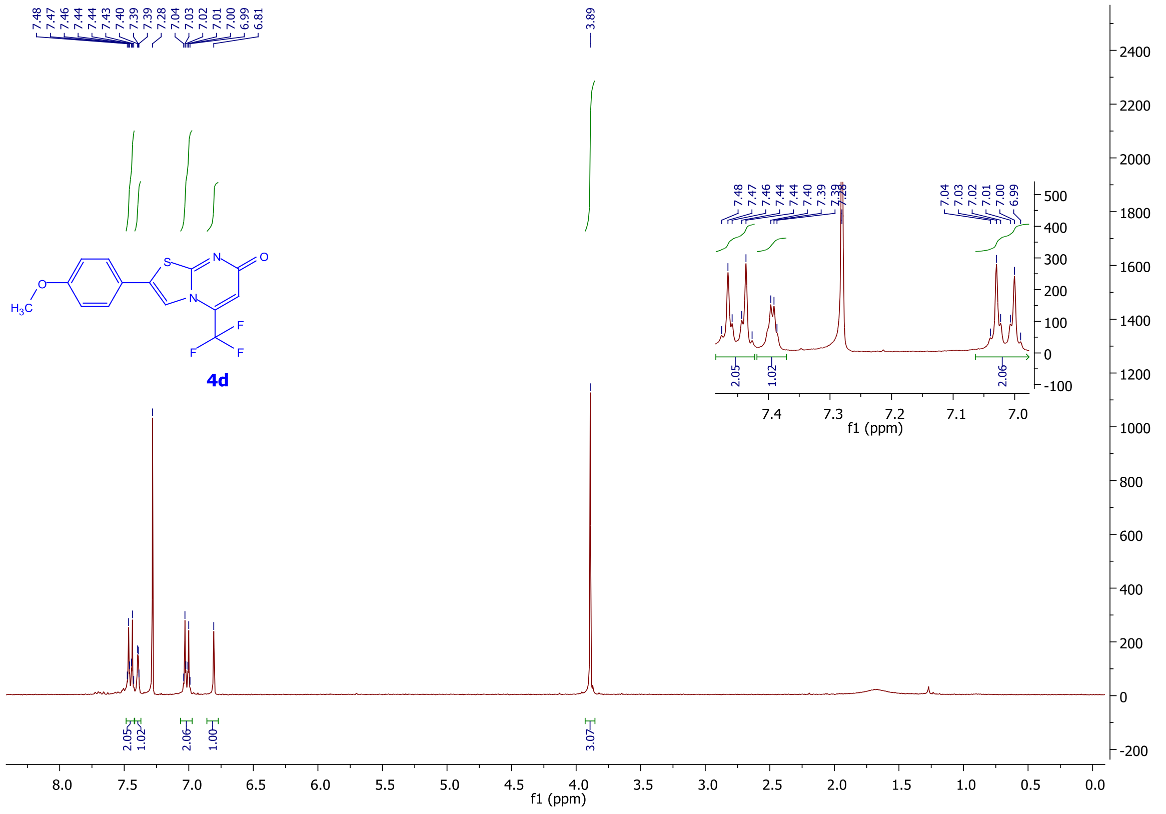

^{19}F NMR (282 MHz, $CDCl_3$)

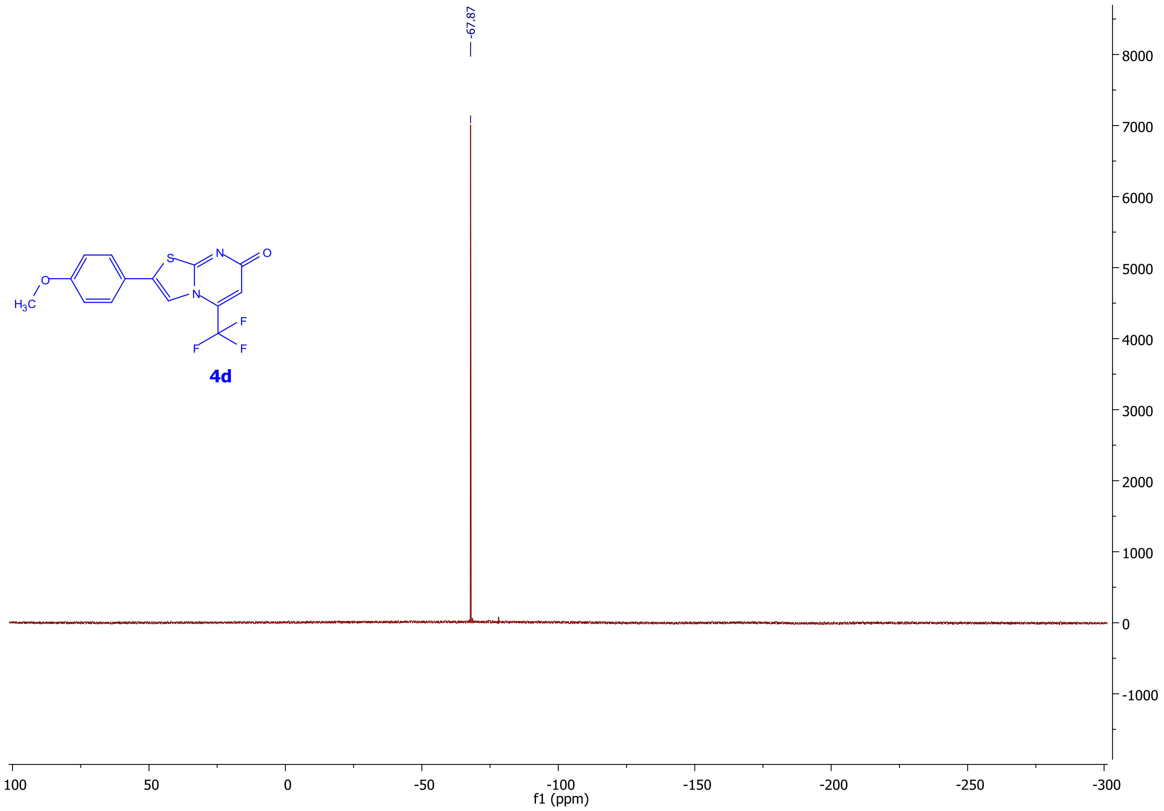

^{13}C NMR (75 MHz, $CDCl_3$)

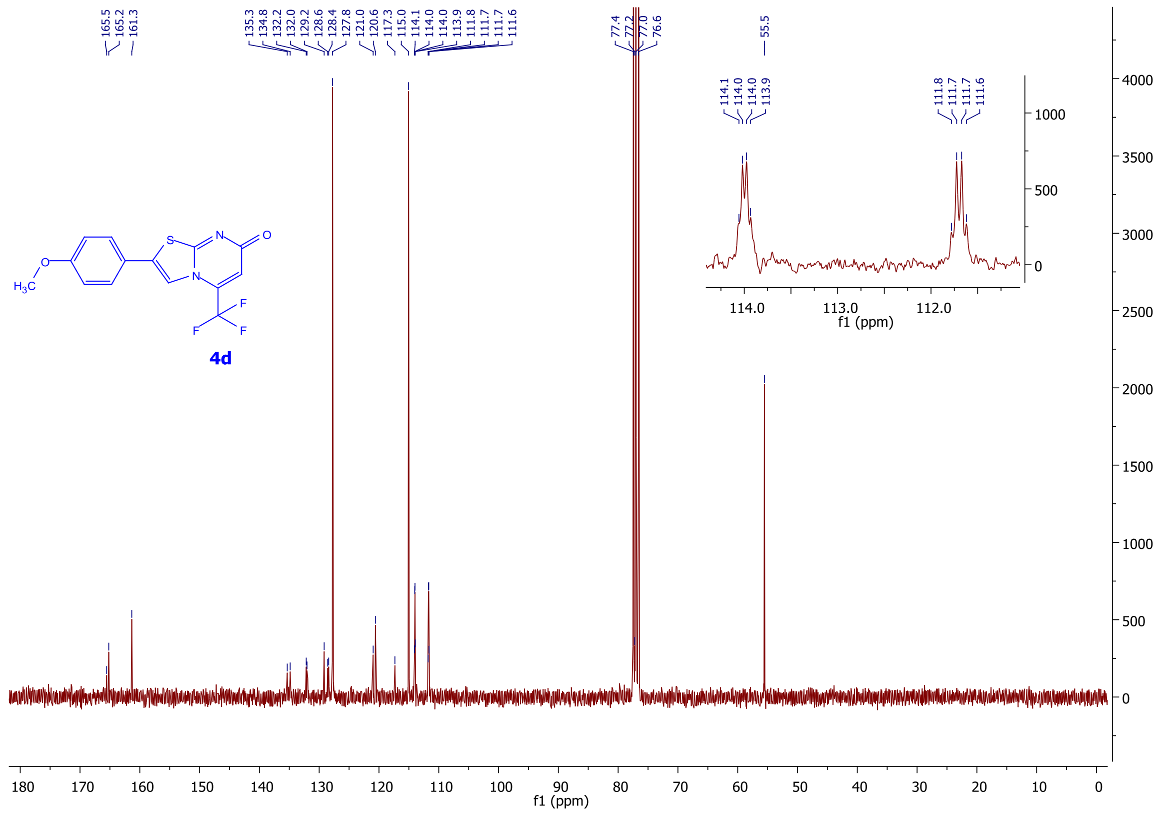

2-(4-methoxyphenyl)-5-(perfluoroethyl)-7*H*-thiazolo[3,2-*a*]pyrimidin-7-one (**4e**)

^{1}H NMR (300 MHz, $CDCl_3$)

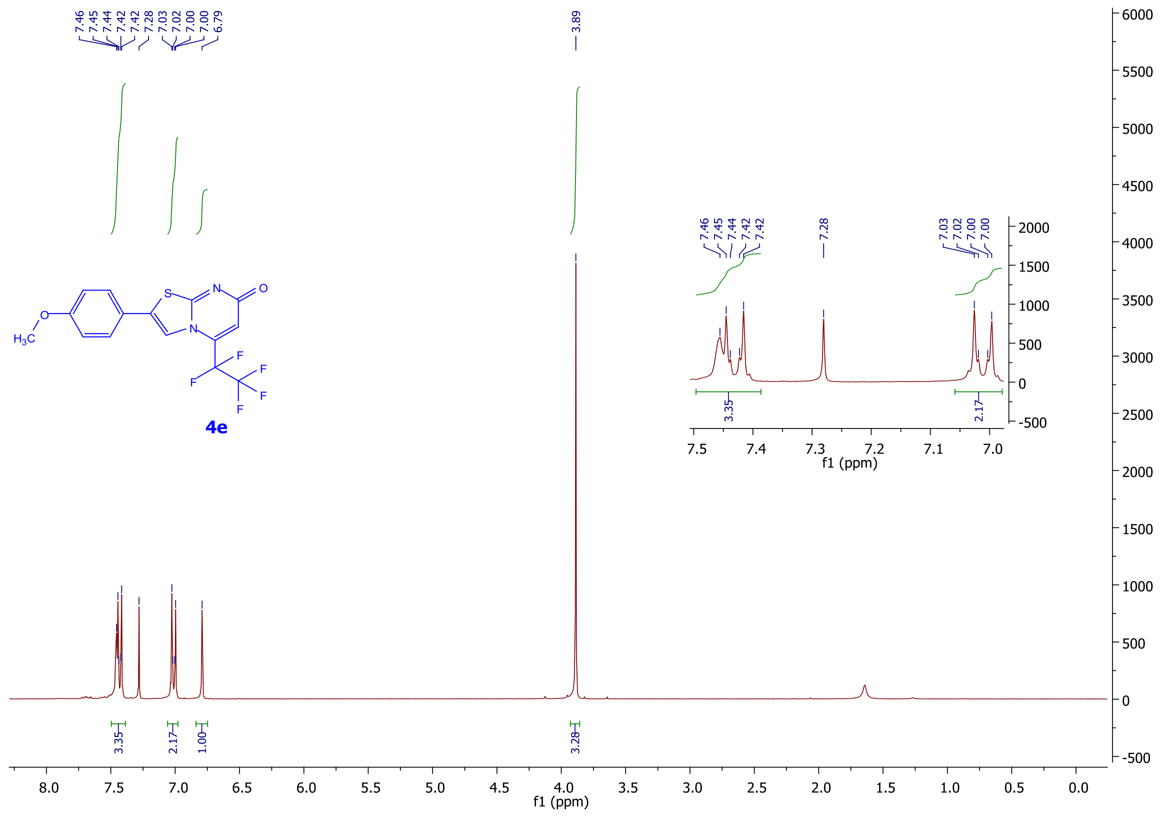

^{19}F NMR (282 MHz, $CDCl_3$)

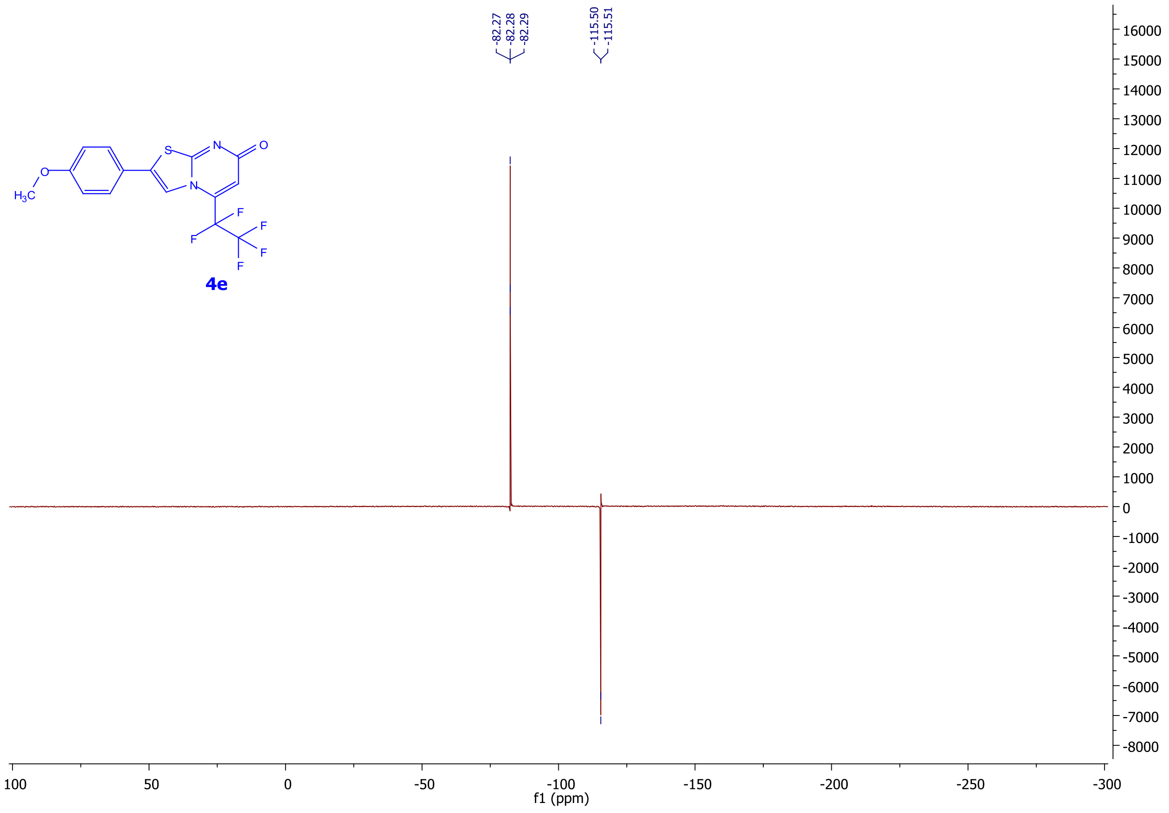

^{13}C NMR (75 MHz, $CDCl_3$)

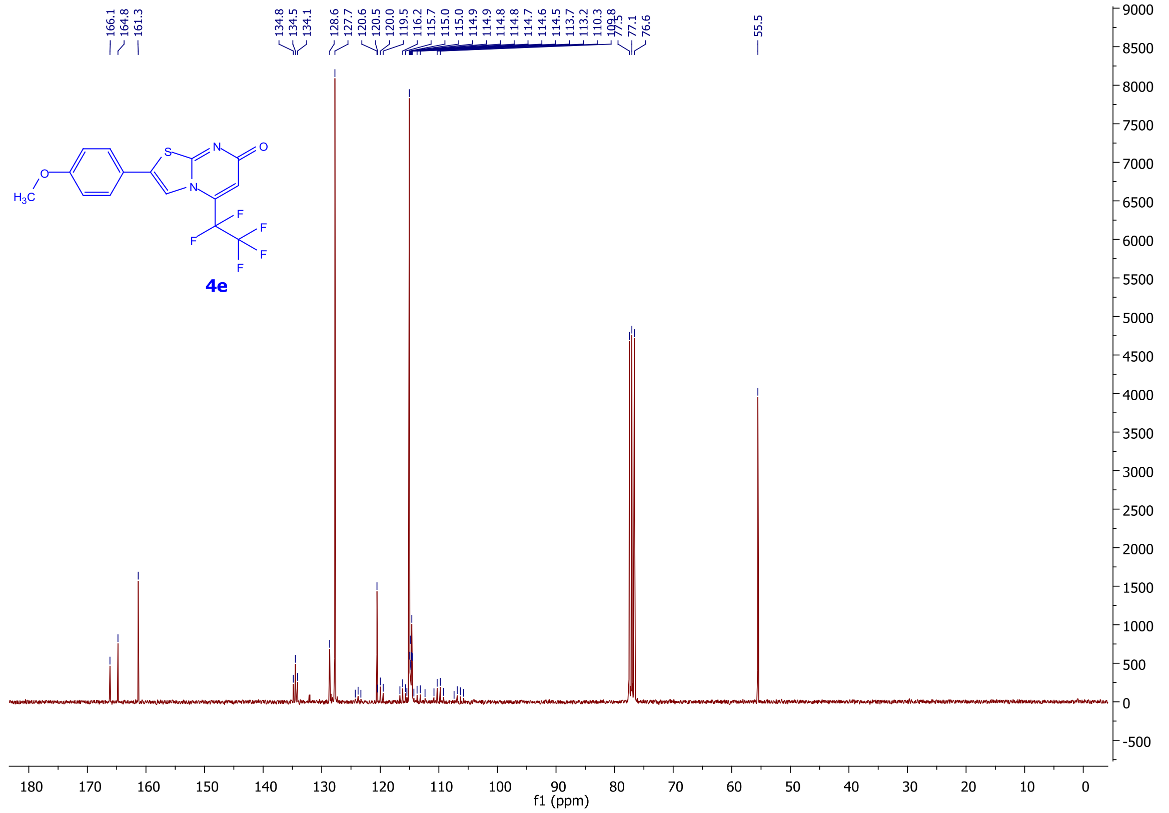

2-(2,4-dimethoxyphenyl)-5-(trifluoromethyl)-7*H*-thiazolo[3,2-*a*]pyrimidin-7-one (**4f**)

^{1}H NMR (300 MHz, $CDCl_3$)

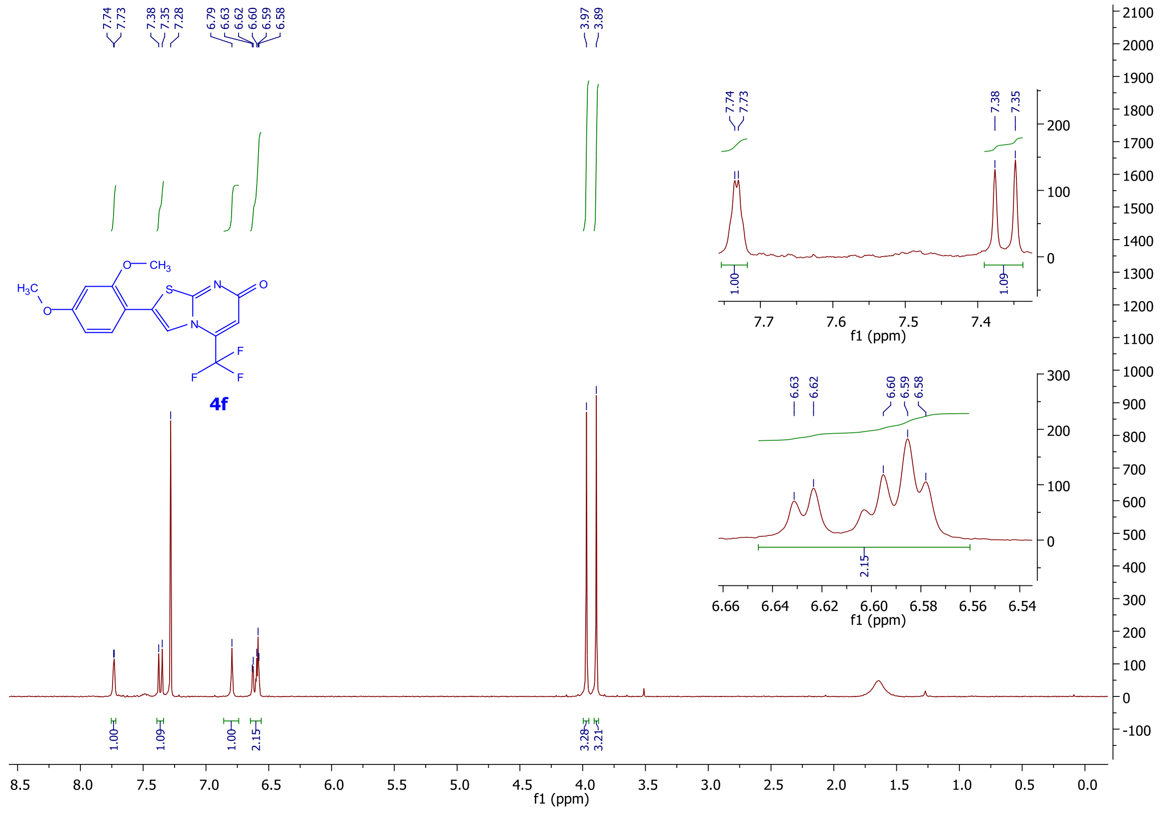

^{19}F NMR (282 MHz, $CDCl_3$)

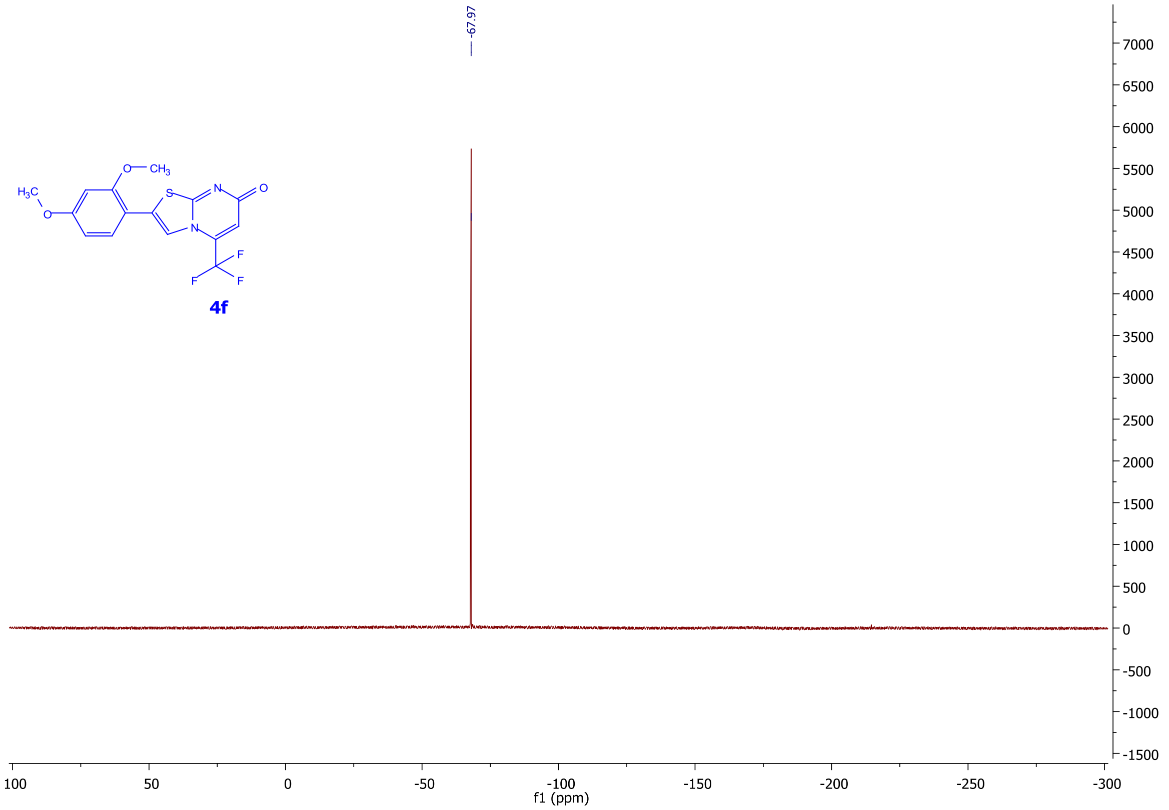

^{13}C NMR (75 MHz, $CDCl_3$)

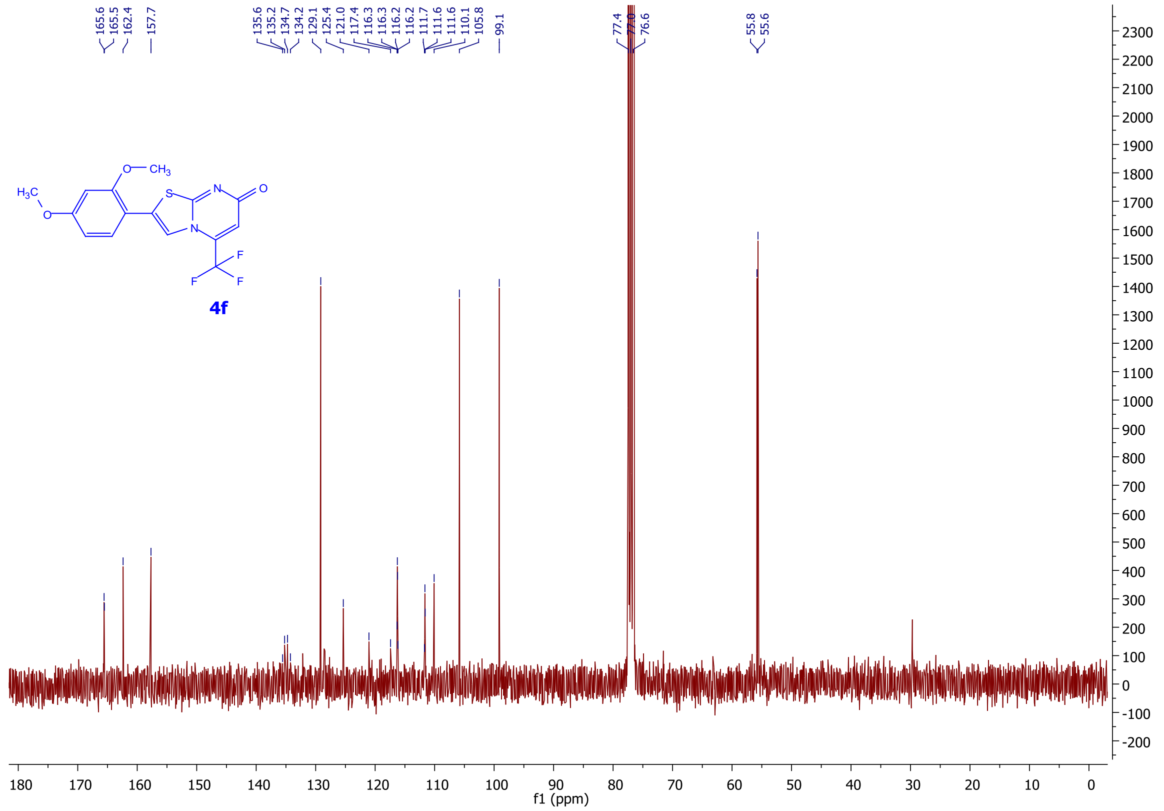

2-(2,3-dihydrobenzo[*b*][1,4]dioxin-6-yl)-5-(trifluoromethyl)-7*H*-thiazolo[3,2-*a*]pyrimidin-7-one (**4g**)

^{1}H NMR (300 MHz, MeOH-d_4)

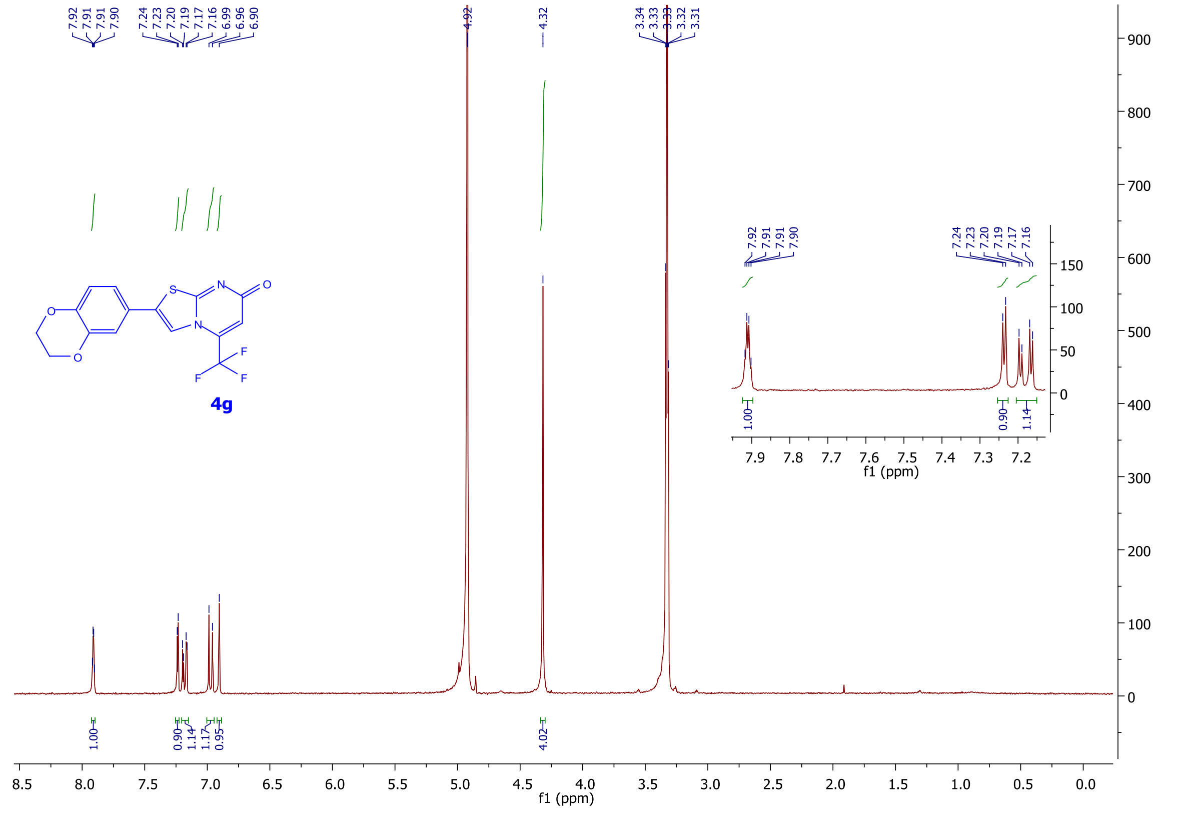

^{19}F NMR (282 MHz, MeOH-d_4)

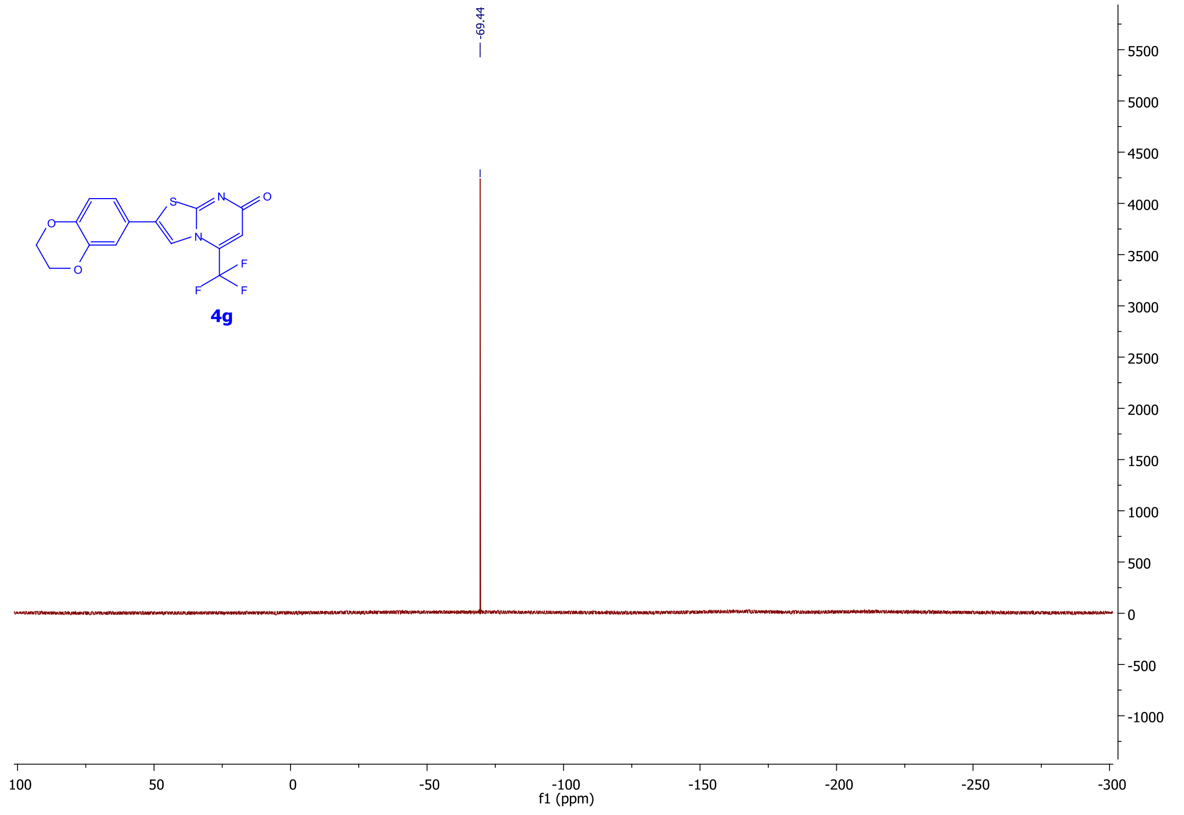

^{13}C NMR (75 MHz, $CDCl_3$)

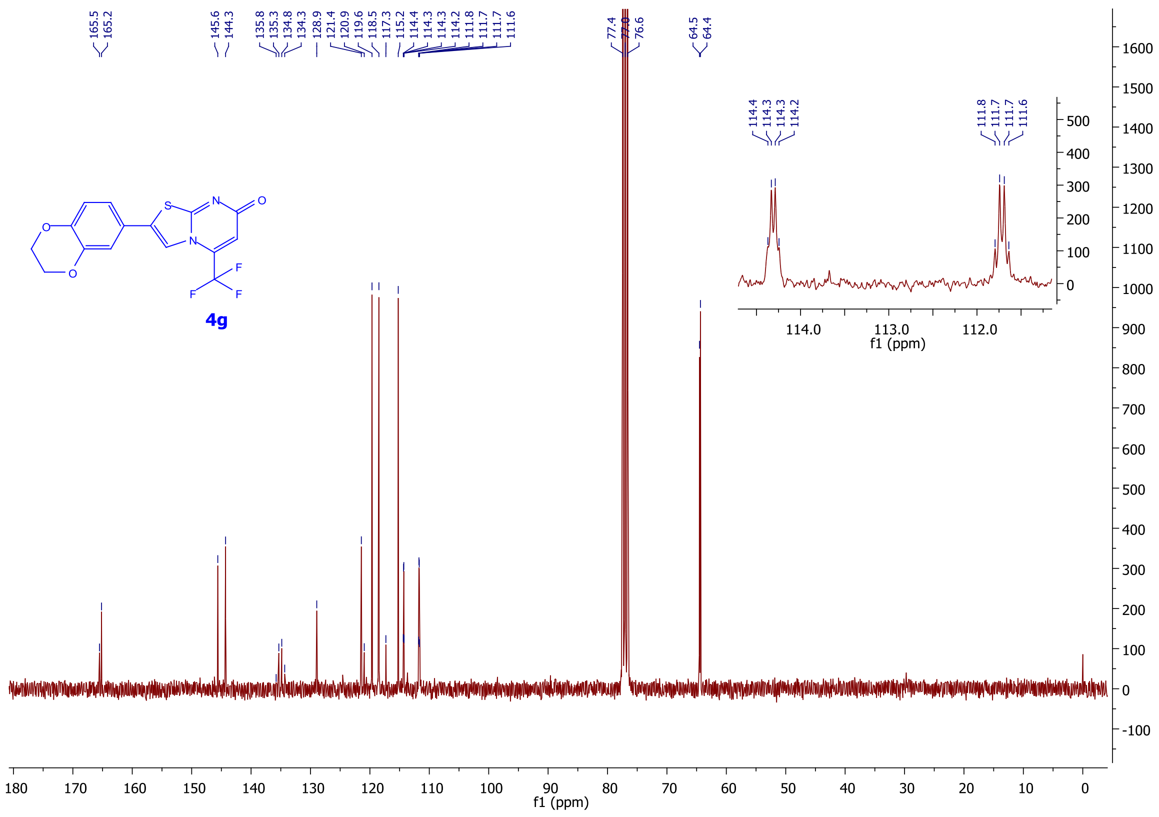

2-(3-aminophenyl)-5-(trifluoromethyl)-7*H*-thiazolo[3,2-*a*]pyrimidin-7-one (**4h**)

^{1}H NMR (300 MHz, DMSO-d_6)

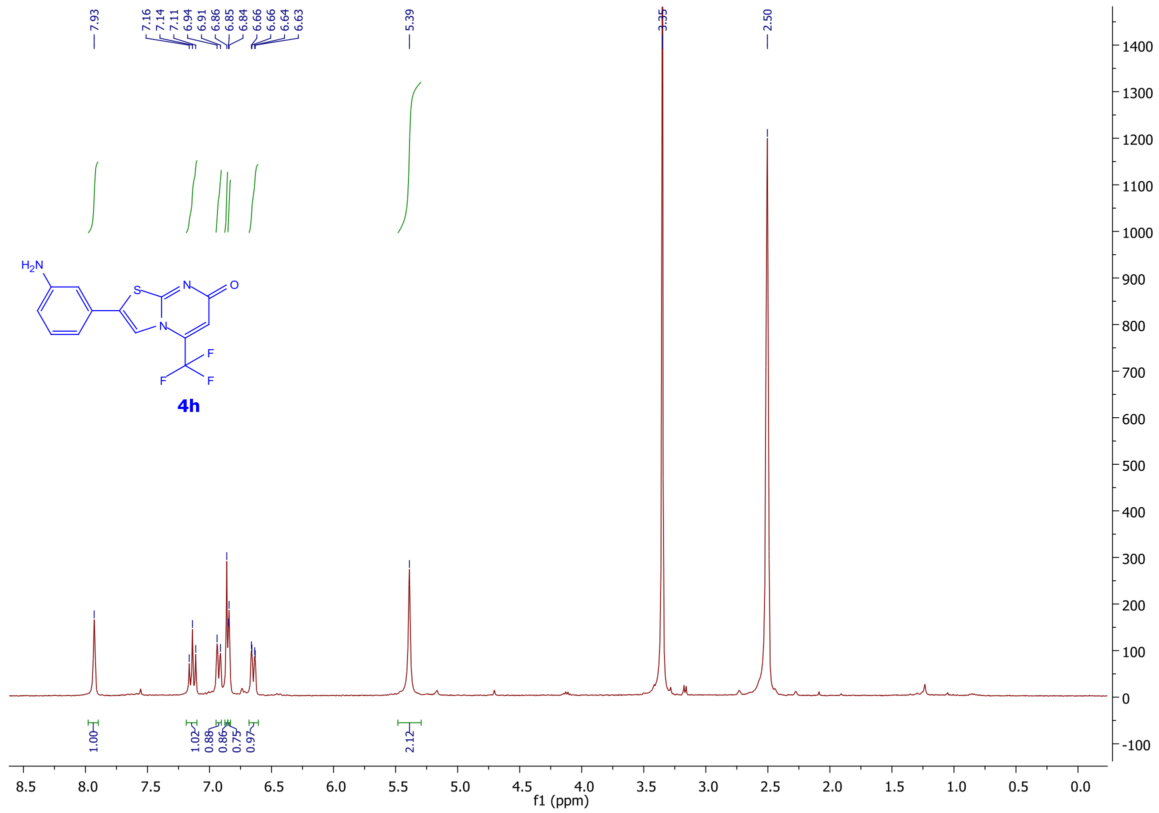

^{19}F NMR (282 MHz, DMSO-*d*$_6$)

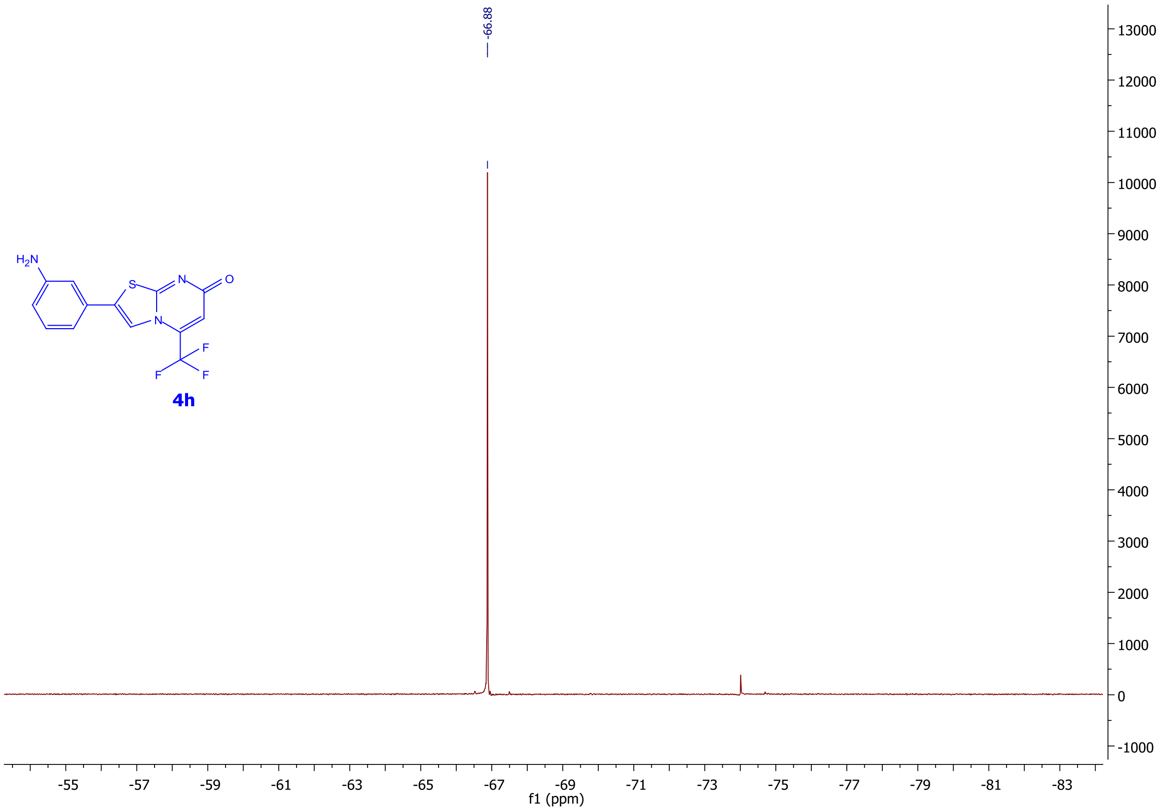

^{13}C NMR (75 MHz, DMSO-*d*$_6$)

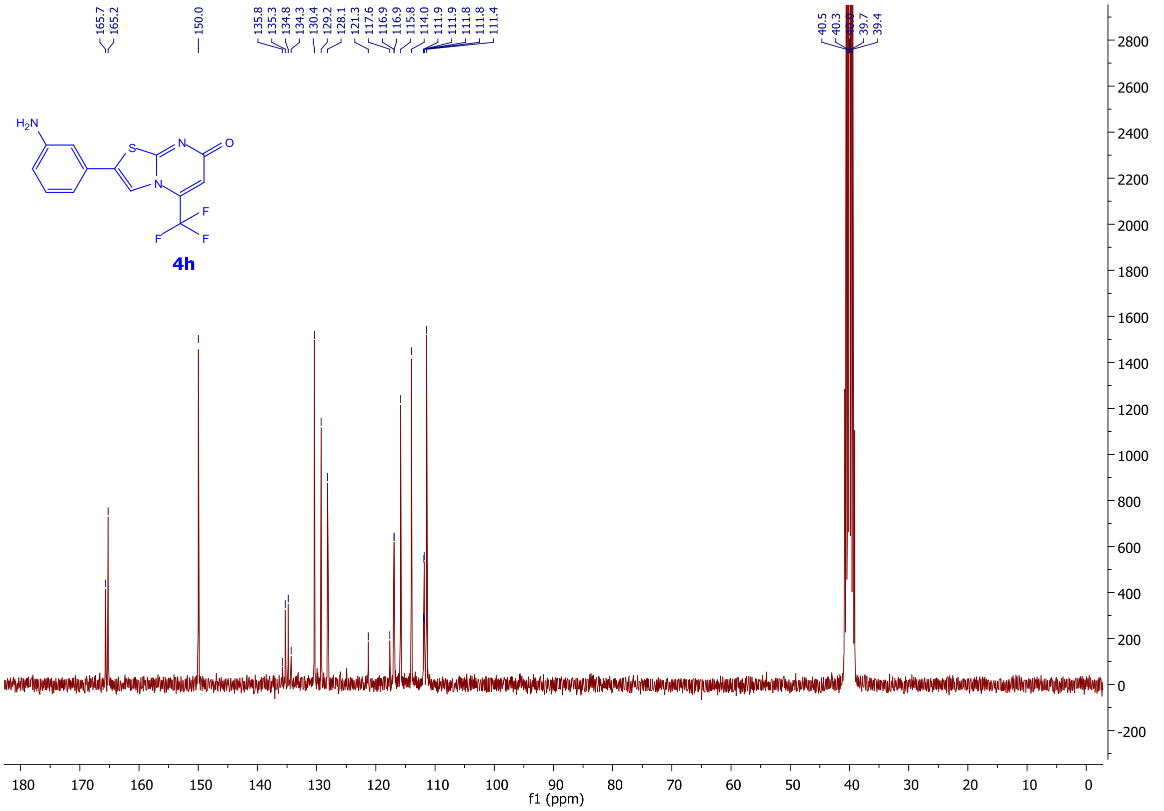

5-(trifluoromethyl)-2-(3-(trifluoromethyl)phenyl)-7*H*-thiazolo[3,2-*a*]pyrimidin-7-one (**4i**)

^{1}H NMR (300 MHz, MeOH-*d*$_4$)

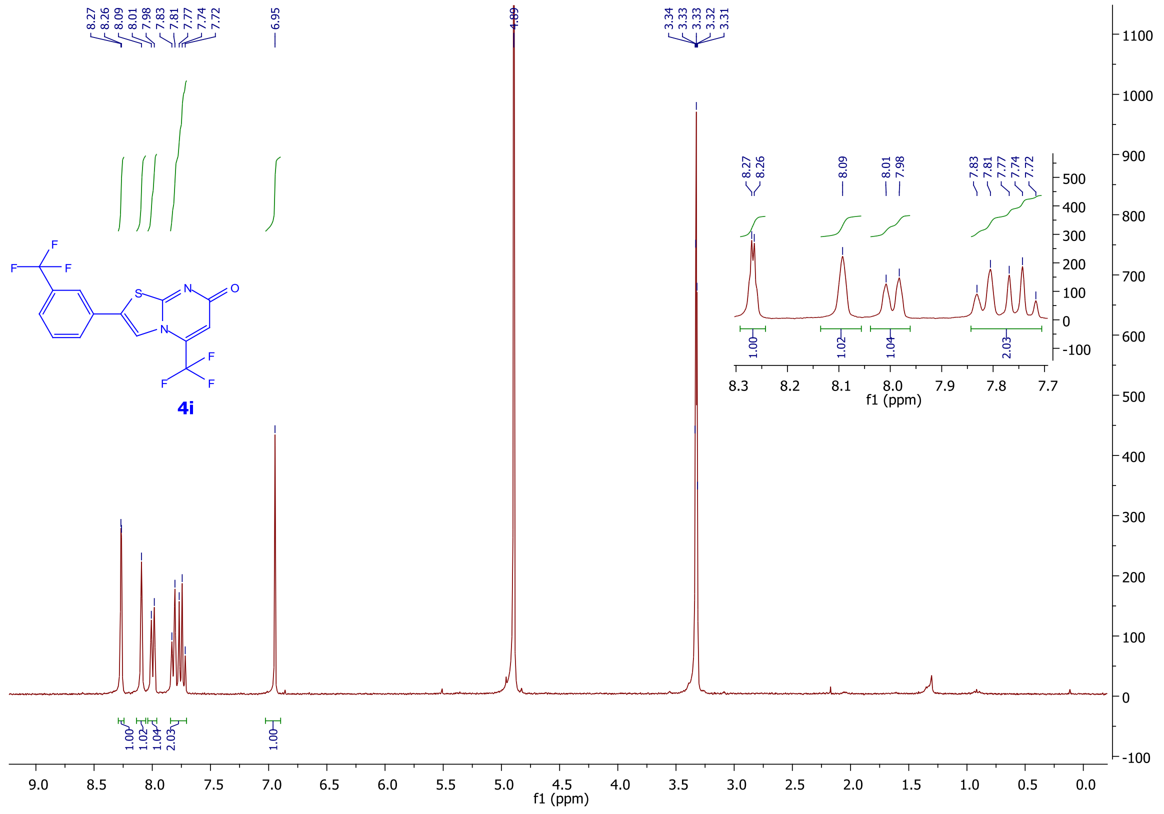

^{19}F NMR (282 MHz, MeOH-*d*$_4$)

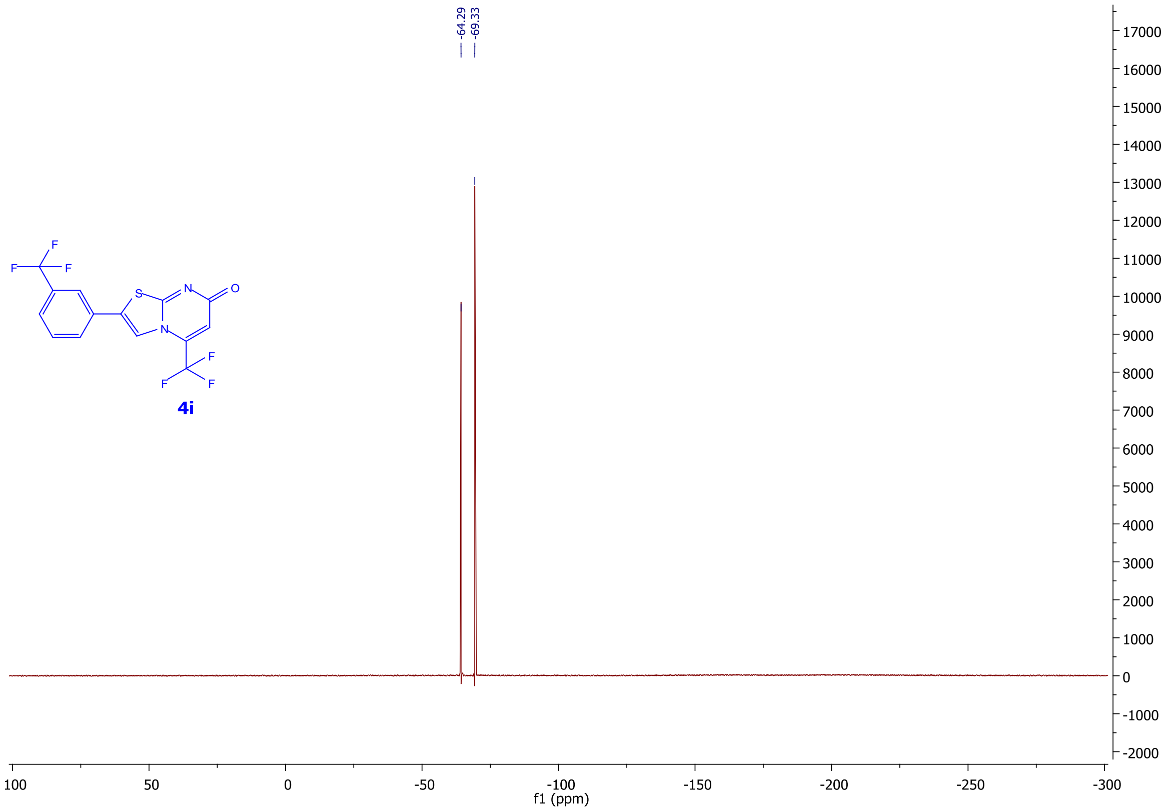

^{13}C NMR (75 MHz, DMSO-d_6)

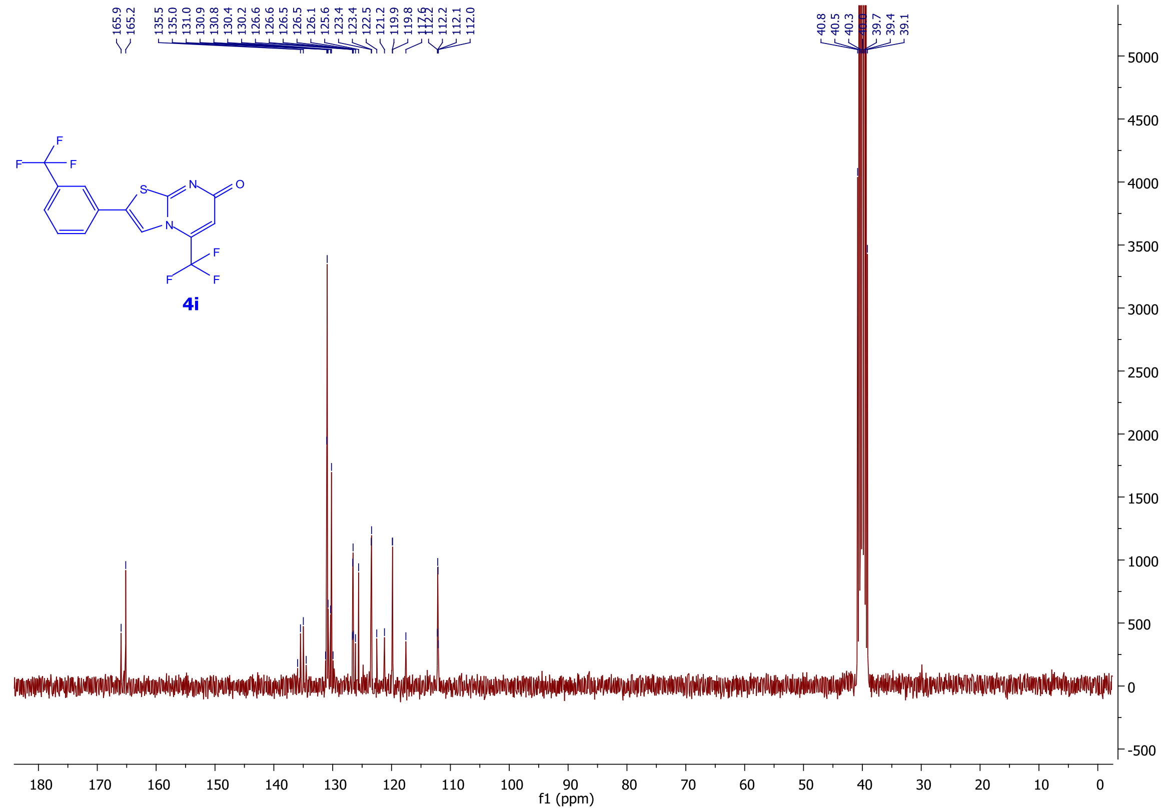

5-(trifluoromethyl)-2-(4-(trifluoromethyl)phenyl)-7*H*-thiazolo[3,2-*a*]pyrimidin-7-one (**4j**)

^{1}H NMR (300 MHz, MeOH-d_4)

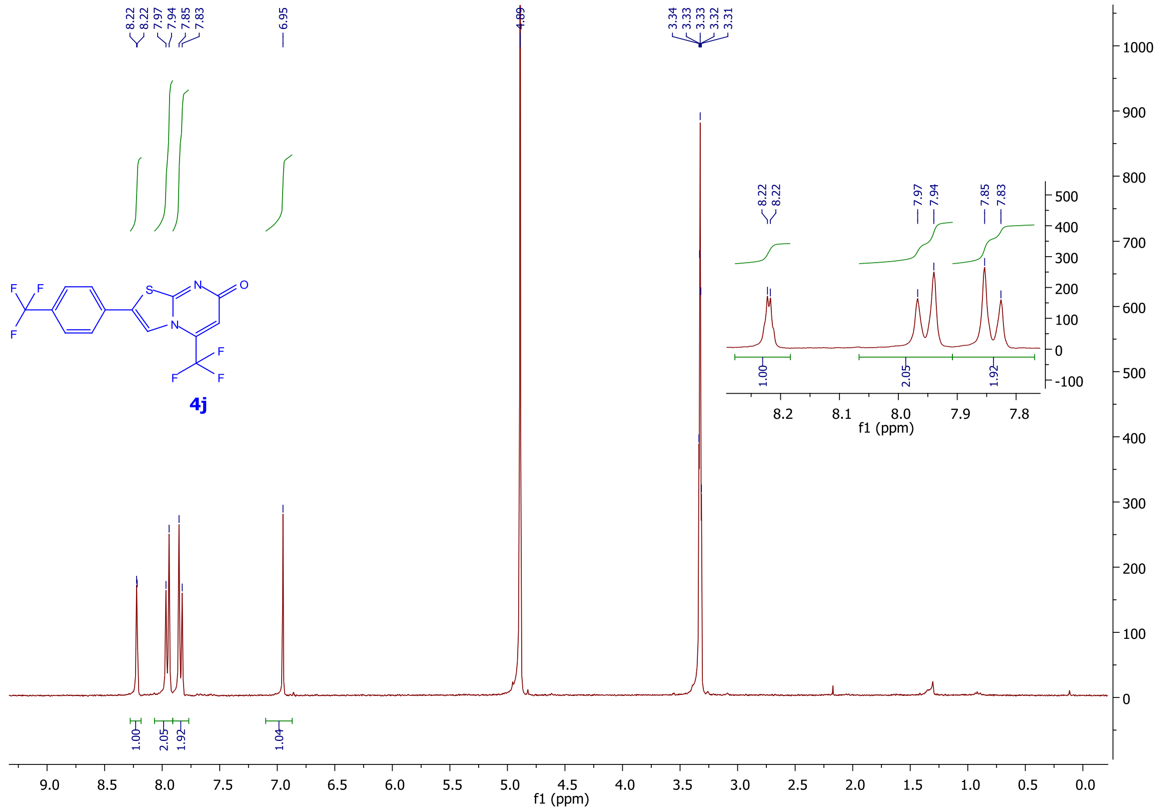

^{19}F NMR (282 MHz, MeOH-d_4)

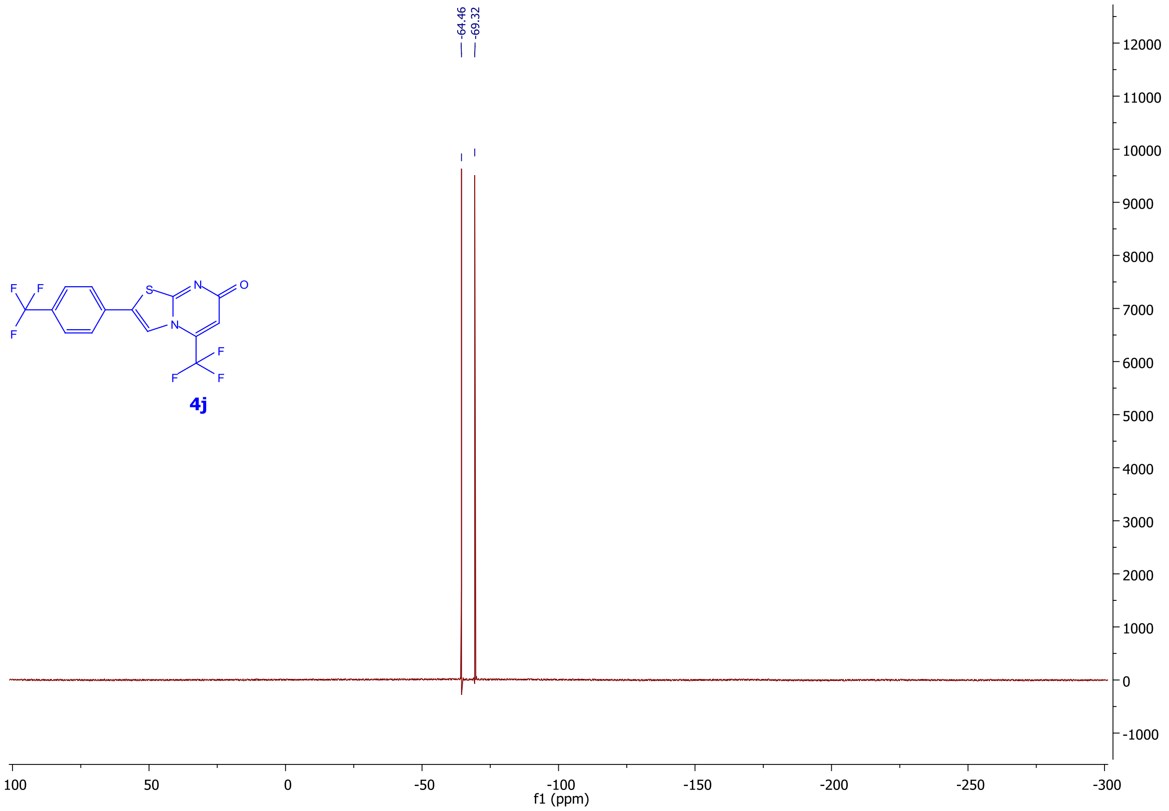

^{13}C NMR (75 MHz, DMSO-d_6)

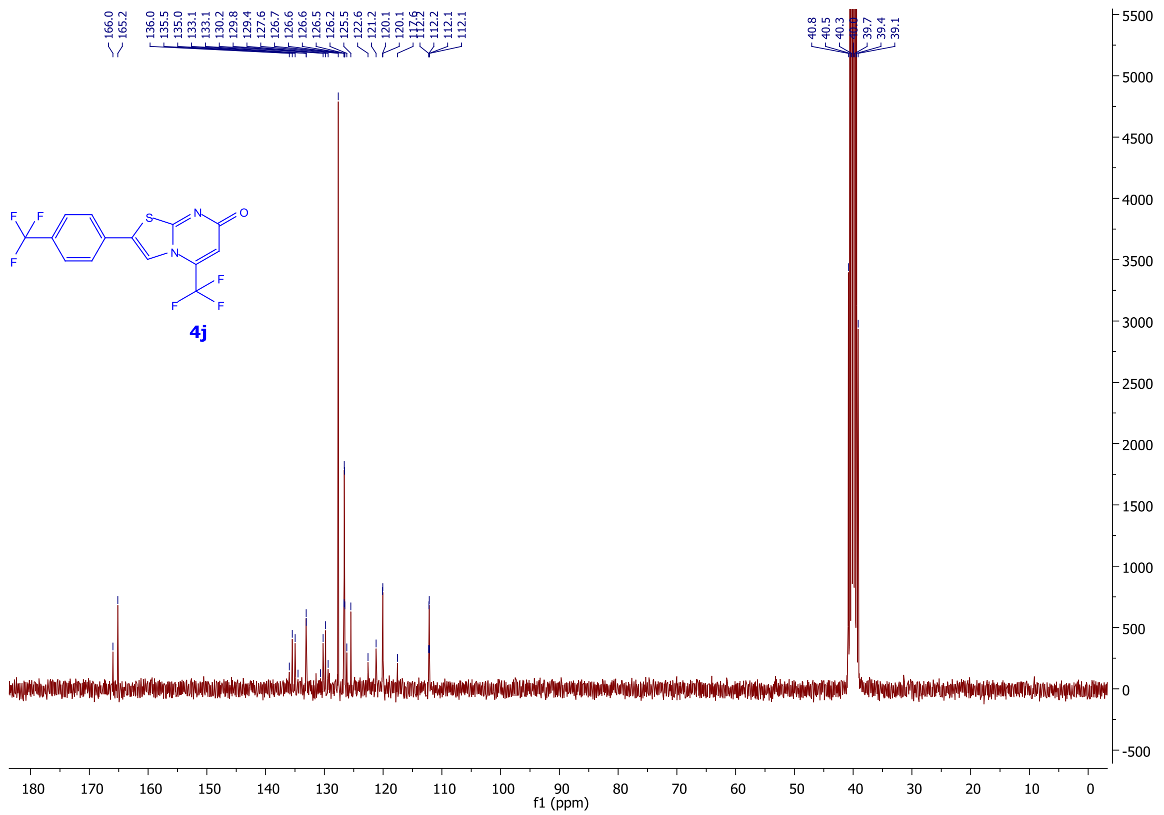

Ethyl 4-(7-oxo-5-(trifluoromethyl)-7*H*-thiazolo[3,2-*a*]pyrimidin-2-yl)benzoate (**4k**)

^{1}H NMR (300 MHz, $CDCl_3$)

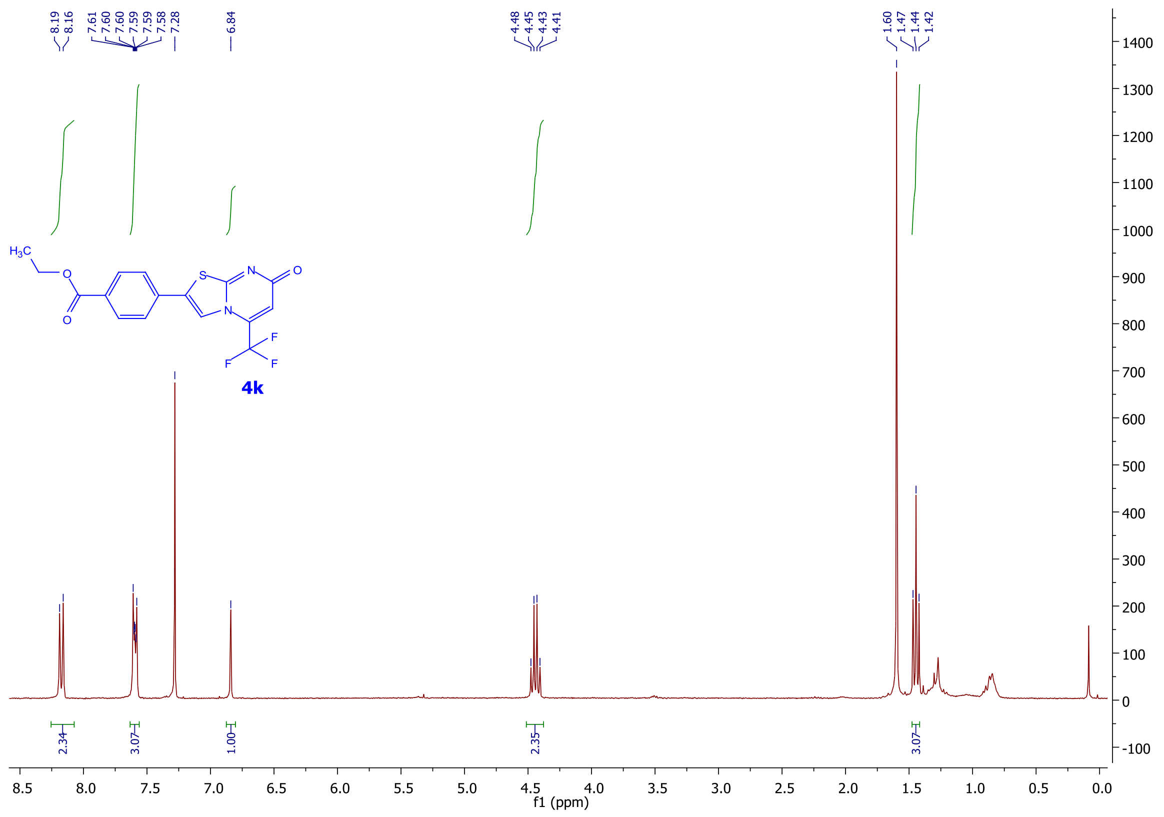

^{19}F NMR (282 MHz, CDCl$_3$)

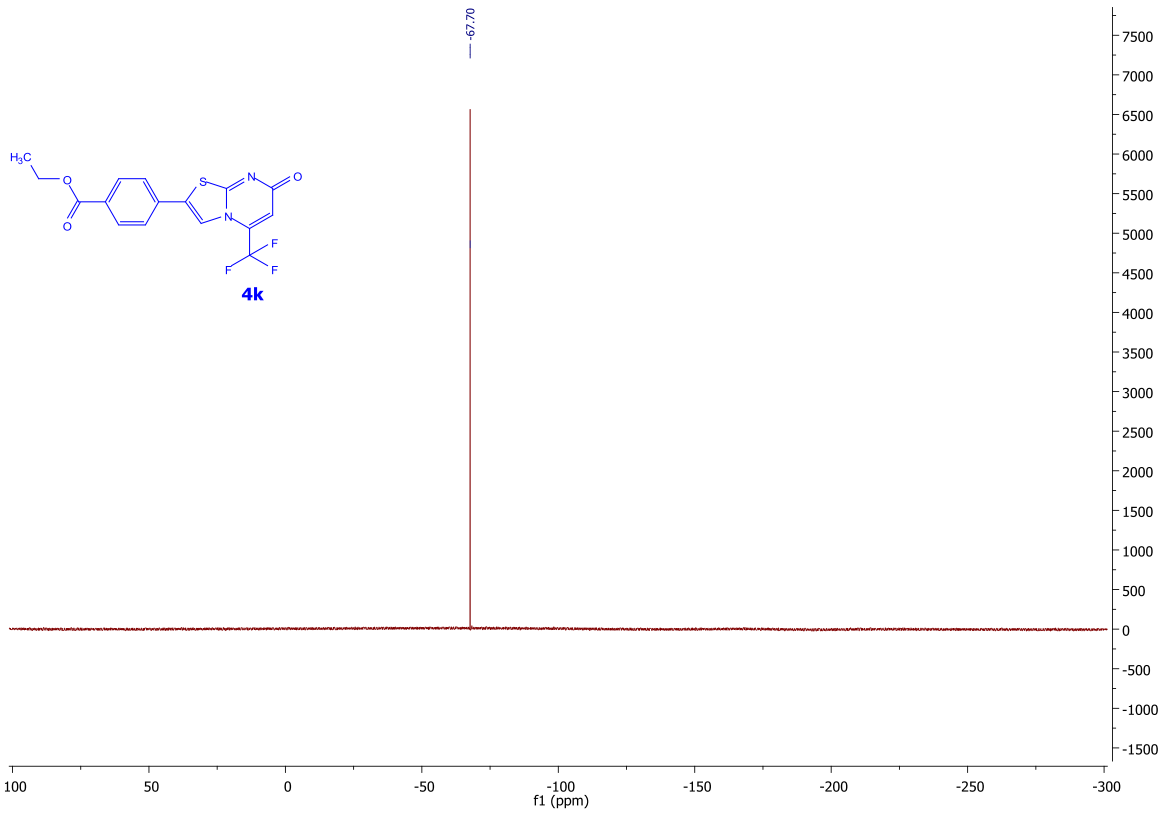

^{13}C NMR (75 MHz, DMSO-d_6)

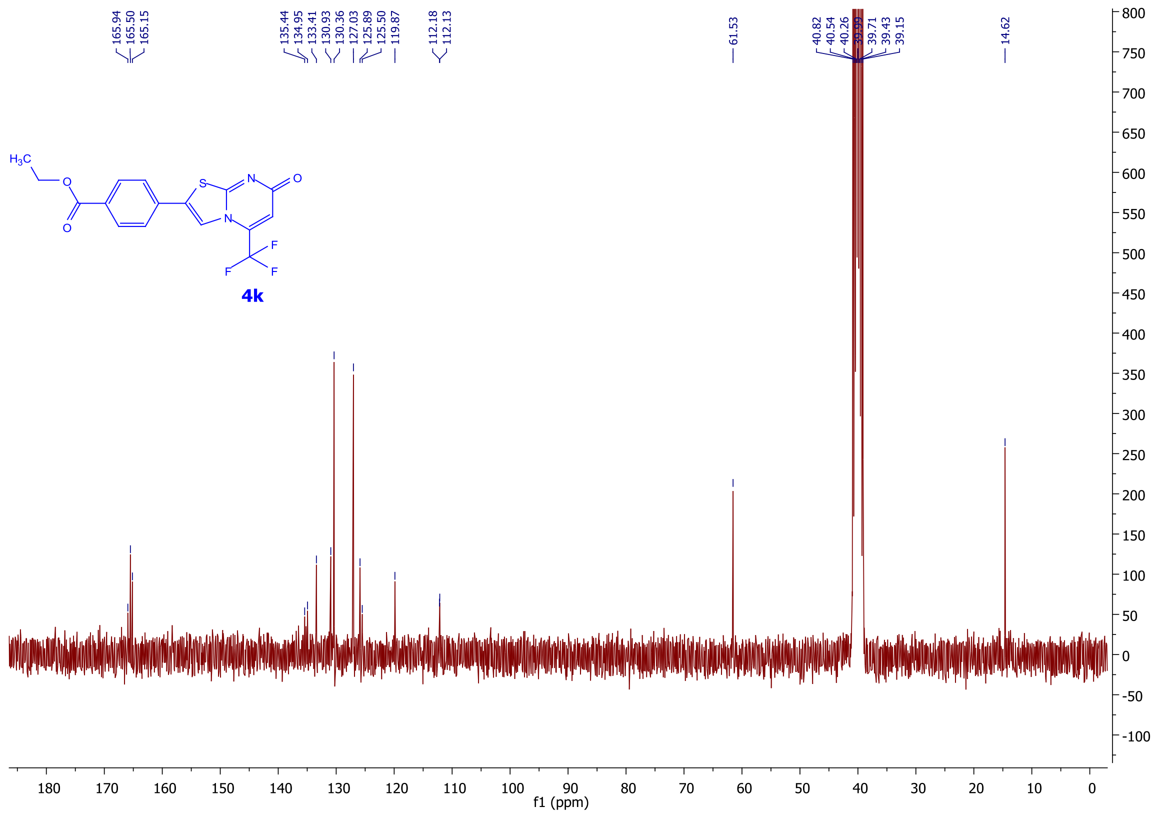

2-(thiophen-3-yl)-5-(trifluoromethyl)-7*H*-thiazolo[3,2-*a*]pyrimidin-7-one (**4l**)

^{1}H NMR (300 MHz, MeOH-*d*$_4$)

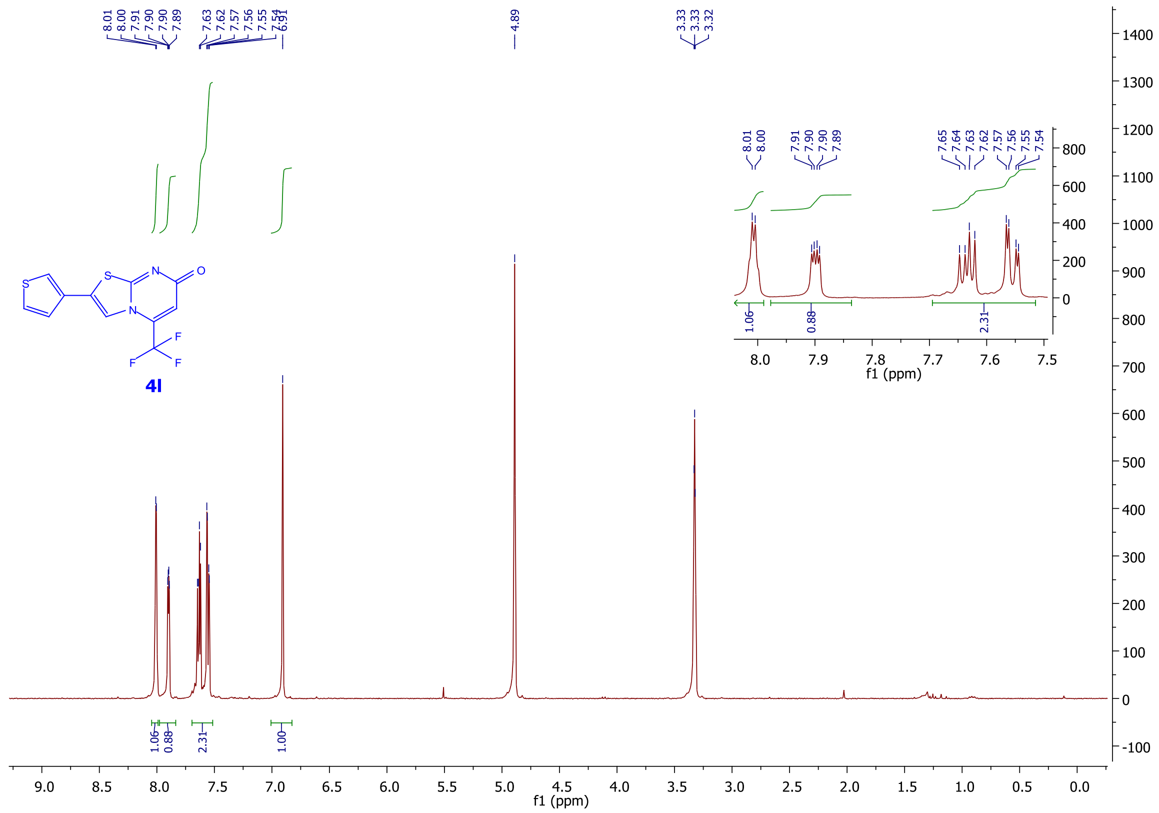

^{19}F NMR (282 MHz, MeOH-d_4)

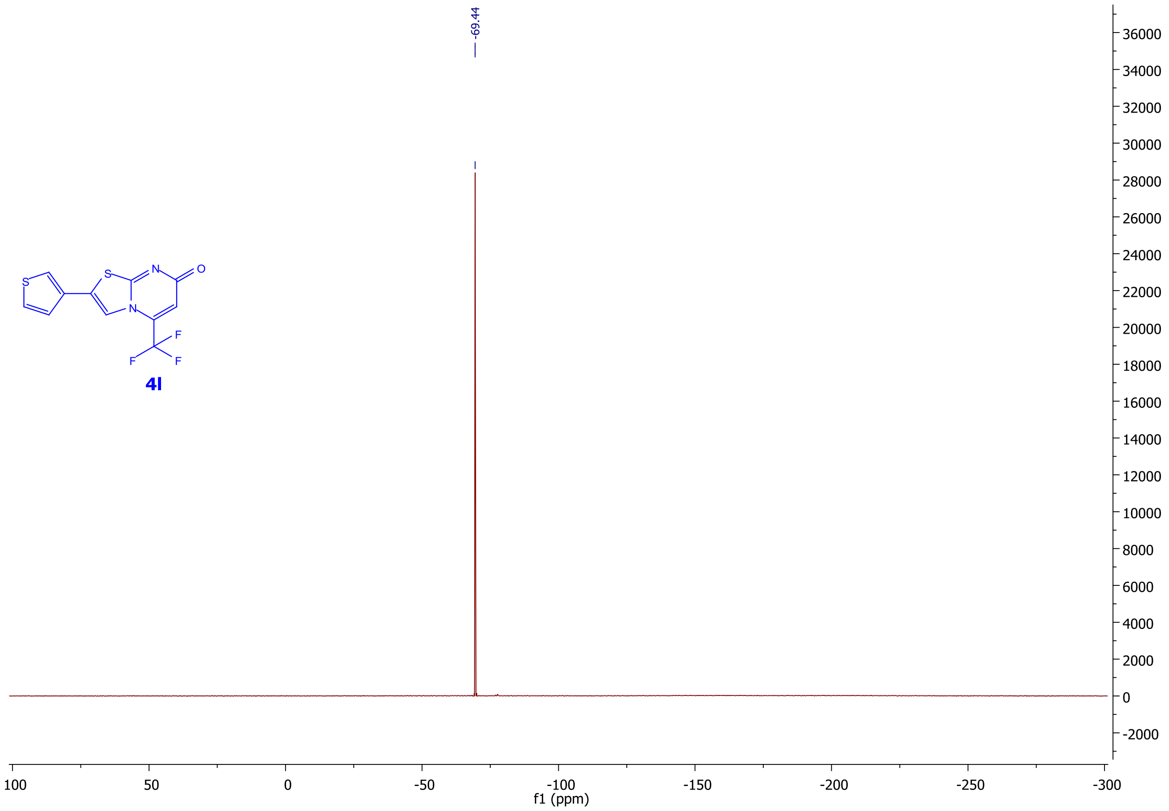

^{13}C NMR (75 MHz, MeOH-d_4)

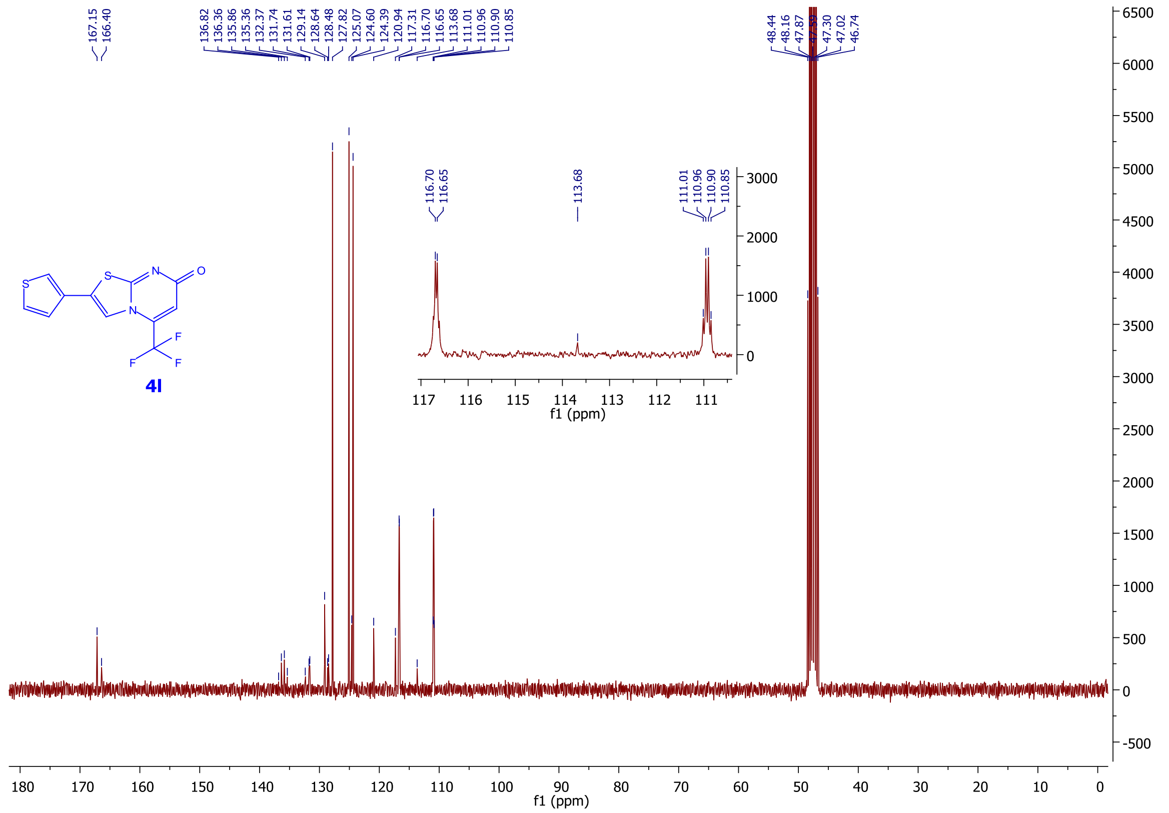

2-(phenylethynyl)-5-(trifluoromethyl)-7H-thiazolo[3,2-a]pyrimidin-7-one (**5a**)

^{1}H NMR (300 MHz, $CDCl_3$)

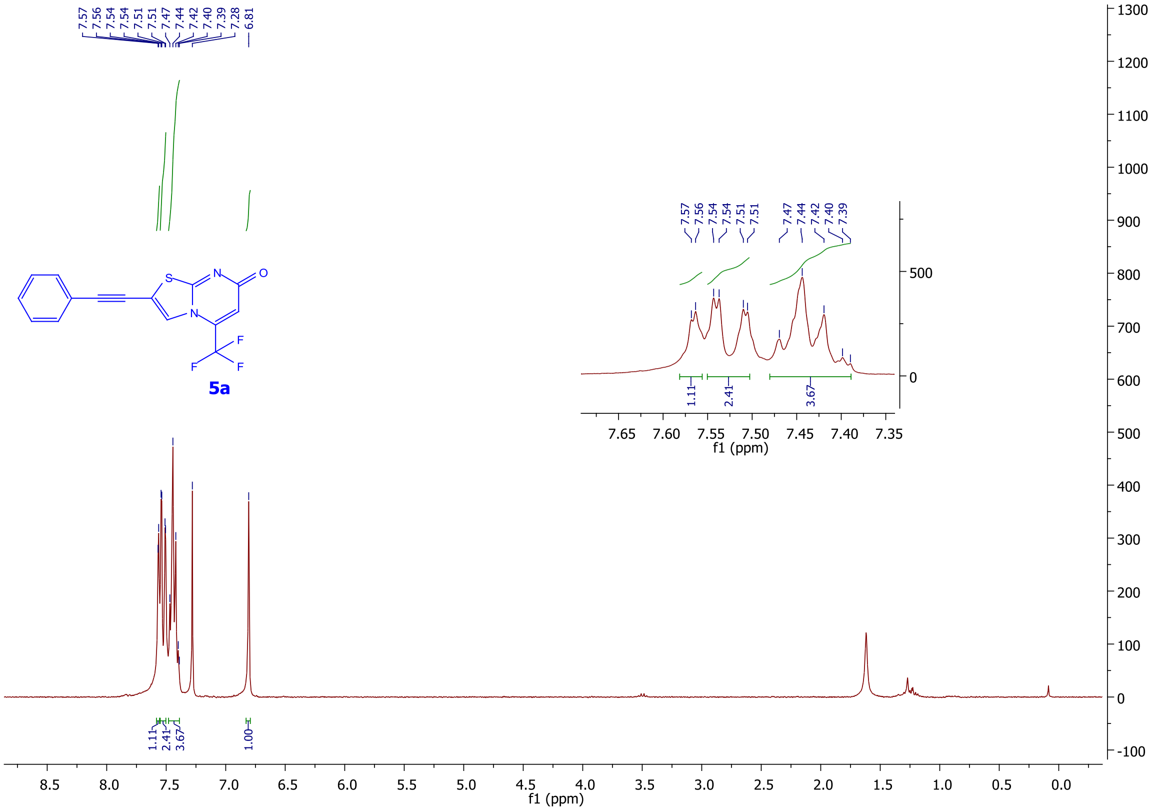

^{19}F NMR (282 MHz, $CDCl_3$)

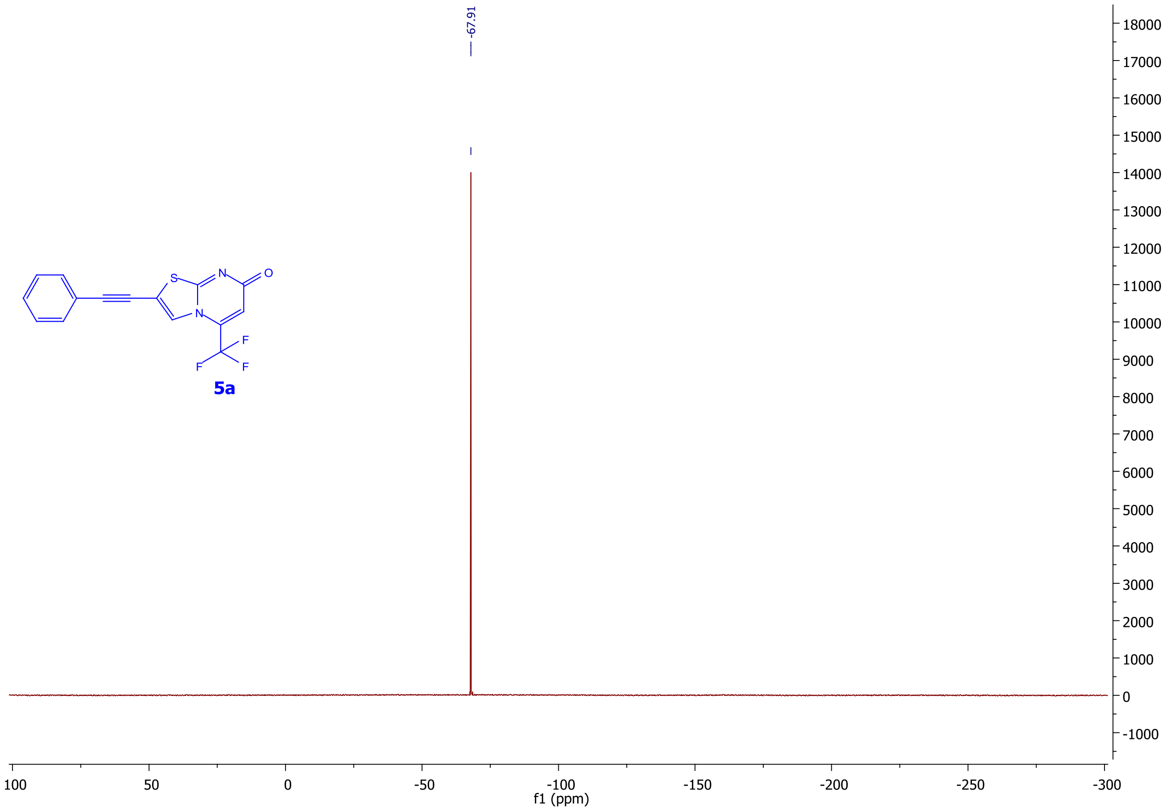

^{13}C NMR (75 MHz, $CDCl_3$)

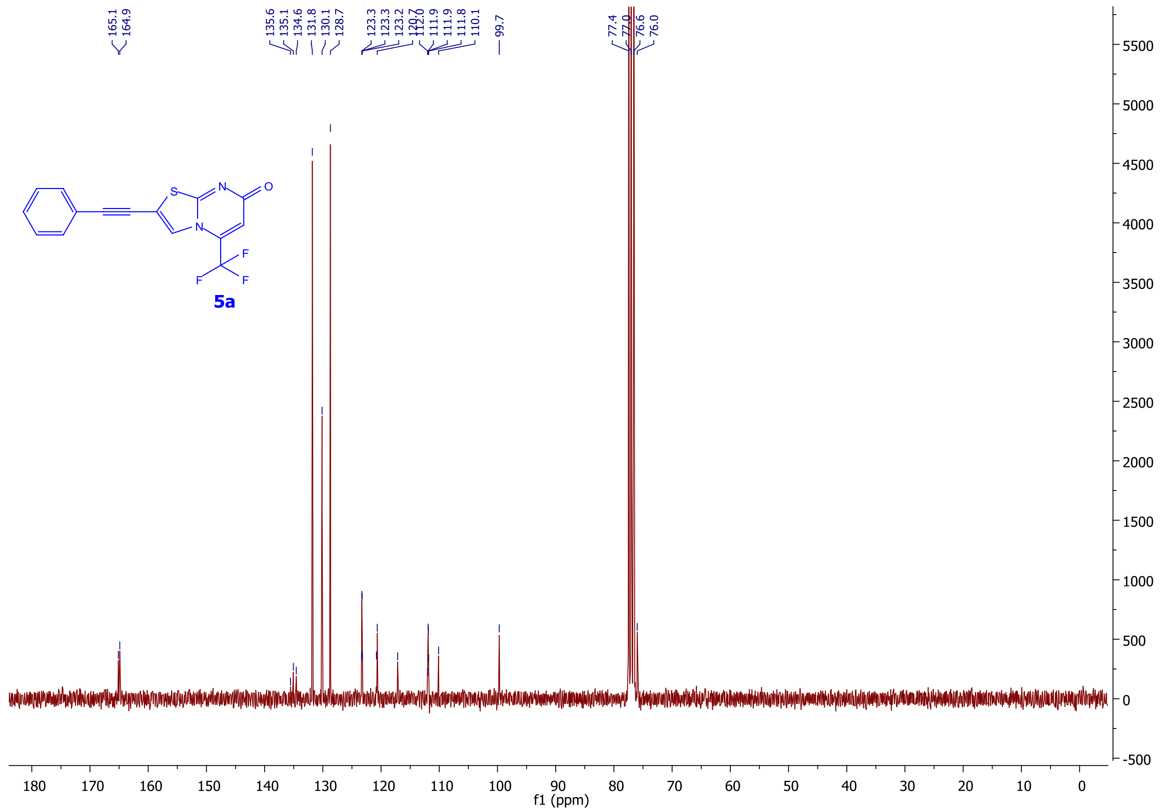

2-((4-methoxyphenyl)ethynyl)-5-(trifluoromethyl)-7*H*-thiazolo[3,2-*a*]pyrimidin-7-one (**5b**)

^{1}H NMR (300 MHz, $CDCl_3$)

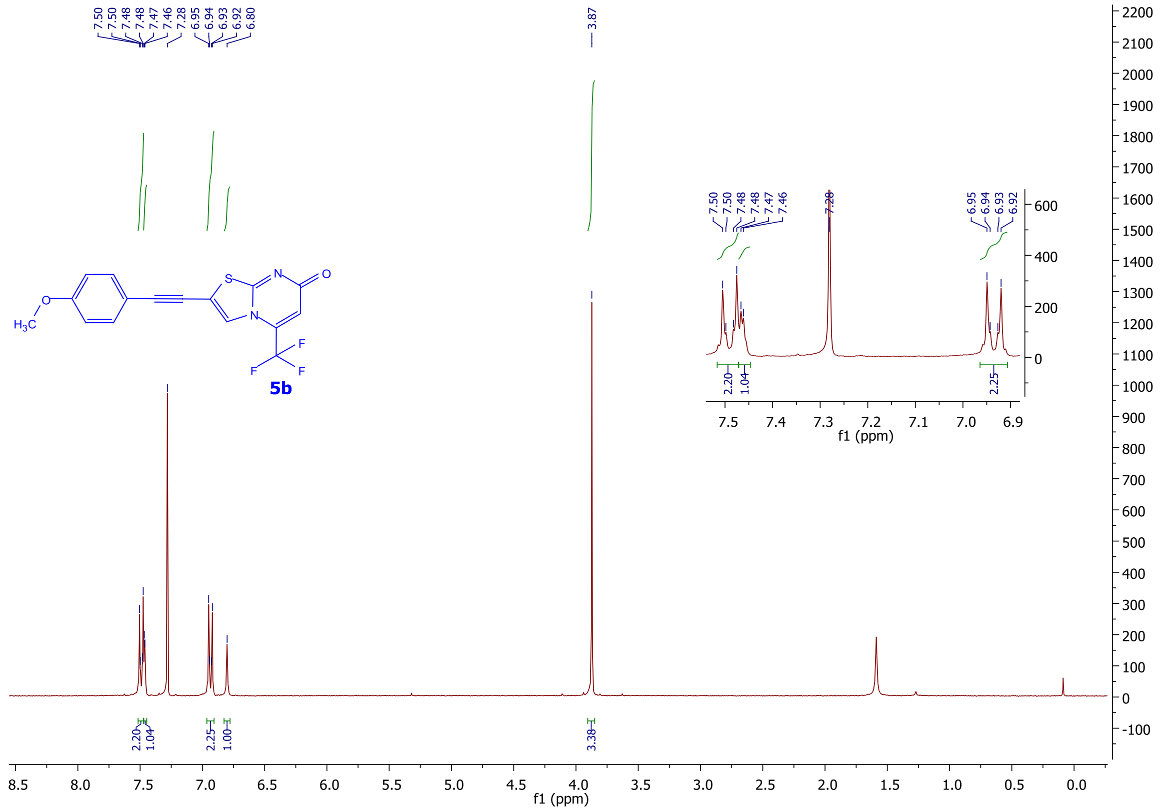

^{19}F NMR (282 MHz, $CDCl_3$)

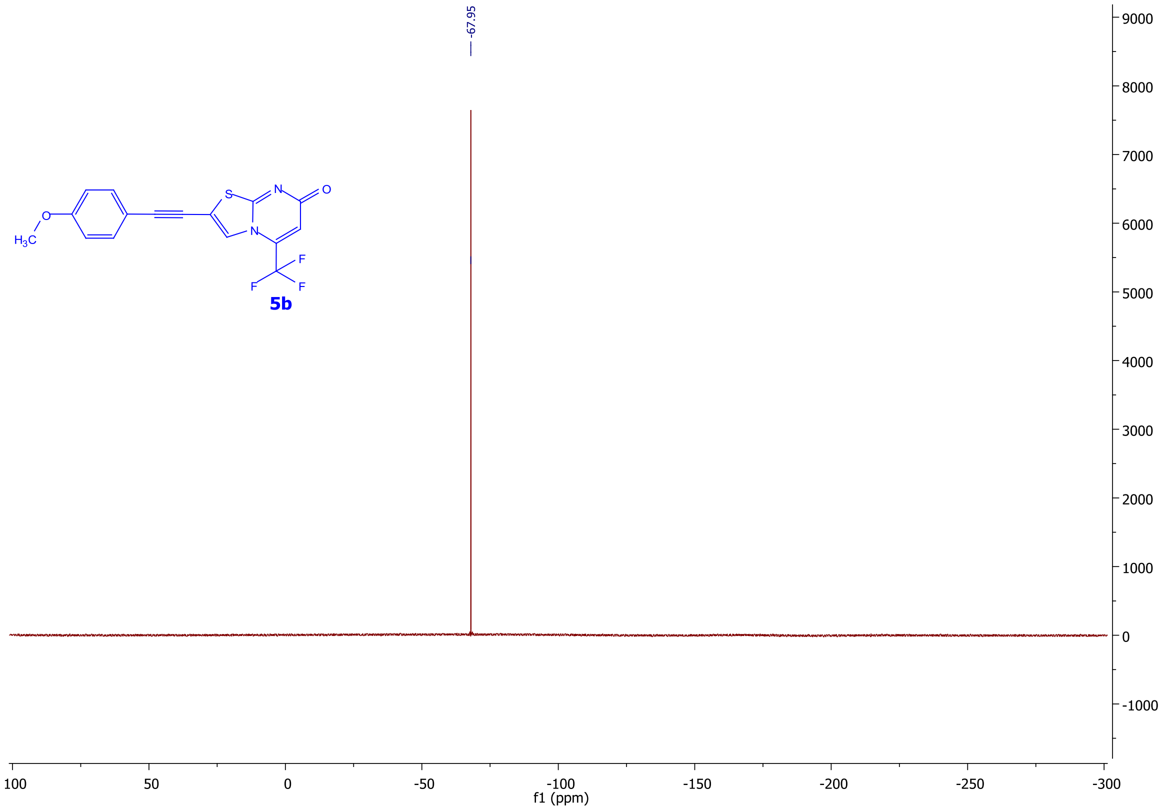

^{13}C NMR (75 MHz, $CDCl_3$)

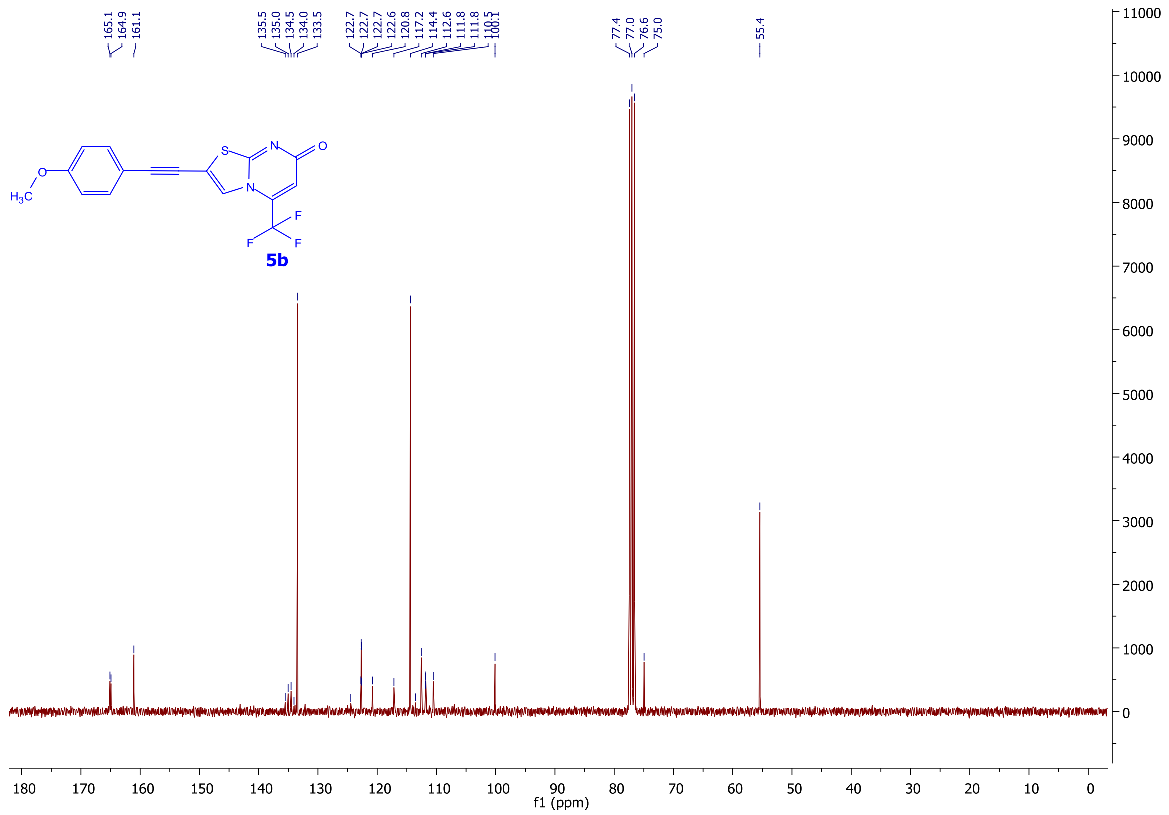

2-((4-chlorophenyl)ethynyl)-5-(trifluoromethyl)-7*H*-thiazolo[3,2-*a*]pyrimidin-7-one (**5c**)

^{1}H NMR (300 MHz, $CDCl_3$)

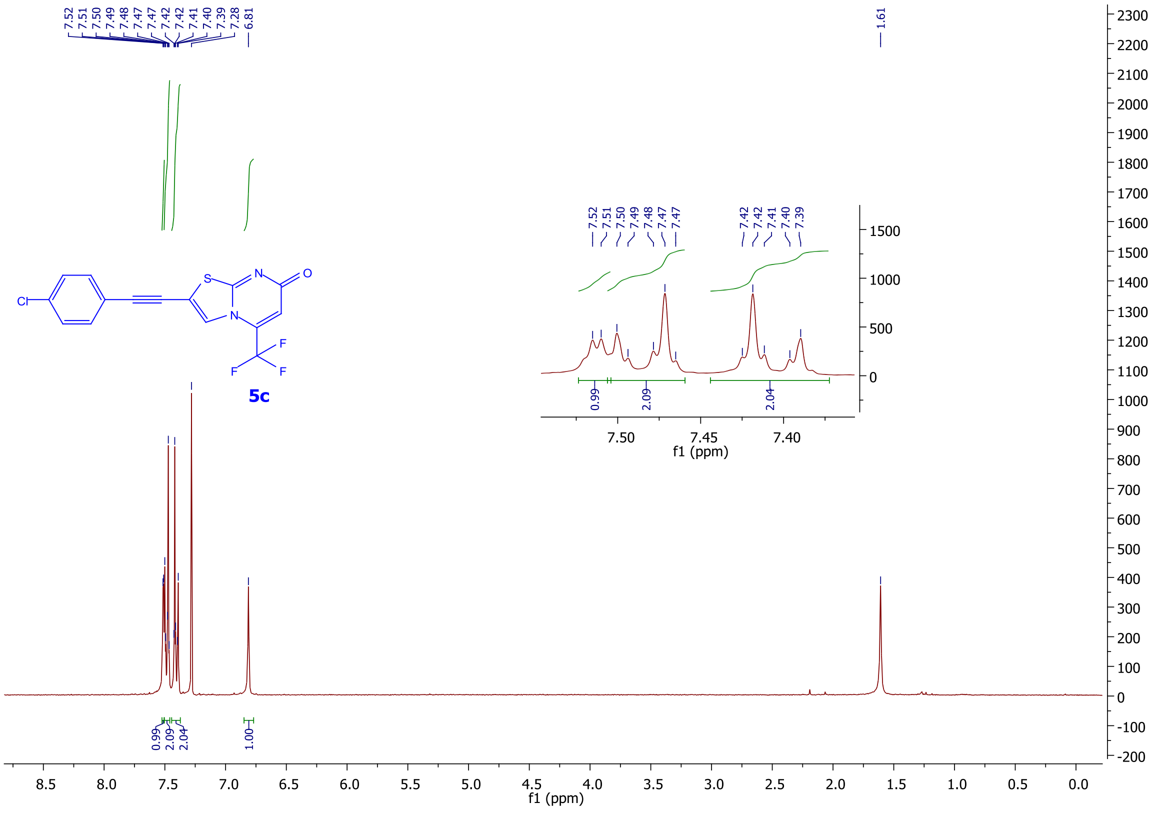

^{19}F NMR (282 MHz, $CDCl_3$)

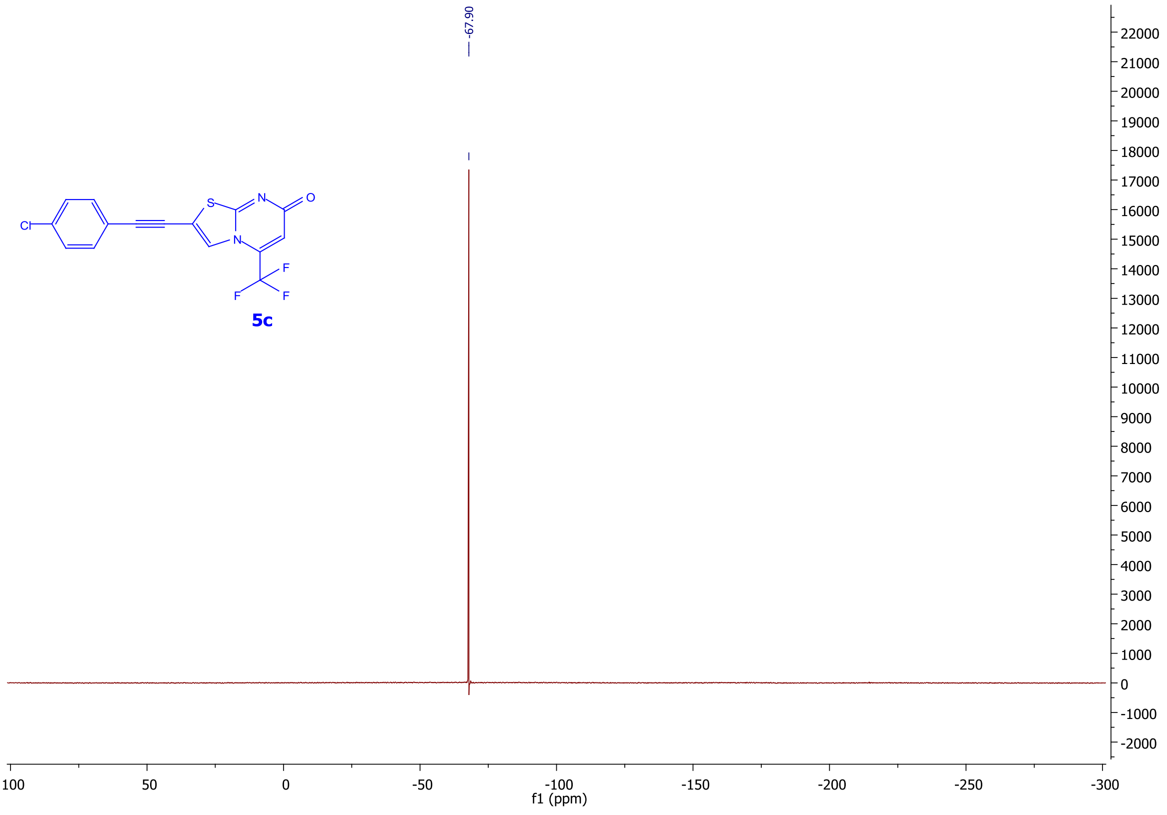

^{13}C NMR (75 MHz, $CDCl_3$)

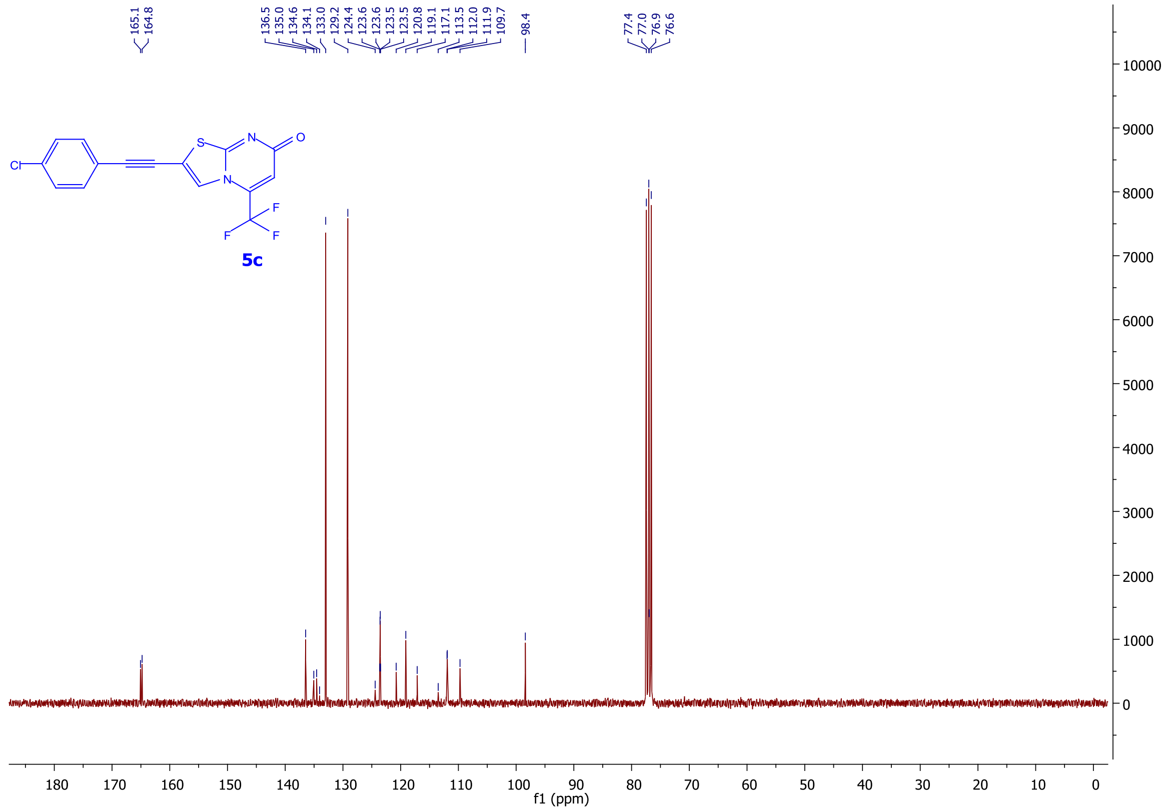

2-((4-fluorophenyl)ethynyl)-5-(trifluoromethyl)-7*H*-thiazolo[3,2-*a*]pyrimidin-7-one (**5d**)

^{1}H NMR (300 MHz, $CDCl_3$)

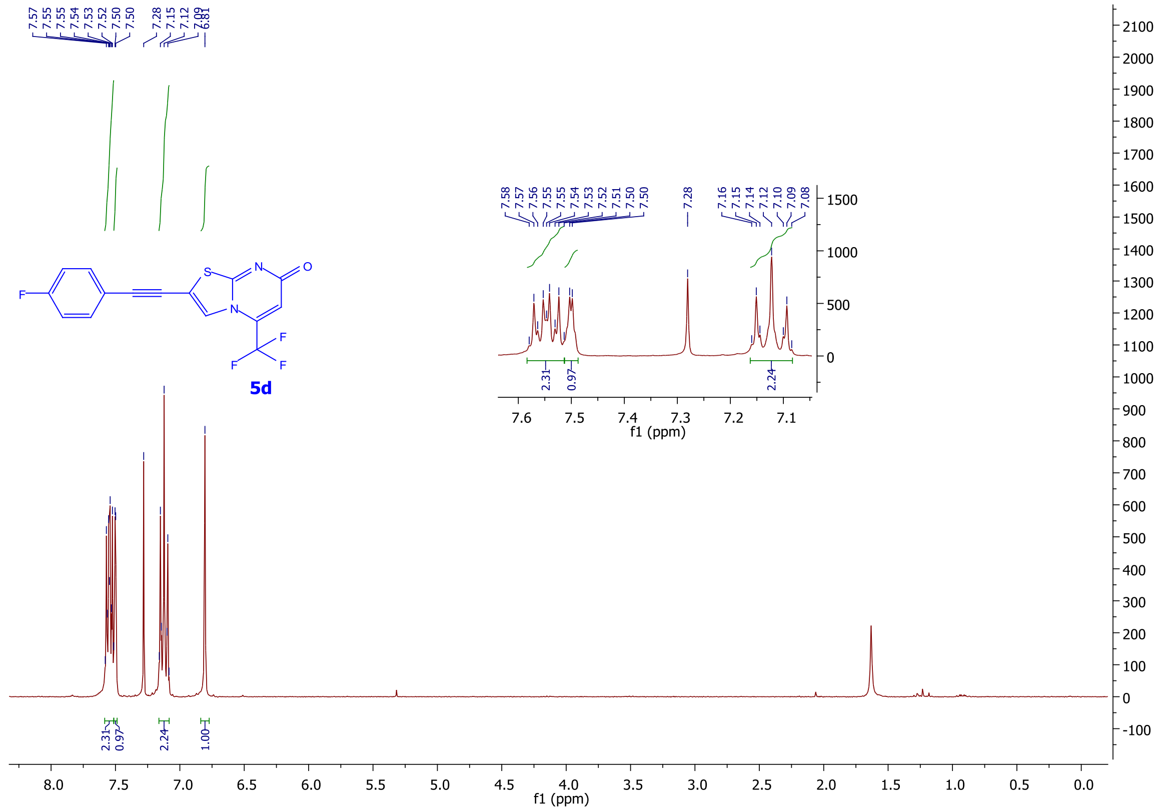

^{19}F NMR (282 MHz, $CDCl_3$)

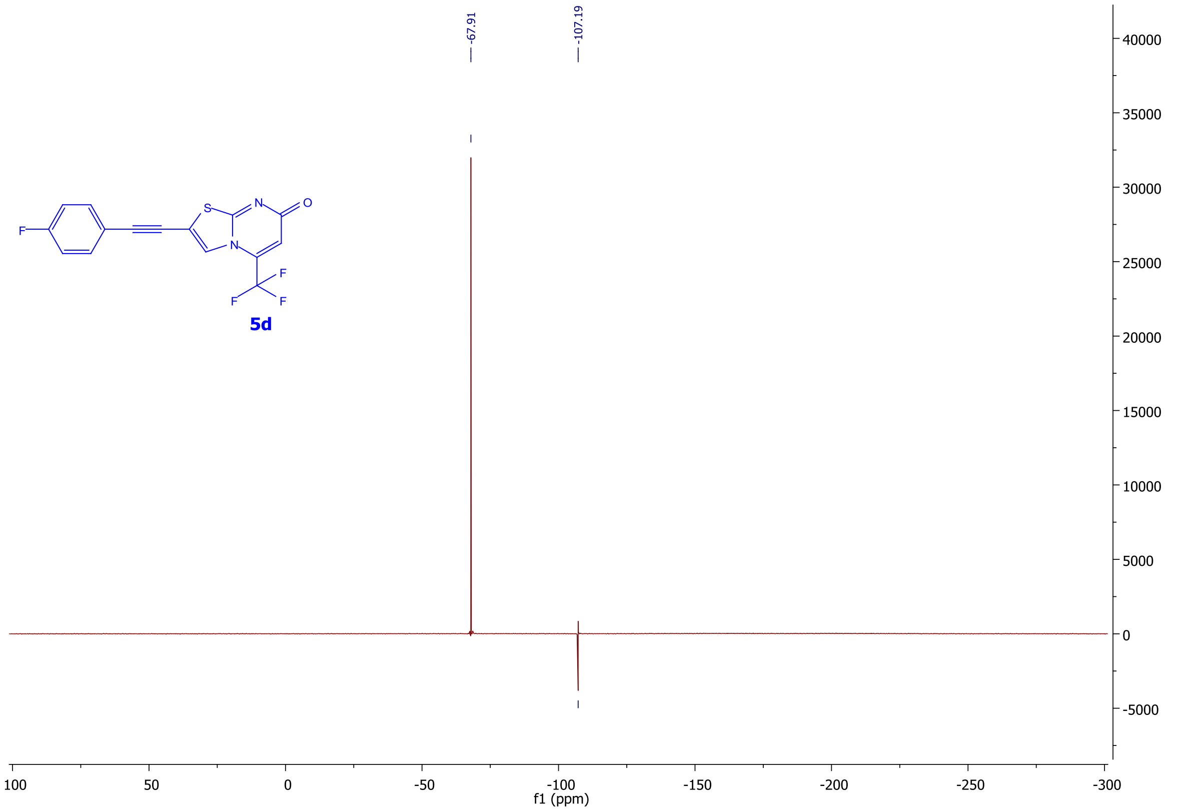

^{13}C NMR (75 MHz, $CDCl_3$)

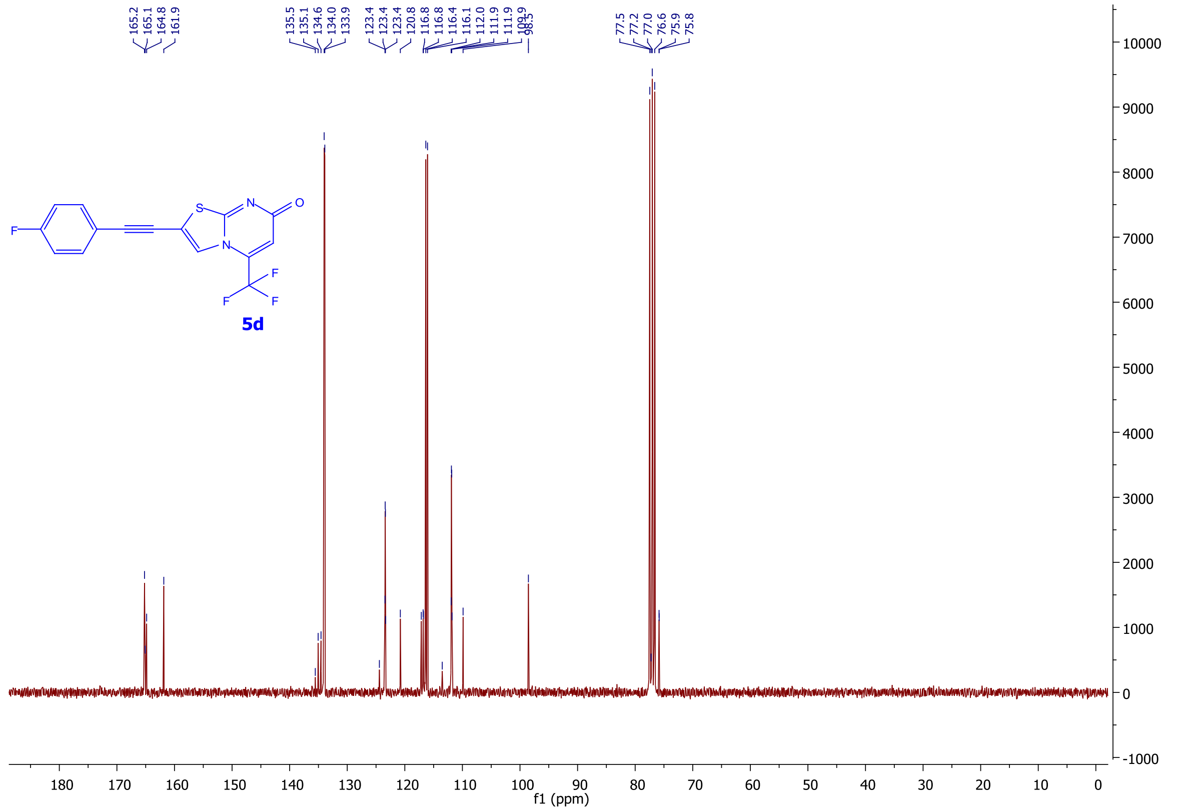

2-((2-fluorophenyl)ethynyl)-5-(trifluoromethyl)-7*H*-thiazolo[3,2-*a*]pyrimidin-7-one (**5e**)

^{1}H NMR (300 MHz, $CDCl_3$)

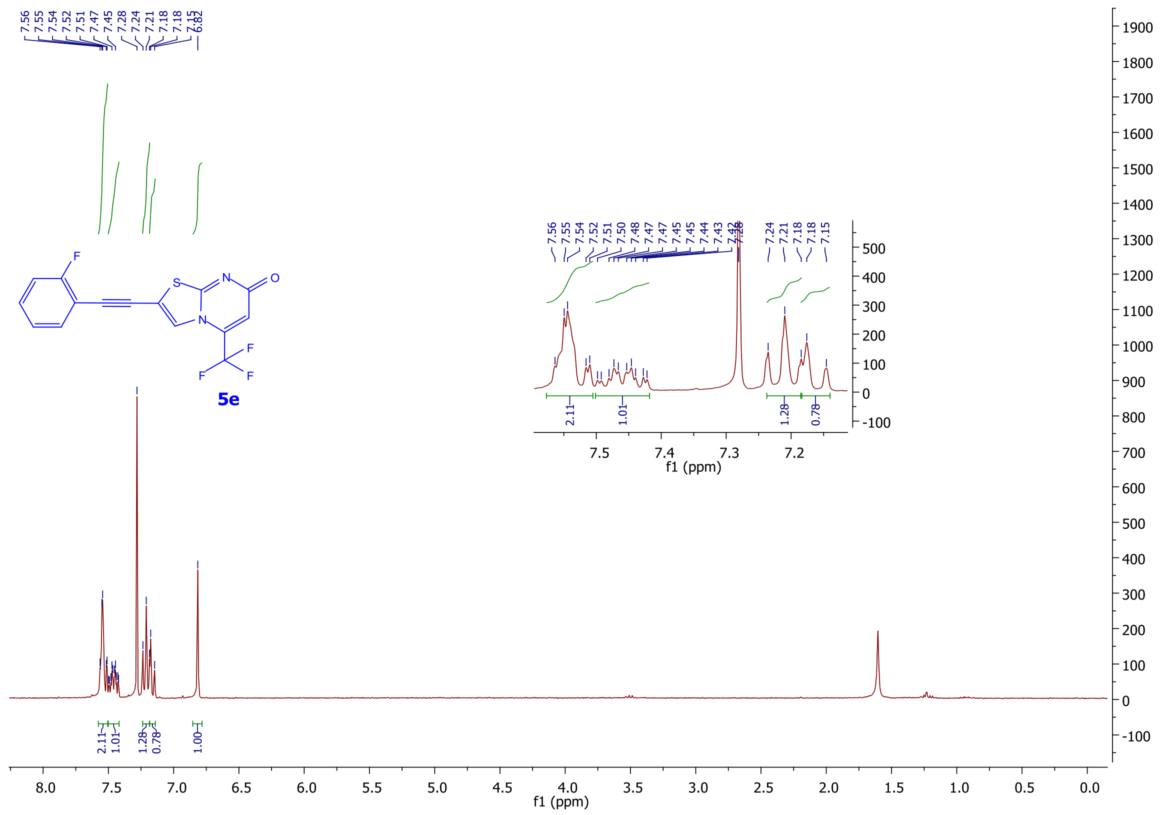

^{19}F NMR (282 MHz, $CDCl_3$)

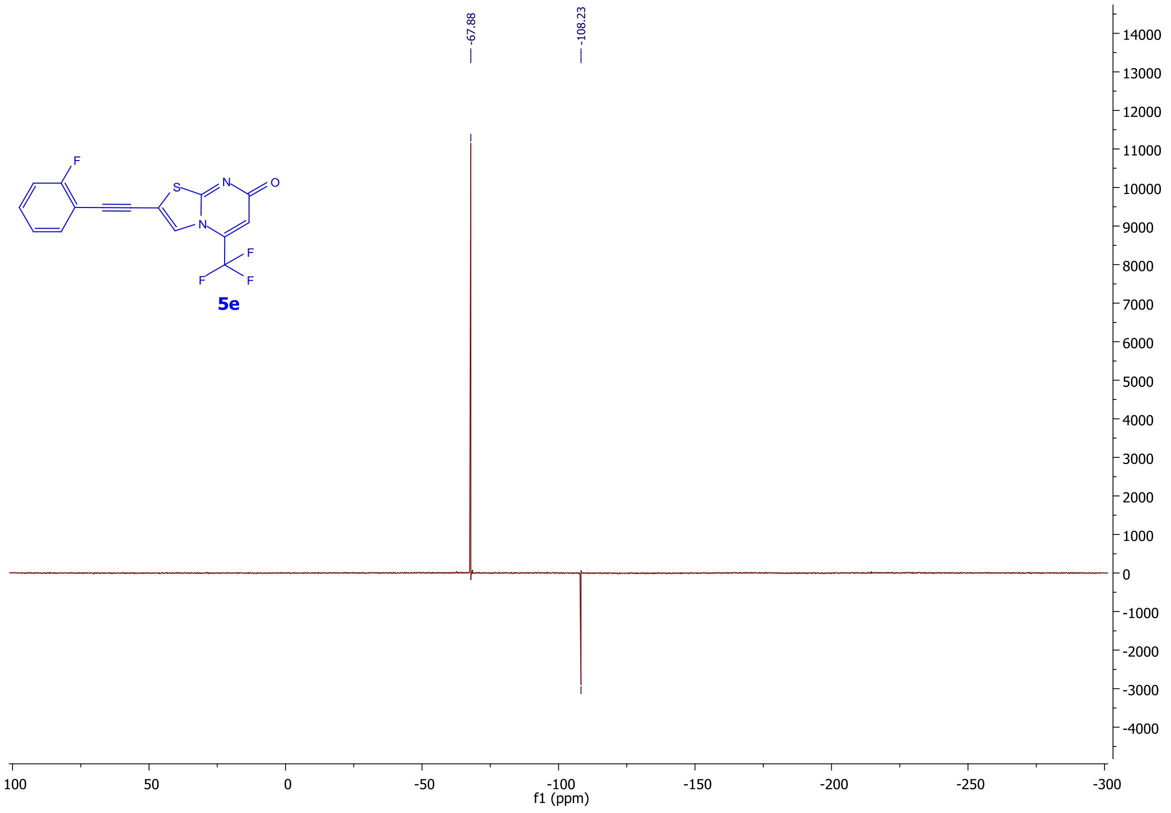

^{13}C NMR (75 MHz, $CDCl_3$)

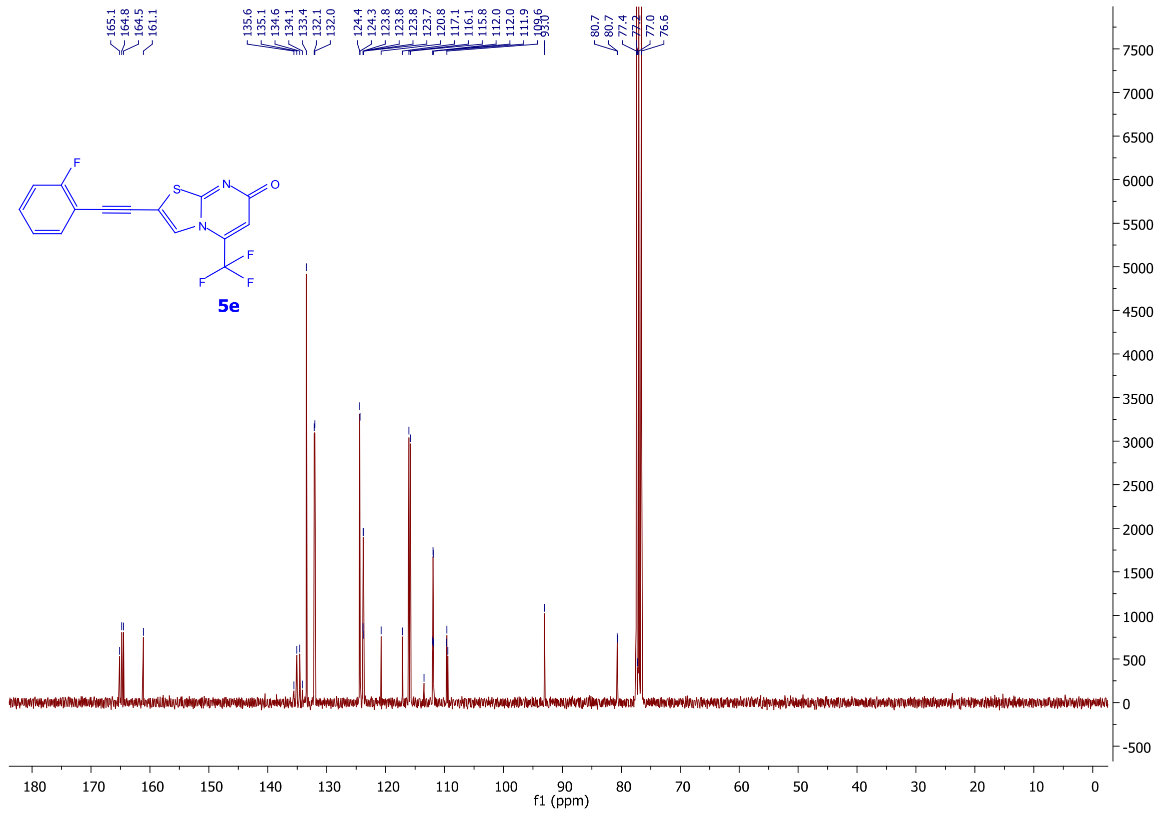

2-(pyridin-2-ylethynyl)-5-(trifluoromethyl)-7*H*-thiazolo[3,2-*a*]pyrimidin-7-one (**5f**)

^{1}H NMR (300 MHz, $CDCl_3$)

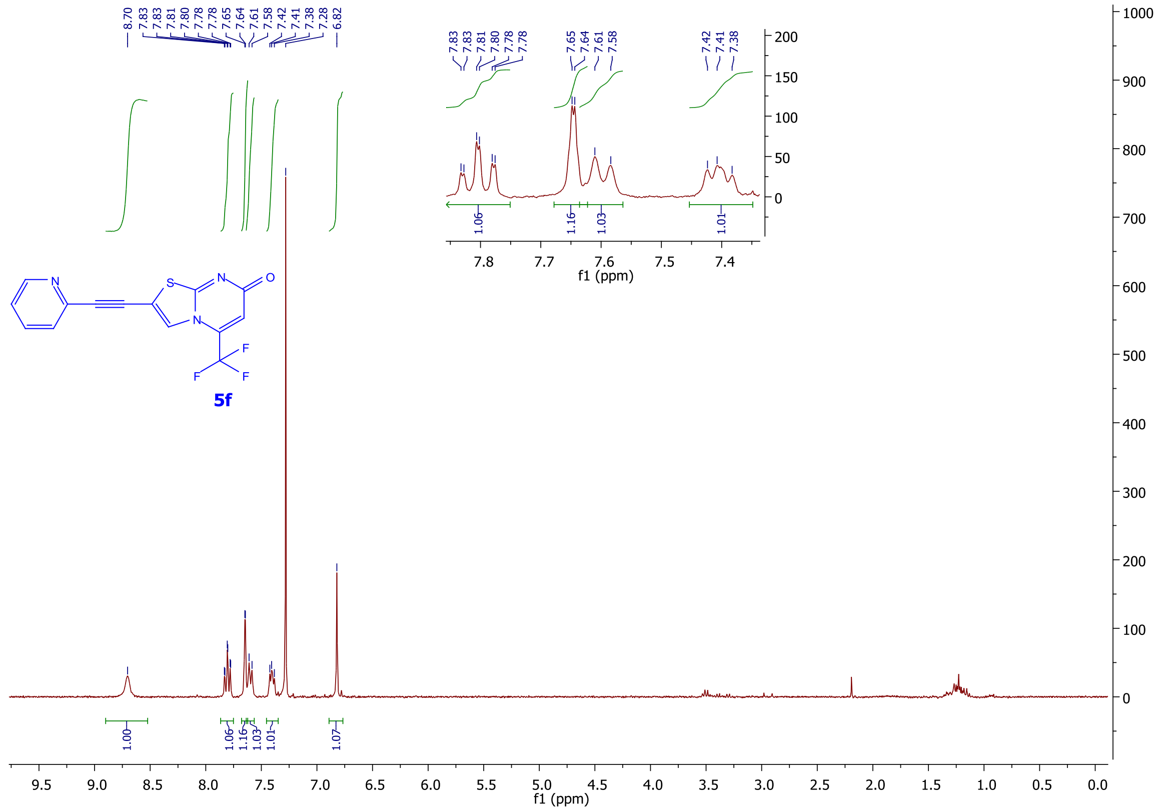

^{19}F NMR (282 MHz, $CDCl_3$)

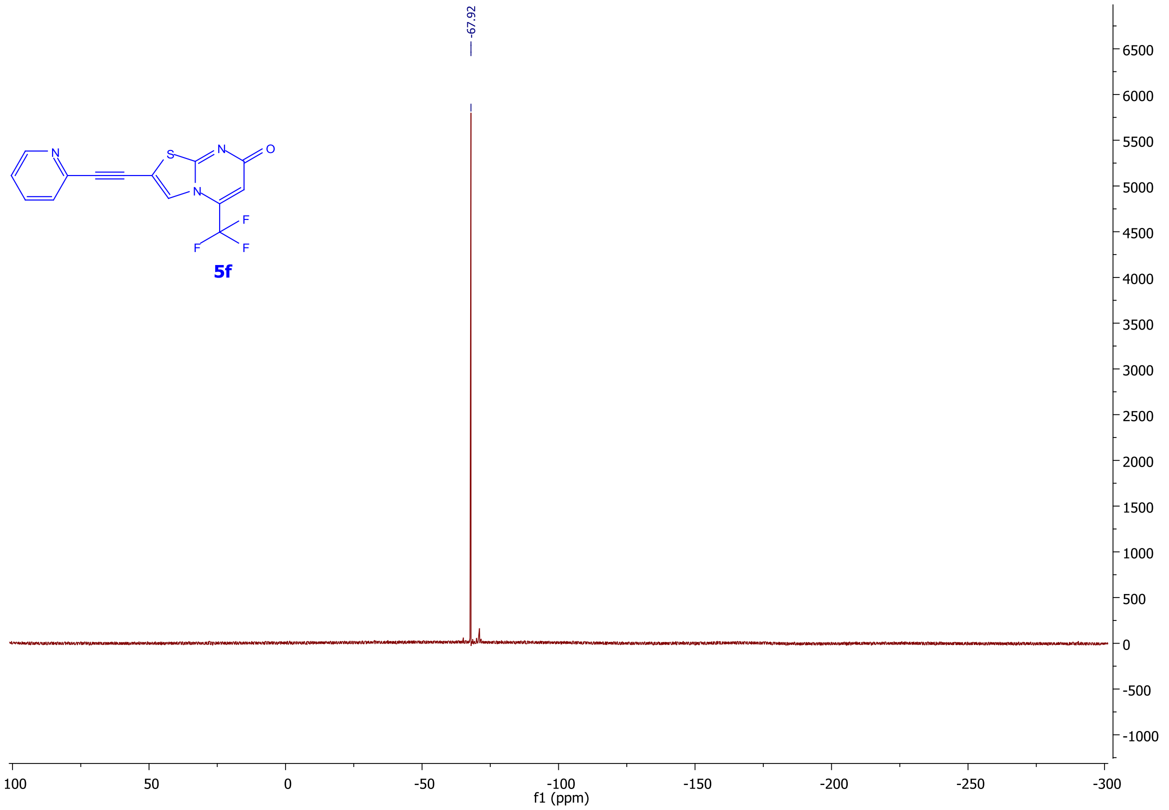

^{13}C NMR (75 MHz, $CDCl_3$)

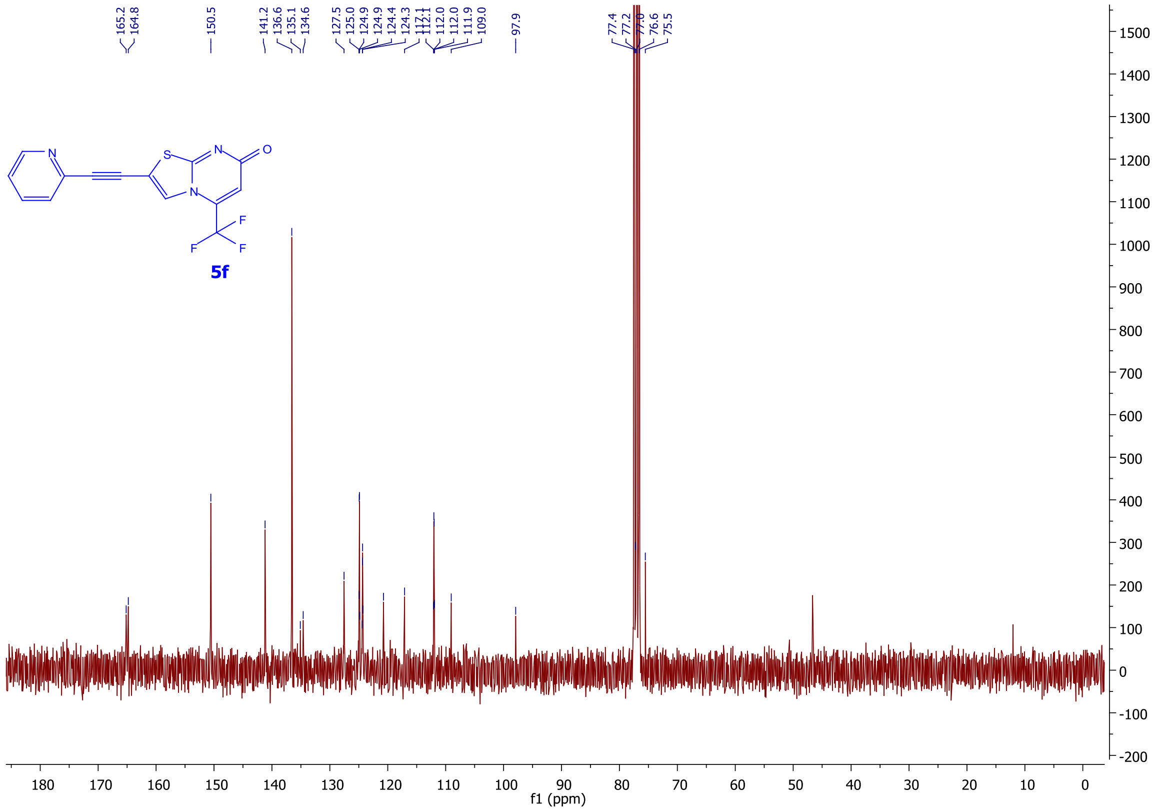

2-(cyclohexylethynyl)-5-(trifluoromethyl)-7*H*-thiazolo[3,2-*a*]pyrimidin-7-one (**5g**)

^{1}H NMR (300 MHz, $CDCl_3$)

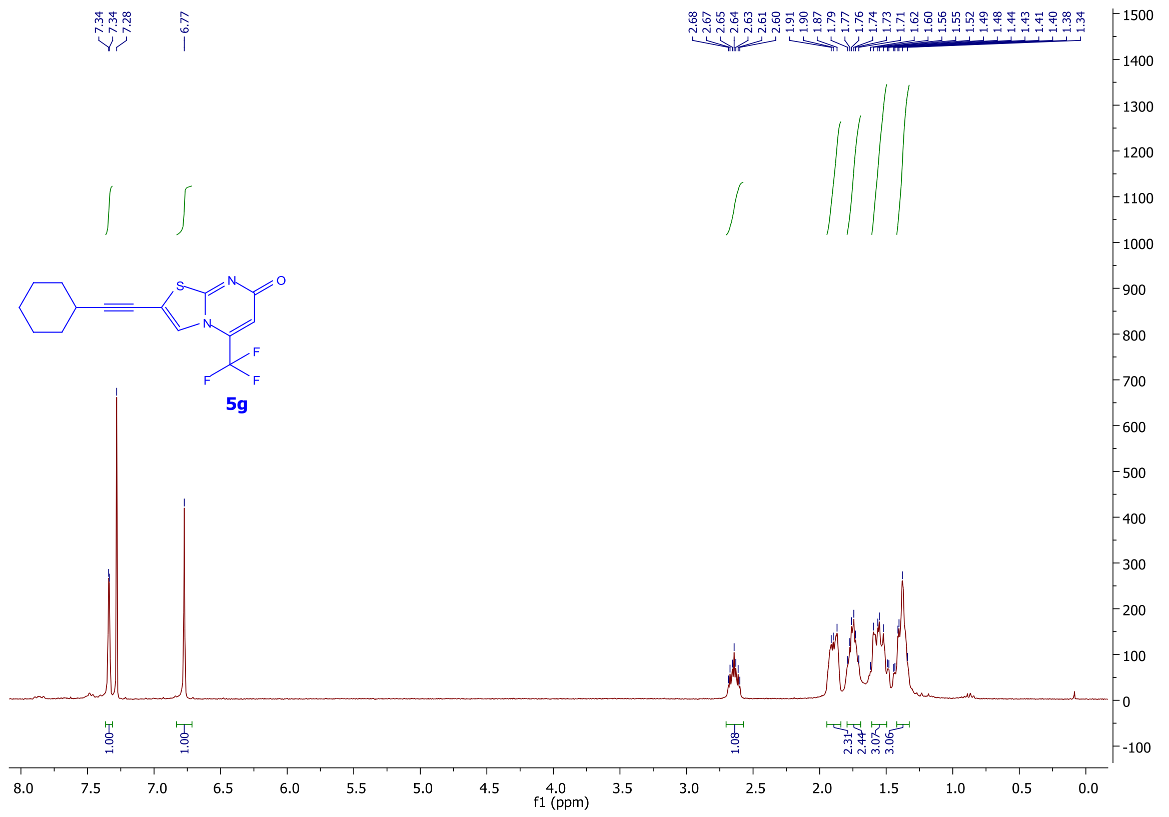

^{19}F NMR (282 MHz, $CDCl_3$)

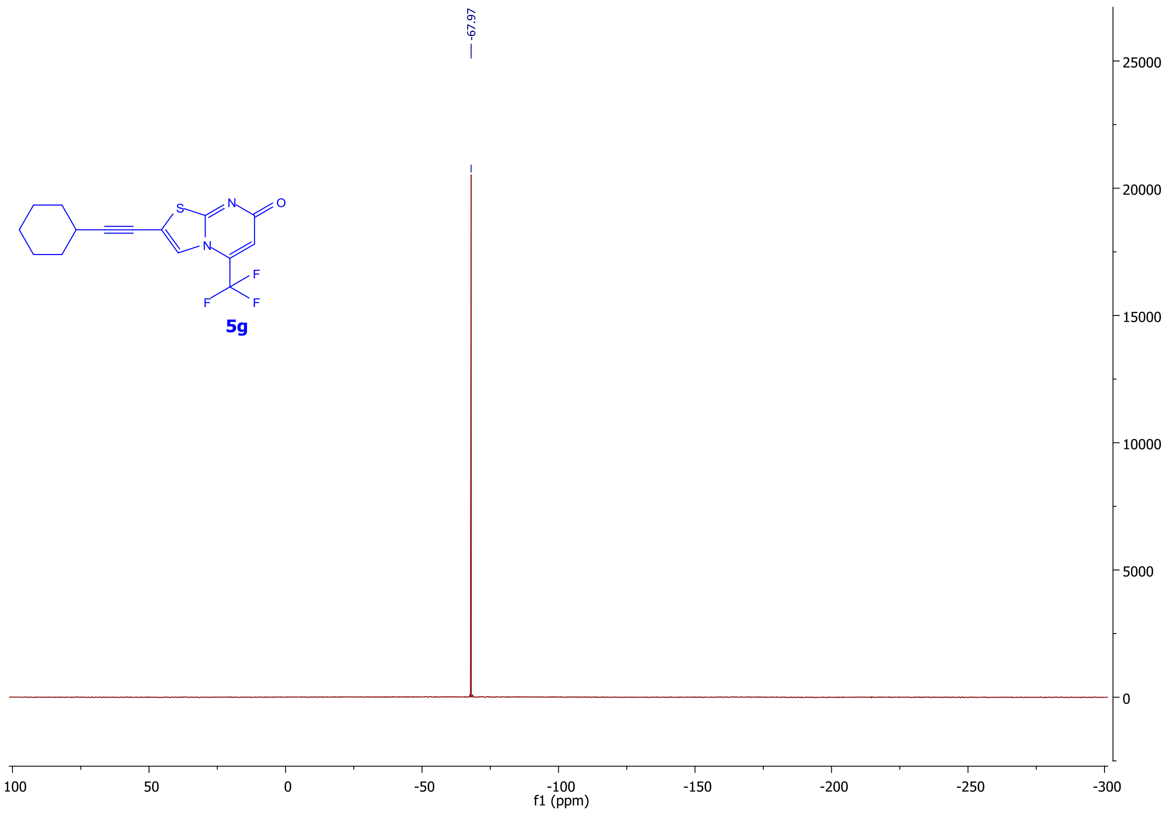

^{13}C NMR (75 MHz, $CDCl_3$)

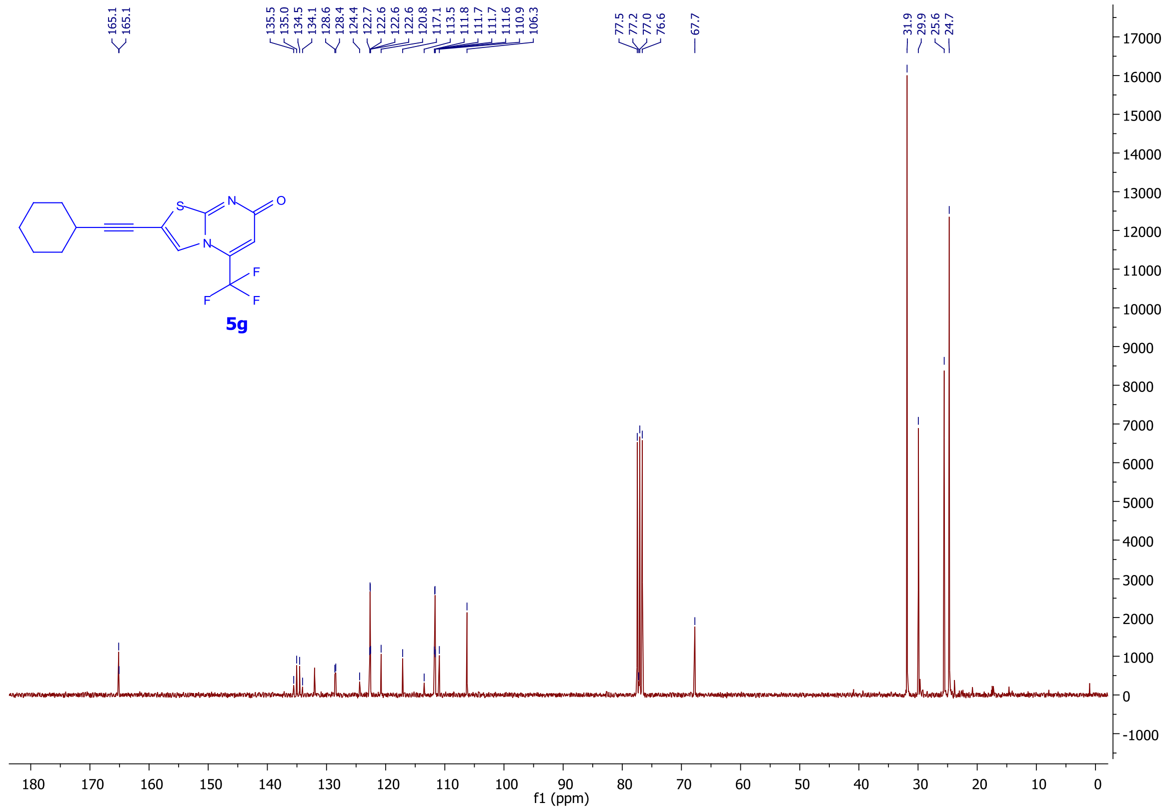

2-(hex-1-yn-1-yl)-5-(trifluoromethyl)-7*H*-thiazolo[3,2-*a*]pyrimidin-7-one (**5h**).

^{1}H NMR (300 MHz, $CDCl_3$)

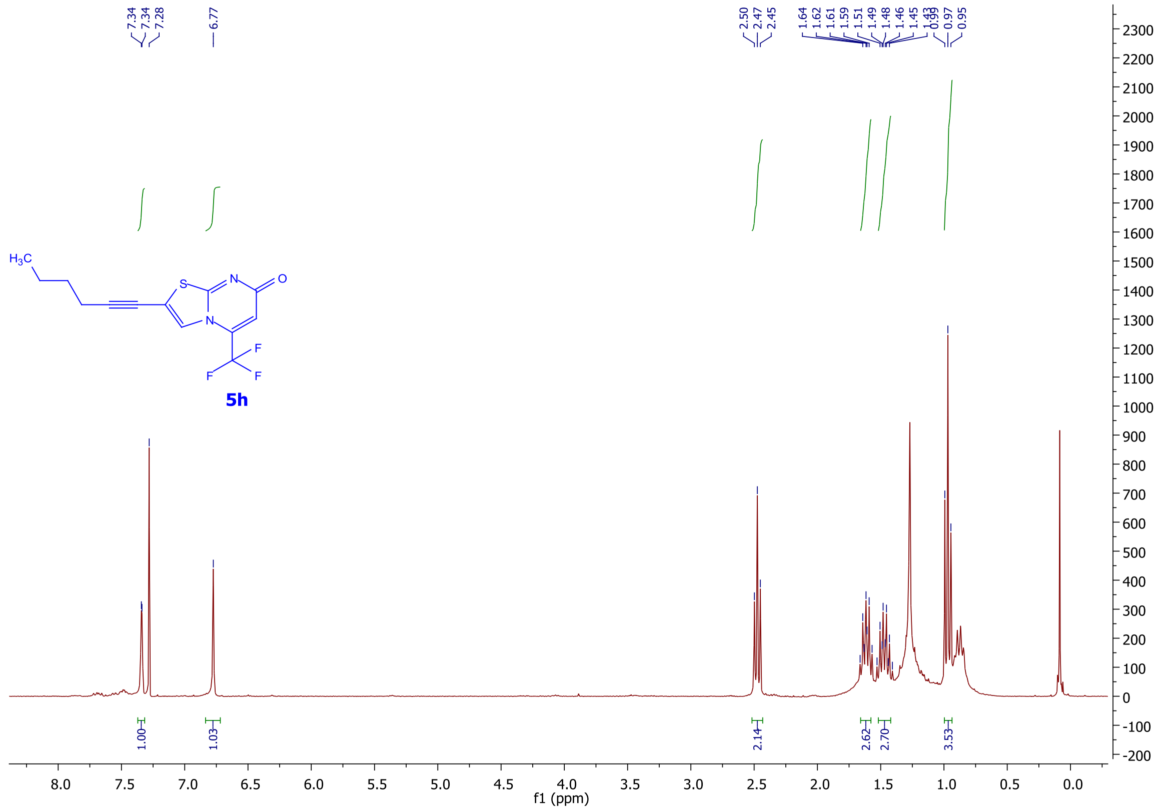

^{13}C NMR (75 MHz, $CDCl_3$)

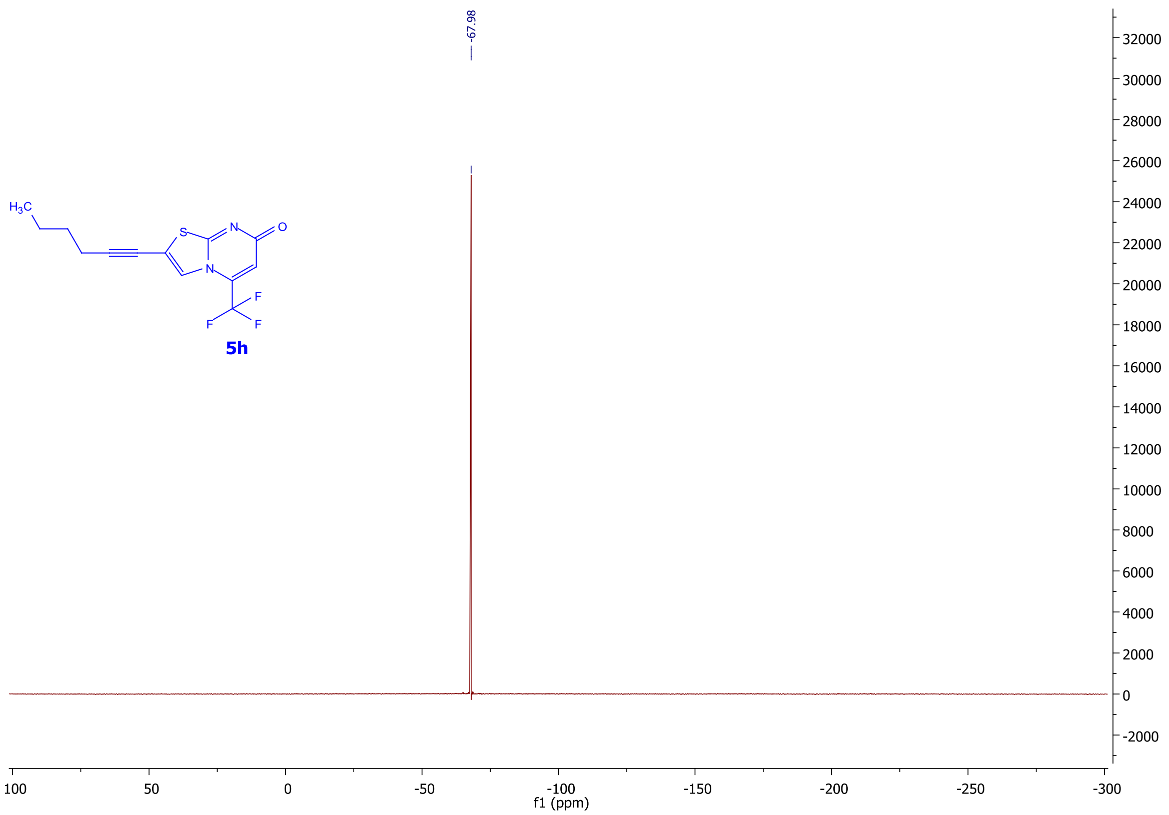

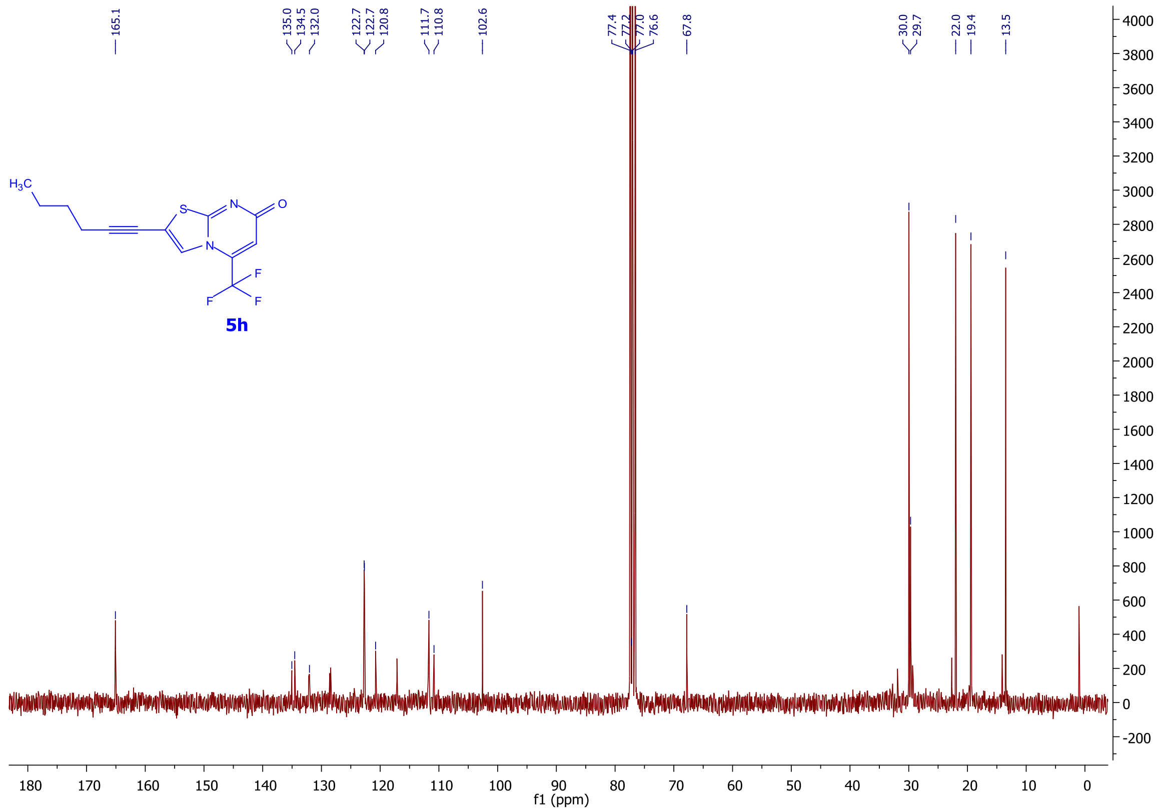

Supplement: Supplementary file 1 [file molecules-27-03013-s001.zip › molecules-1707190-supplementary.pdf]
